# Global Justice Index Report 2022

**DOI:** 10.1007/s41111-023-00240-0

**Published:** 2023-04-11

**Authors:** Yanfeng Gu, Sujian Guo, Xuan Qin, Wen Qu, Zhongyuan Wang, Tiantian Zhang

**Affiliations:** Fudan Institute for Advanced Study in Social Sciences, Fudan University, Shanghai, China

**Keywords:** Global Justice Index, Indicators, Measurements, Methods, Country global rankings

## Abstract

The Global Justice Index is a multiyear research project based at Fudan Institute for Advanced Study in Social Sciences that assesses the contributions made by each country to achieving greater global justice. We have published results from 2010 to 2019 in Global Justice Index Report, Global Justice Index Report 2020, Global Justice Index Report 2021, and we are now presenting our fourth-year results for 2020 in Global Justice Index Report 2022, which is an updated version of previous years’ reports. This year, we take into account changes to global justice influenced by the COVID-19 pandemic. The report consists of four sections: introduction, findings, main results, and conclusion. In the introduction, we discuss the development of the conceptual framework and evaluative principles to justify our selection of the dimensions and indicators for measurement. Next, in the findings section, we report the data, indicators, and our results for each country for each of the 10 issues we identify, and provide regional comparisons for Asia, Europe, North America, Latin America, Africa, and Oceania. In the following section, we present the main results for the global justice indices, and report the ranking of each country’s contribution to achieving greater global justice. In the final section, we discuss the applications and limitations of the index and potential further research trajectories.

## Introduction

The Global Justice Index is an ongoing research project conducted by Fudan-IAS to measure the level of global justice achieved by nation-states. Our goal is to provide readers with an accurate understanding of each country’s contribution to global justice as a whole. We have published results from 2010 to 2019 and are now presenting our fourth-year results for 2020.^1^ This year’s report contains four sections: an introduction, findings, main results, and a conclusion.

The Global Justice Index study for 2020 takes the form of an updated version of previous years’ reports. This year, we take into account changes to global justice caused by the COVID-19 pandemic. In 2020, the global COVID-19 pandemic had a profound effect on global justice.^2^ It intensified economic inequality, widened gender gaps, and increased discrimination against vulnerable populations. A variety of measures have been implemented to promote global justice in response to the COVID-19 pandemic. These include the provision of economic relief to families and businesses, expanding access to healthcare, increasing the use of digital technology to bridge the digital divide, protecting vulnerable populations from discrimination, and strengthening international cooperation. To achieve an accurate measurement of each country’s contribution to greater global justice, we have incorporated novel indicators in certain issue areas (health and humanitarian aids), expanded the number of countries (education), and strengthened our analysis by adding a discussion of the influence of the pandemic. However, our methodology, main indicator system, and data sources remain consistent with those of last year’s report to enable cross-comparison.

In our introduction, we discuss the development of a conceptual framework to justify our choice of issues, dimensions, and indicators for measurement. Although this was covered in previous reports, it is important to repeat it here as part of maintaining the integrity of this year’s Global Justice Index research. Global justice is widely understood to be a complex concept including multiple components belonging to both normative and empirical realities, requiring an integrated theoretical framework that covers these aspects. In our theoretical paper, published in 2019, we clarified our conceptualization of global justice and presented our issue-area system based on it.^3^

Our conceptualization of global justice synthesizes multiple theories and intellectual traditions from different social, cultural, and political contexts. We recognize three main approaches—rights-based, goods-based, and virtue-based—as the foundation for a coherent theoretical framework with a normative basis for measurement. A rights-based approach focuses on the principles, rules, and sources of legitimacy. A goods-based approach concentrates on the material and institutional support provided by governments or institutions. A virtue-based approach considers justice to be something an individual must pursue rather than comply with. The relationship between these three is interdependent, forming one holistic whole. They all work together, as follows: the rights-based conceptualization provides the basic structure (the bones), the goods-based conceptualization provides substantial material support (the muscles), and the virtue-focused conceptualization provides personal motivation and internalized willingness (the heart).

Based on this theoretical framework, we proposed two evaluative principles to better understand and justify the selection of issue areas for evaluation. These are Common but Differentiated and Respective Capabilities (CBDR-RC) and Cosmopolitan but Due-diligent Responsibilities (CDDR). CBDR-RC addresses the issues “for which no single nation-state can be held directly accountable or responsible, matters that can only be tackled through the globally concerted efforts of all stakeholders.”^4^ For example, issues such as climate change require a collective effort on the part of all countries to be adequately addressed, and that this effort cannot be undertaken by one nation alone. The second principle, CDDR, asserts that “all-nation-states are morally obligated to provide cosmopolitan aid, in which context the least advantaged will have a due-diligent responsibility” (Guo et al. 2019). This principle is based on the concept of mutual accountability as proposed in the Paris Declaration on Aid Effectiveness, adopted in 2005 at the Second High-Level Forum on Aid Effectiveness to promote better cooperation between actors in aid and development. According to this principle, anti-poverty and education policies are part of domestic affairs, and nation-states are expected to provide material and institutional assistance to their citizenry within their territories.

Drawing on the principles of CBDR-RC and CDDR, we have selected two clusters of global justice issue areas in our measurement. The issue areas that relate to CBDR-RC are (1) climate change (global warming), (2) peacekeeping, (3) humanitarian aid, (4) terrorism and armed conflict, (5) cross-national criminal police cooperation, and (6) refugees. The issue areas that relate to CDDR are (7) anti-poverty, (8) education, (9) public health, and (10) the protection of women and children. In the following sections, we provide rankings for individual nations’ contributions to global justice across these 10 issue areas for 2020. We also provide regional comparisons, detailed policy analysis, and visualization tools to enable a more accurate understanding of each country’s contribution to achieving global justice.

## Findings

### Issue 1: Climate Change

#### Introduction

Due to the rapid development of modern economies and the acceleration of global economic integration, the environmental problems caused by climate change, such as acid rain, the greenhouse effect, desertification, and sea-level rise, have become increasingly prominent and are understood to seriously threaten human survival and development.^5^ Climate change is taking place on a global scale, and it is substantially affected by human activity, in particular increasing greenhouse gas emissions.^6^^,^^7^ The warming of the atmosphere is accelerating and could cause a deterioration in living conditions and to global economic and social losses.^8^

The events of 2020 were unprecedented for people and the planet, bringing two challenges to modern civilization, i.e., the COVID-19 pandemic, a disaster on a scale not seen for more than a century, and increasing climate change and accompanying phenomena. Statistical data collected from the NASA Earth Observatory^9^ and the Global Carbon Project^10^ report that although global GHG emissions fell by about 7% in 2020 during the COVID-19 pandemic, this change did not affect global climate. The climate is changing in one main respect: the world is getting hotter. The earth’s mean surface temperature for 2020 was 1.2 ± 0.1 °C above the 1850–1900 baseline, and it was one of the three warmest years on record globally.^11^

As a core element of the Sustainable Development Goals (SDGs),^12^ urgent action is called for to combat climate change and its impacts by nearly 200 countries. Multilateral climate governance has been put into practice by a variety of means. One landmark is the Paris Agreement, which has the central aim of keeping the increase in global temperature well below 2 °C over the pre-industrial baseline levels in this century and to pursue efforts to limit the temperature increase even further, to 1.5 °C. To comply with this treaty on, individual countries set a global emissions-reduction target and submitted their plans for climate action, referred to as nationally determined contributions (NDCs).

Taking into account the principle of Common but Differentiated and Respective Capabilities (CBDR-RC) as proposed by this project,^13^ this study measures the performance of each country in addressing climate change in terms of global justice.

#### Dimensions and Indicators

Consistent with the 2021 Global Justice Index Report in 2021,^14^ we assess the contribution and performance of individual countries with respect to their promotion of global justice in the issue area of climate change, investigating energy consumption, electricity production, carbon dioxide (CO_2_) emissions, and forest coverage (Table 1). The indicators were determined as follows.

**Table 1:** Data on climate change

Category	Dimension	Indicator	Data source	Coverage
Performance	Energy consumption	Total primary energy consumption	BP Statistical Review of World Energy	75 countries
		Primary energy consumption per capita		
		Oil consumption		
		Natural gas consumption		
		Coal consumption		
	Electricity production	Total electricity production		
		Electricity production from nuclear sources		
		Electricity production from hydroelectric sources		
		Electricity production from renewable sources excluding hydroelectric		
	CO_2_	CO_2_ emissions	Global Carbon Project	
		CO_2_ emissions per GDP		
		CO_2_ emissions per capita		
	Forest	Total forest area	UNEP	
		Rate of change in forest area		
		Forest area per capita		
		Forest coverage		
		Planted forest area		

The global warming that has occurred since the pre-industrial era has been primarily attributed to increases in atmospheric CO_2_ concentrations, mainly resulting from the carbon emissions of fossil fuel combustion.^15^ The massive energy consumption due to this combustion poses serious threats to energy security, environmental quality, climate change, and human health. Five alternative proxies (total, primary energy consumption per capita, oil consumption, natural gas consumption, and coal consumption) are used to measure the dimension of energy consumption. We draw on the energy consumption data from the BP Statistical Review of World Energy 2020.

CO_2_ is produced as a waste product. It absorbs the heat radiation from the Earth’s surface, which warms the surface. Thus, the energy consumption and CO_2_ emissions rate mirrors a country’s influence on global warming. The CO_2_ dimension indicators contain CO_2_ emissions, CO_2_ emissions per unit GDP, and CO_2_ emissions per capita. For this report, CO_2_ data are drawn from the Global Carbon Project to maintain data consistency.

Overall, 25% of emissions come from the production of electricity and combustion for heat.^16^ Policies should be developed to shift energy systems away from fossil fuels that produce greenhouse gas (GHG) and toward renewable energies. Thus, it is vital to include electricity generation in the dimension system to access performance in mitigating global climate change. Accordingly, in this dimension, the indicators are electricity production overall, electricity production from nuclear sources, electricity production from hydroelectric sources, and electricity production from renewable sources other than hydroelectric ones. As with energy consumption, electricity production data for this report are extracted from the BP Statistical Review of World Energy 2020.

As the lungs and major climate regulators, forests play a vital role in determining the balance of CO_2_ in the atmosphere. Through their photosynthesis, forests take up and store nearly 1/3 of human CO_2_ emissions. The process of afforestation represents individual countries’ efforts to fight climate change. It is significant to use the forest dimension in this issue. The data are collected from the UN Environment Program (UNEP), and five indicators are selected: forested area in total, rate of change in forested area, forested area per capita, forest coverage, and planted forest area. Because 2019 data for forest area change rate, forested area per capita, and forest coverage are not yet available, we supplement using values based on the data for forested areas. The population data used when calculating forested area per capita are from the World Bank. It is worth noting that data for planted forest area are only available through 2017, but taking into account that the data do not greatly fluctuate from year to year, we used records from the previous years to impute and estimate 2020 data.

In accordance with last years’ report, we measure 75 countries’ performance of global justice in the issue of climate change in 2020.

#### Results

Adopting the index construction method developed for this project, this section reports the ranking results for the selected 75 countries’ performance in terms of global justice from a climate change perspective in 2020. Table 2 presents the detailed rankings.

**Table 2:** Country rankings in the climate change aspect of promoting global justice in 2020

Country	Ranking	Country	Ranking
Brazil	1	Belarus	39
Canada	2	Thailand	40
Russian Federation	3	Malaysia	41
Sweden	4	Venezuela	42
China	5	Greece	43
France	6	Ireland	44
Finland	7	Bangladesh	45
Peru	8	Denmark	46
Latvia	9	Poland	47
Japan	10	Czech Republic	48
United States of America	11	Australia	49
Colombia	12	Morocco	50
Vietnam	13	Hungary	51
Turkey	14	Belgium	52
Spain	15	Luxembourg	53
India	16	Cyprus	54
Chile	17	Algeria	55
Italy	18	Argentina	56
Switzerland	19	Uzbekistan	57
New Zealand	20	Netherlands	58
Slovenia	21	Egypt	59
Philippines	22	Israel	60
Germany	23	Iceland	61
Norway	24	Ukraine	62
Ecuador	25	Iraq	63
Austria	26	Pakistan	64
Estonia	27	South Africa	65
Sri Lanka	28	Kazakhstan	66
Indonesia	29	Oman	67
Portugal	30	Iran	68
Mexico	31	United Arab Emirates	69
Lithuania	32	Singapore	70
Bulgaria	33	Kuwait	71
Azerbaijan	34	Turkmenistan	72
Romania	35	Saudi Arabia	73
Slovakia	36	Trinidad and Tobago	74
United Kingdom of Great Britain and Northern Ireland	37	Qatar	75
Republic of Korea	38		

As shown in Table 2, the highest ranking countries are very similar to those in the 2019 ranking, with small changes, including that Russia has risen from fourth to third place, while Sweden has fallen from third to fourth, and Colombia has dropped out of the top 10, while Latvia has risen from eleventh to ninth. Of the top 10 countries, five are located in Europe, two are in each of Asia and Latin America respectively, and one is in North America. Here, Brazil, Russia, China, Peru, and Latvia are considered developing countries, while Canada, Sweden, France, Finland, and Japan are developed countries. It can be easily seen that developing countries are on the front lines of the battle with climate change.

The bottom-ranked countries are Kazakhstan, Oman, Iran, the United Arab Emirates, Singapore, Kuwait, Turkmenistan, Saudi Arabia, Trinidad and Tobago, and Qatar, most of which are developing countries in Asia and Latin America. Singapore is the only developed country among them, appearing here due to the continuous reduction in forest area and the very limited decrease in energy consumption and CO_2_ emissions, in a context where other countries have markedly reduced their energy consumption and emissions. The names of these 10 countries has not changed, but their rankings have changed slightly, indicating the steadiness and confidence level of the index calculation method.

#### Regional Analysis

**Fig. 1:**
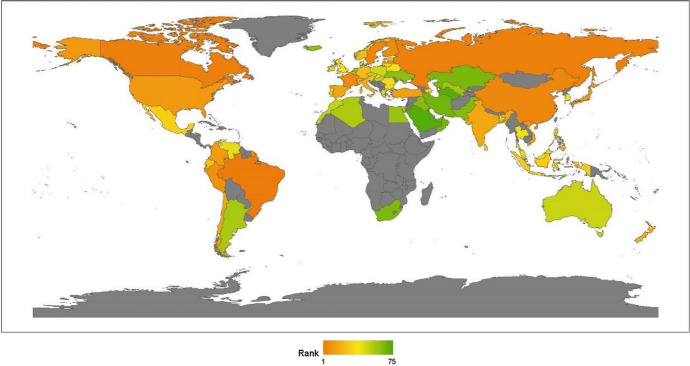
2020 index ranking for climate change on a world map

We classify countries by continent, namely, Asia, Europe, North America, Latin America, Africa, and Oceania. This section provides a regional analysis of the rankings in the issue of climate change, obtained by calculating the average scores for the countries by continent. Comparing the 2020 results with those for 2019, given in our last annual report, we can see that the rankings are unchanged: North America, Latin America, Europe, Oceania, Asia, and Africa (see Fig. 1).^17^

Asia: The top five Asian countries by performance on climate are China, Japan, Vietnam, Turkey, and India. As one of the largest emitters of CO_2_ worldwide,^18^ China has committed to peak its carbon emissions by 2030 and attain carbon neutrality by 2060. China is ranked fifth in tackling climate change since 2017, with its main advantages residing in its power production matrix and forest. China has developed a series of domestic strategies^19^ and policies, including abated coal consumption,^20^ cleaner energy development,^21^ nationwide ecological restoration,^22^ and other various decarbonization technologies^23^ as potential countermeasures to achieve carbon neutrality by 2060.

Japan ranks ninth in the dimensions of electricity consumption and forest. Following the accident at the Fukushima-Daichi Nuclear Power Station, hydroelectric power has been among the few self-sufficient sources of energy in resource-poor Japan, and liquefied natural gas has been promoted for the long term to lessen emissions.

Vietnam’s ranking rose from twentieth in 2019 to thirteenth in 2020. With the aim of increasing resilience to climate change, Vietnam has adopted ecological redlining to curb the expansion of agriculture into forested areas. From 2011 to 2021, Vietnam’s forest area grew by over a million hectares. After a string of deadly typhoons in late 2020, Vietnam’s prime minister also called for the country to plant 1 billion trees nationwide by 2025 to reduce the risk of landslides and flooding.

Turkey performed better on climate in 2020 than in previous years, with above-average scores in the electricity generation and forest dimensions. Although coal was most commonly used for electricity production in Turkey, amounting to 34.5% of the total electricity generation, hydroelectricity power ranked second, with 25.5%. From 2000 to 2020, Turkey experienced a net change of 88.8 kha (0.65%) in tree cover.

India saw its ranking rise to sixteenth in 2020, with the fifth highest score in the dimension of electricity. Its power mix consisted of 57.9% fossil fuels and 42.3% non-fossil fuels (including 29.2% wind, solar, and other recyclable fuels and 11.5% hydro power).

Indonesia’s ranking fell from the top five in Asia due to its reduced forest area, with the loss of 115,459 ha of forest cover in 2020. Therefore, commitments by the government and the private sector to maintaining zero-deforestation rate should be upheld, and the environment should not be sacrificed for economic growth.

The bottom countries are Singapore, Kuwait, Turkmenistan, Saudi Arabia, and Qatar. Singapore’s ranking declined from sixty-fifth in 2019 to seventieth in 2020, with its rankings decreasing across all dimensions. Oil has a dominant share in Singapore’s total energy consumption, reaching 70% in 2020. From 2000 to 2020, Singapore experienced a net change of − 379 ha (− 1.9%) in tree cover. In response, it launched the One Million Trees project, involving restoration of both inland and mangrove forests. Singapore ratified the Paris Agreement in 2016 and submitted its first NDC, declaring a target reduction of GHG emissions intensity by 36% from 2005 levels by 2030, with the aim of achieving peak emissions around 2030. Kuwait, Turkmenistan, Saudi Arabia, and Qatar, which are traditionally oil exporters, received lower rankings than other Asian countries, as they had higher CO_2_ emissions per capita and carbon intensity as well as lower forest reserves.

Europe: European countries performed better than those of Oceania, Asia, and Africa on the climate issue. The top-ranking European countries are Russia, Sweden, France, Finland, and Latvia, while the lowest ranking countries are Luxembourg, Cyprus, the Netherlands, Iceland, and Ukraine, similar to the rankings for the previous year. Russia’s ranking rose from fourth in 2019 to third in 2020, with its performance improving in the dimensions of CO_2_ emissions, energy consumption, and electricity generation. In 2020, the total amount of forest area in Russia did not change relative to the previous year. In Russia, CO_2_ emissions in 2020 were 1797.6 megatons, with a 4.47% decrease from 2019. Russia’s per capita energy consumption reached 243 gigajoules in 2020, down from 266 gigajoules in the previous year. Roughly 60% of Russia’s electricity is generated by fossil fuels (mainly using natural gas), 20% by hydroelectricity, and 20% by nuclear reactors, making a relatively clean mix. The better performing countries had at least one strength across all dimensions, whereas the countries with poor rankings scored lower in all dimensions.

Notably, Norway’s and Germany’s rankings fell from eighteenth and thirteenth in 2019 to twenty-fourth and twenty-third in 2020, respectively, with their rankings decreasing significantly in relation to forest. Germany’s forests have been greatly damaged by extreme weather events such as droughts and heat waves, while Norway’s total forest cover is witnessing losses every year due to deforestation. Ireland’s ranking rose from fifty-first in 2019 to forty-fourth in 2020, with its performance improving in the dimension of CO_2_ emissions. In 2020, Ireland’s national GHG emissions amounted to 58.77 megatons, or 3.6% lower than 2019 emissions.

North America: North America is the leading region in the 2020 climate rankings, as in 2019. Canada ranks second worldwide, and the United States ranks eleventh. Both Canada and the United States have excellent performance in electricity generation and forest cover, with a sizable proportion of renewables in power production and considerable forest coverage. In 2020, the CO_2_ emissions of the United States and Canada fell by 11% and 8.9% respectively. The two countries’ energy consumption also decreased, largely driven by the COVID-19 restrictions, such as the lower use of transportation, decreased electricity demand, and halted industrial activities. However, the United States has rolled back certain environmental regulations and is poised to direct stimulus funds toward reinvigorating the fossil fuel industry. This suggests that keeping the climate healthy in the post-COVID-19 era may be a serious challenge.

Latin America: Latin America ranked second among all continents. The top three countries here are Brazil, Peru, and Colombia, while the bottom countries are Venezuela, Argentina, and Trinidad and Tobago, as was the case in 2019.

Brazil shows a high score in the dimensions of power production mix and forest. Its performance benefits from its reliance on hydropower for electricity generation. Although illegal mining activity and cattle ranching have led to deforestation in Brazil, the abundant endowment of forest contributes to its higher score. Peru and Columbia continue their excellent performance of controlling emissions and energy consumption, partly resulting from restrictions imposed due to the COVID-19 pandemic, including closure of industries and commercial establishments.

Venezuela’s score in the dimension of emissions was relatively low, which dragged down its coverall ranking. Argentina scored very low in the forest dimension. From 2000 to 2020, Argentina showed a net change of − 3.56 Mha (− 10%) in tree cover. Trinidad and Tobago ranked seventy-fourth in 2020, as in 2019. As a small island developing state, Trinidad and Tobago has been vulnerable to temperature increases, changes in precipitation, sea-level rise, tropical maritime air patterns, flooding, modified moist equatorial climate, hillside erosion, and loss of coastal habitats. The leading Caribbean producer of oil and gas, Trinidad and Tobago submitted its first NDC in 2018, promising a reduction objective for overall emissions from the power generation, transportation, and industrial sectors by 15% in the business-as-usual scenario by 2030. Taking action to implement climate change mitigation policies in the country is considered a necessity from a mitigation and adaptation perspective, and Trinidad and Tobago is committed to playing its part as a responsible member of the global community.

Africa: Africa as a whole had the worst scores among all continents in the issue of climate in 2020. Four countries are here selected to represent its overall performance: Morocco, Algeria, Egypt, and South Africa. The rankings of these countries did not fluctuate obviously in 2020. Morocco ranked fiftieth and performed poorly in terms of electricity generation and forest cover. Fossil fuels accounted for nearly 70% of Morocco’s electricity generation mix, and the pandemic continues to disrupt the renewable energy sector. To boost reforestation, a total of 600,000 ha of forest plantations are prepared by 2030 as part of the new “Forests of Morocco 2020–2030” strategy. Algeria confronted the same situation. Countries in equatorial areas are becoming increasingly uninhabitable. Egypt ranked fifty-ninth with a below-average score in the dimension of energy consumption and a very poor score in the forestry dimension.

Oceania: Within Oceania, the two largest countries, Australia and New Zealand, ranked forty-ninth and twentieth, respectively, in their climate responses. With a poor score in the dimensions of CO_2_ emissions and energy consumption, Australia experienced increasing temperature, continuous wildfires, and above-average rainfalls through 2020. To cut down on emissions and improve environmental quality, support for new fossil fuel projects needs to be abandoned. New Zealand’s total new planting estimate for 2020 was 34,000 ha. Thanks to relentless reforestation efforts, New Zealand rose from twenty-eighth place in 2019 to twentieth in 2020. In addition, New Zealand has declared a climate change emergency and committed to a carbon–neutral government by 2025.

#### Conclusion

Assuming that worldwide carbon emissions continue at the current rate, global warming is likely to exceed 1.5 °C between 2030 and 2052, even more than 3–5 °C at the end of the twenty-first century.^24^ If dramatic action is not taken within the next decade, we could face irreversible damage to the natural world.

Developing countries are at the front line of the battle. Those parts of the globe that will suffer the most and the soonest are not those that are responsible for putting the CO_2_ into the atmosphere in the first place. Climate change is having a more negative impact on the least-developed and most-vulnerable states, which lack an adaptive capacity because of poverty, marginalization, and geographic isolation. Improved global climate governance and providing sufficient climate aids will deliver a range of livelihood and environmental benefits to enable to adapt to climate change.

The climate and COVID-19 crises are global and unprecedented in their level of disruption, and they require coordinated responses by policy-makers, businesses, and society as a whole. However, while nations are marshaling massive resources to mitigate the economic and social impacts of COVID-19, they may at the same time be missing the chance to address climate change. According to statistical data collected from the NASA Earth Observatory^25^ and the Global Carbon Project,^26^ global GHG emissions fell by about 7% in 2020 during COVID-19. Among the 75 countries in our analysis, CO_2_ emissions were decreased in 2020. The worldwide lockdowns due to the COVID-19 pandemic have reduced air pollution and as a result of lower use of transportation, electricity generation, and industrial production. Emissions are expected to reach even higher levels once the global economy begins to recover from the pandemic.

Countermeasures to mitigate climate change should be undertaken by governmental and regulatory bodies for cleaner energy development, decarbonization technologies, and the acceleration of afforestation to resolve the crisis. Climate concerns could be addressed in part by investing in ecological and green projects, properly disposing of medical wastes, building health-ensuring and livable societies, and terminating the funding of pollution. For consumers, it is vital to avoid a wasteful lifestyle by purchasing fewer physical products and choosing higher quality and lasting ones. These factors will provide a strong foundation for building safer, healthier, and environmentally friendly societies for generations to come.

### Issue 2: Peacekeeping

#### Introduction

Peacekeeping, as defined by the United Nations (UN), is a way that countries torn by conflict can be helped to create conditions for sustainable peace. UN peacekeepers, including military, police, and civilian personnel from many countries, monitor and observe peace processes as they emerge in post-conflict situations and assist ex-combatants to implement the peace agreements that they have signed. UN peacekeeping missions are called upon not only to monitor ceasefires and implement peace agreements but also to protect civilians and reduce one-sided violence.^27^ Overall, UN peacekeeping is a cost-effective approach to guarantee global security, mitigating violent conflicts and reducing the numbers of casualties,^28^ and obtaining a better chance of lasting peace.

After the first blue-helmeted UN troops were deployed in 1948, the UN Security Council has authorized 70 peacekeeping missions and deployed more than one million peacekeepers from 110 nations to help countries navigate the difficult path from conflict to peace. In 2020, there were 21 active multilateral peace operations conducted by the UN. Conflicts and tensions persisted in the Democratic Republic of the Congo, South Sudan, Abyei, Lebanon, and beyond. The Security Council closely monitors ongoing conflicts in Africa, Latin America, and the Middle East, including the situation in Libya, the Syrian Arab Republic, and Yemen as well as the Palestinian issue, which continues to have serious repercussions throughout the sub-region.

In 2020, the first year of the COVID-19 pandemic, wide-ranging impacts were seen on multilateral peace operations. The crisis affected all operations simultaneously, including those of host nations, headquarters, and contributing countries. It caused major disruption, from the political-strategic level where mandates are drawn up down to the operational and tactical levels. Operations were forced to adapt to preserve continuity as far as possible. The pandemic highlights two particular areas of deeper tension in UN peacekeeping: the risk of unintended harm and the impact of financial constraints on peacekeeping operations, posing existing global governance questions with renewed urgency. Thus, UN peacekeeping has had to seek ways of continuing to deliver on their priority mandates while mitigating the challenges posed by the pandemic.

Global justice in peacekeeping worldwide requires awareness of collaboration and commitment to this issue. Our Global Justice Index measures each country’s personnel and financial contributions and efforts in support of global peacekeeping.

#### Dimensions and Indicators

As previously, we measured the contribution of 193 nation states in this issue through two indicators, including personnel and financial contributions to quantify each country’s efforts in maintaining global peace in 2020. For personnel contribution, we used the number of troops and police deployed to measure the commitment of manpower inputs. The deployment of trained troops and police officers helps reduce crime, instability, and fragility in countries where they are engaged. More peacekeepers in conflict areas are correlated with fewer civilian deaths and lower levels of violence. For financial contribution, we employ the composition of the levels of donations for peacekeeping operations made by each country to the UN as the indicator. As shown in Table 3, these data come from the UN peacekeeping website and from the International Peace Institute.

**Table 3:** Data on peacekeeping

Category	Dimension	Indicator	Data source	Coverage
Contribution	Personnel Contribution	Troops and Police	UN Peacekeeping Website International Peace Institute	193 countries
	Financial Contribution	Donation		

#### Results

This section reports the rankings of 193 countries in 2020 based on their level of contribution to global justice in the issue area of peacekeeping. Table 4 shows the ranking results for 193 countries in 2020.

**Table 4:** Country rankings in the peacekeeping aspect for promoting global justice in 2020

Country	Ranking	Country	Ranking
China	1	Paraguay	98
United States of America	2	Bosnia and Herzegovina	99
Bangladesh	3	Bolivia (Plurinational State of)	100
Ethiopia	4	Bhutan	101
Rwanda	5	Slovenia	102
Nepal	6	Mali	103
India	7	Colombia	104
Pakistan	8	Croatia	105
Egypt	9	Iran (Islamic Republic of)	106
Indonesia	10	Samoa	107
Ghana	11	Luxembourg	108
France	12	Estonia	109
Senegal	13	Oman	110
Germany	14	Ecuador	111
Morocco	15	Malta	112
United Republic of Tanzania	16	Honduras	113
Japan	17	Kyrgyzstan	114
Italy	18	Madagascar	115
United Kingdom of Great Britain and Northern Ireland	19	Bahrain	116
Chad	20	Cyprus	117
Togo	21	Republic of Moldova	118
Burkina Faso	22	Algeria	119
Spain	23	Belarus	120
Uruguay	24	Dominican Republic	121
South Africa	25	Iceland	122
Republic of Korea	26	Tajikistan	123
Cameroon	27	Iraq	124
Zambia	28	Trinidad and Tobago	125
Niger	29	Cuba	126
Guinea	30	Bulgaria	127
Mongolia	31	Latvia	128
Malaysia	32	Azerbaijan	129
Malawi	33	Bahamas	130
Russian Federation	34	Timor-Leste	131
Cambodia	35	The former Yugoslav Republic of Macedonia	132
Burundi	36	Costa Rica	133
Mauritania	37	Monaco	134
Canada	38	Montenegro	135
Sri Lanka	39	Lebanon	136
Uganda	40	Liechtenstein	137
Ireland	41	Panama	137
Côte d’Ivoire	42	Albania	139
Australia	43	Libya	140
Jordan	44	Turkmenistan	141
Benin	45	Uzbekistan	142
Gabon	46	Andorra	143
Sweden	47	Equatorial Guinea	144
Brazil	48	Barbados	145
Austria	49	Botswana	145
Netherlands	50	Papua New Guinea	147
Argentina	51	Democratic Republic of the Congo	148
Fiji	52	Guinea-Bissau	149
Portugal	53	Mauritius	150
Finland	54	Syrian Arab Republic	150
Ukraine	55	San Marino	152
Nigeria	56	Georgia	153
Thailand	57	Jamaica	153
Switzerland	58	Democratic People’s Republic of Korea	155
El Salvador	59	Angola	156
Poland	60	Myanmar	156
Slovakia	61	Nicaragua	156
Serbia	62	Sudan	156
Saudi Arabia	63	Suriname	156
Peru	64	Yemen	156
Norway	65	Maldives	162
Belgium	66	Afghanistan	163
Gambia	67	Seychelles	164
Turkey	68	South Sudan	164
Greece	69	Lao People’s Democratic Republic	166
Denmark	70	Antigua and Barbuda	167
Guatemala	71	Eswatini	167
Djibouti	72	Guyana	167
Romania	73	Mozambique	167
Tunisia	74	Saint Kitts and Nevis	167
Kenya	75	Haiti	172
Congo	76	Belize	173
Kazakhstan	77	Cabo Verde	173
United Arab Emirates	78	Dominica	173
Liberia	79	Grenada	173
Israel	80	Marshall Islands	173
Singapore	81	Micronesia (Federated States of)	173
Zimbabwe	82	Nauru	173
Sierra Leone	83	Palau	173
New Zealand	84	Saint Lucia	173
Vietnam	85	Saint Vincent and the Grenadines	173
Mexico	86	Tonga	173
Qatar	87	Central African Republic	184
Czechia	88	Comoros	184
Kuwait	89	Eritrea	184
Chile	90	Kiribati	184
Venezuela (Bolivarian Republic of)	91	Lesotho	184
Lithuania	92	Sao Tome and Principe	184
Hungary	93	Solomon Islands	184
Namibia	94	Somalia	184
Philippines	95	Tuvalu	184
Brunei Darussalam	96	Vanuatu	184
Armenia	97		

The rankings of the top 10 countries remain almost the same with 2019. China, the United States, Bangladesh, Ethiopia, Rwanda, Nepal, India, Pakistan, Egypt, and Indonesia made outstanding contributions to worldwide peacekeeping in 2020. Of these, one is in North America, six are in Asia, and three are in Africa. Bangladesh rose to third place, largely due to its increased contribution of peacekeepers, from 77,776 in 2019 to 78,838 in 2020, with a focus on the Democratic Republic of the Congo, South Sudan, Mali, and Central African Republic. Ethiopia fell to fourth place, due to a downturn in the scale of its peacekeeping forces, from 85,913 in 2019 to 78,652 in 2020. India also withdrew its troops dispatched overseas, with a reduction of 13%; thus India ranked seventh in 2020, down from sixth in 2019, retaining a major peacekeeping force in South Sudan, the Democratic Republic of the Congo, and Lebanon.

The countries at the bottom rungs are the Central African Republic, Comoros, Eritrea, Kiribati, Lesotho, Solomon Islands, Somalia, Sao Tome and Principe, Tuvalu, and Vanuatu. The lowest ranking countries for 2020 with respect to peacekeeping are sparely populated and less-developed countries, with low ability to participate in peacekeeping operations. The contribution of these countries’ contributions to peacekeeping are restricted due to their lack of capability.

#### Regional Analysis

**Fig. 2:**
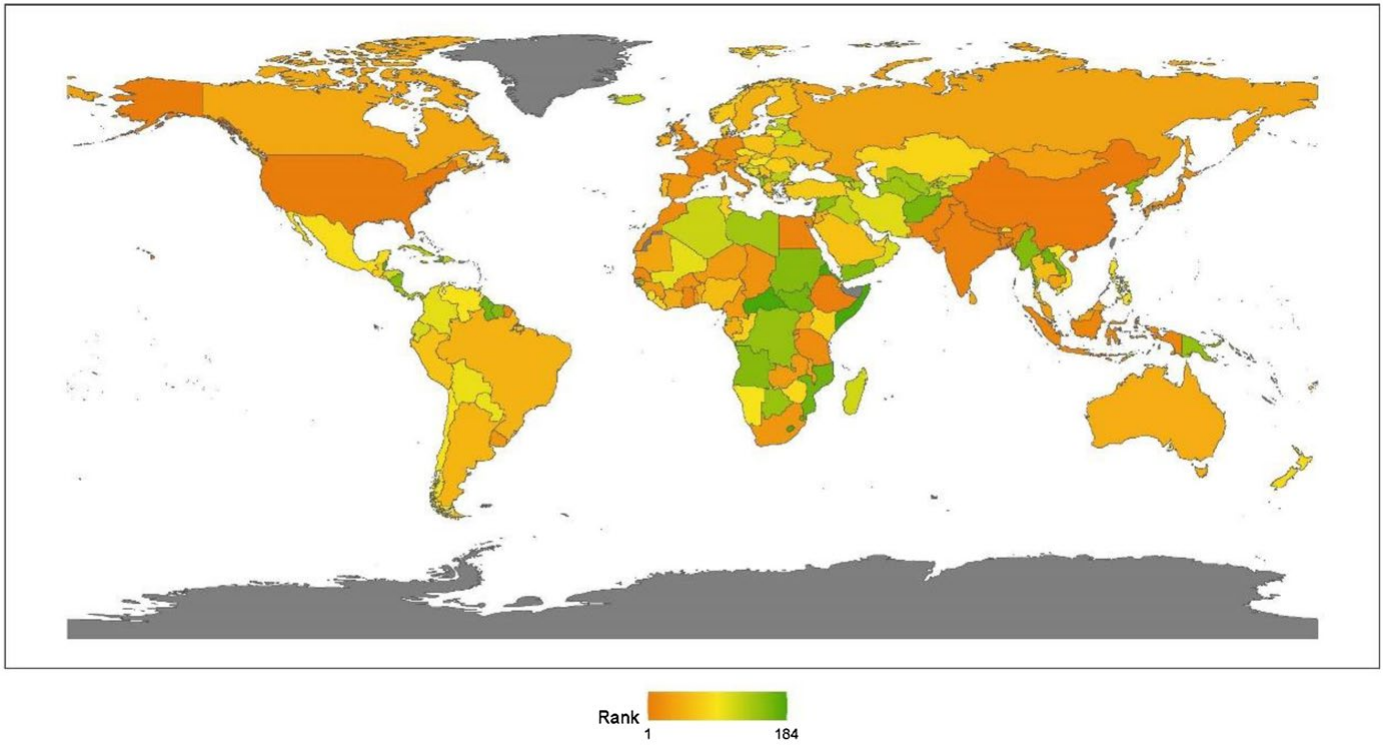
2020 index ranking of peacekeeping issues on a world map

Continents’ rankings are obtained by calculating the average ranking for the countries that pertain to that continent. The results for 2020 reveal little change from previous years. The geographic breakdown of regions with the ranking of peacekeeping aspects for promoting global justice, from the best to worst, is North America, Asia, Africa, Europe, Latin America, and Oceania, as shown in Fig. 2. This section offers an analytical review of the distribution of each continent’s performance in this issue.

Asia: Asia has performed well in.peacekeeping. The top-ranking countries are China, Bangladesh, Nepal, India, and Pakistan. A leader in peacekeeping, China has been involved in UN peacekeeping operations for more than 30 years and is a key force in UN peacekeeping. Whether in mission areas such as South Sudan, Mali, Lebanon, and Darfur, or in the face of the sudden onset of the COVID-19 pandemic, Chinese peacekeepers have committed to fulfilling their responsibilities, with 30,468 troops. In 2020, China’s contribution ratio to UN dues was 15.22%, making it the second-largest supporter to the UN peacekeeping budget. Nepal is the world’s fourth largest military contributor for UN peacekeeping operations, with about 68,062 Nepalese peacekeepers serving in 13 operations around the world.

The bottom-ranking countries in Asia are Yemen, the Maldives, Afghanistan, Lao People’s Democratic Republic, and Micronesia. None of these five countries sent peacekeepers in 2020, as they are underdeveloped nations with inadequate staffing and finances, and some of them are trapped in unstable political situations. Ending a 5-year civil war, Yemen experienced an unprecedented and uncontrolled COVID-19 pandemic, soaring food prices, and a sharp drop in foreign aid, thus undergoing an unprecedented humanitarian crisis. Terrorist attacks in Afghanistan continued to increase in 2020. The outbreak of COVID-19 could have a severe impact on Afghanistan’s peace progress.

Europe: Europe ranked fourth among the six continents in peacekeeping in 2020. It contributed 34.68% of UN peacekeeping funds, far ahead of the other continents. Despite the outstanding contribution from a financial perspective, Europe dispatched only 79,395 peacekeepers in 2020, much less than that of Asia and Africa.

The best-performing countries in Europe are France, Germany, Italy, the United Kingdom, and Spain. As with previous years, France’s results are remarkable, with an increase of 267 peacekeepers, mostly distributed in the United Nations Multidimensional Integrated Stabilization Mission in the Central African Republic and Mali. Spain also increased its peacekeeping forces in 2020, focusing on the United Nations Interim Force In Lebanon.

Norway’s ranking fell from fifty-eighth to sixty-fifth, mainly because the number of troops that it dispatched fell from 1228 in 2019 to 738 in 2020. Poland’s ranking went from eighty-first in 2019 to sixtieth in 2020. This praiseworthy improvement may be attributed to its troop contribution, with an increased number from 530 to 2708.

Montenegro, Liechtenstein, Albania, Andorra, and San Marino scored lower than the other countries in Europe. The main factor that dragged down their rankings was their very limited contributions to peacekeeping forces and financial support.

North America: North America ranked at the forefront generally in peacekeeping. The United States continued to take the second place in the field of peacekeeping in 2020. North America’s rate of financial contributions rate to UN peacekeeping was 30.64%, increasing its aggregated score.

As a permanent member of the UN Security Council and the largest funder of the Department of Peace Operations, the United States has an important leadership role to play in authorizing and shaping UN missions. The UN faces a cash crisis, with member states accumulating more than $1 billion in unpaid dues (about two-thirds of which is owed by the United States). Since 2017, the United States has accumulated over $900 million in total arrears, including approximately $776 million in the peacekeeping budget.

Canada ranked thirty-eighth in 2020, approximately the same as in 2019. Canada has supported the UN need to sustain its missions delivering resources to military and police personnel, as well as civilian staff on peacekeeping operations in multiple locations across the African continent. Like most countries, Canada withdrew a proportion of its peacekeepers in 2020. The number of Canadian peacekeepers in 2020 fell to 460, with a focus on Mali, South Sudan, and the Democratic Republic of the Congo.

Latin America: The ranking of Latin America was only slightly higher than that of Africa and Oceania and lower than the rankings of all other continents. The total contribution of Latin American peacekeepers increased from 28,957 in 2019 to 29,998 in 2020. Latin America’s financial contribution rate to UN peacekeeping was only 1.7%, far behind other continents. The number of peacekeepers safeguarding this continent was 1403, mainly distributed in Colombia and Haiti.

Uruguay, Brazil, Argentina, El Salvador, and Peru showed better scores than other Latin American countries. Uruguay’s global ranking rose from twenty-seventh in 2019 to twenty-fourth in 2020. The number of Uruguayan peacekeeping forces increased from 12,220 in 2019 to 13,647 in 2020, with a focus on missions in Golan and the Democratic Republic of the Congo. The global rankings of Brazil, Argentina, El Salvador, and Peru were relatively stable, in spite of a small withdrawal of peacekeeping forces.

The lowest scoring countries were Belize, Dominica, Grenada, Saint Lucia, and Saint Vincent and the Grenadines, similar to the result in 2019.

Africa: In the overall ranking, Africa is only lower than North America and Asia. In terms of peacekeeping personnel, African countries had the highest performance, with a cumulative contribution of 483,956 peacekeepers, consisting 48.9% of the total deployment. Conflicts and tensions persisted on this continent. The number of overall peacekeeping personnel protecting this continent was 835,575, far more than any other continent. More than half UN missions were conducted in Africa, such as in Western Sahara, Central African Republic, Mali, the Democratic Republic of the Congo, Guinea-Bissau, Sudan, Abyei, South Sudan, Libya, and Somalia. The United Nations Hybrid Operation in Darfur (UNAMID) and the UN Integrated Peacebuilding Office in Guinea-Bissau were closed in 2020. UNAMID’s closure is a landmark in contemporary peacekeeping.

The top five countries were Ethiopia, Rwanda, Egypt, Ghana, and Senegal. Ethiopia had a higher ranking than all other countries in Africa. With a population of more than 100 million, Ethiopia is an influential country in East Africa. Over the past 10 years, Ethiopia has achieved excellent performance in peacekeeping and is among the countries with the largest number of peacekeepers deployed. This makes its ranking and score firmly in first place on this continent. However, in 2020, Ethiopia was plunged into the vortex of a civil war, and it thinned out its peacekeeping forces as a consequence.

Although Rwanda began relatively late in the field of peacekeeping, it has rapidly developed and is now one of the countries that provides the most support for UN peacekeepers. In 2020, Rwanda dispatched 76,047 peacekeepers supporting United Nations Missions in South Sudan and Darfur and the UN Multidimensional Integrated Stabilization Mission in the Central African Republic, forming the third-largest contribution in terms of troops.

Mali fell from 93 to 103rd, halving the number of peacekeepers in 2020. Mali not only witnessed a political turmoil but also experienced more than seven terror attacks, in which at least 133 people were killed, including soldiers and civilians. At least six peacekeepers lost their lives and 24 were injured in the United Nations Multidimensional Integrated Stabilization Mission in Mali in 2020.

The ranking of Cote d’Ivoire has risen sharply, from the sixtieth rank in 2019 to the forty-second one in 2020. It sent 3453 troops in 2019 and increased this number to 7127 in 2020, in which 6555 Ivorian military and police personnel were deployed in the UN Mission in Mali.

The lowest ranking countries in Africa are Central African Republic, Comoros, Eritrea, Lesotho, Somalia, and Sao Tome and Principe. These six countries had the same low rank of 184th as in 2019, due to their limitations of national capabilities and population size. Central African Republic faced violent clashes in its north-east and north-west from April 2020 onwards. Armed conflict has again overshadowed the country’s peace process. Zambia, Rwanda, Russia, and France have also sent troops, military experts, and helicopter gunships to Central African Republic to make help stabilize the situation. Suffering a continuous civil war, Somalia remains mired in social unrest, and 2 of the 21 total UN peacekeeping operations occurred in Somalia.

Oceania: We included 13 Oceanian countries in our ranking. In 2020, Oceania contributed 4816 peacekeepers and 2.5% of the global peacekeeping funding.

Australia, Fiji, and New Zealand ranked higher than others in this region, at forty-third, fifty-second, and eighty-fourth. Australia sent 395 peacekeepers in 2020, focusing on the United Nations mission in South Sudan and the United Nations Truce Supervision Organization. The contribution in terms of troops for Fiji was 10.34 times that of Australia. Fiji dispatched 4083 peacekeepers in 2020, mainly supporting the United Nations Disengagement Observer Force for Golan and the United Nations Assistance Mission for Iraq.

The lowest ranking countries were the Solomon Islands, Tuvalu, and Vanuatu, unchanged from their ranking the previous year. Like the bottom-ranking African countries, they all ranked the 184th in 2020. The intra-continent differences in Oceania are greater than in other continents.

#### Conclusion

COVID-19 has had a profound influence on UN peacekeeping operations, forcing them to review, adjust, and restrict their activities. Within missions, COVID-19 has had several impacts. Some tasks have been suspended, either because they required close contact with local populations or because severely constrained missions have had to prioritize activities deemed more immediately critical. Meanwhile, the number of deaths due to illness among international and local personnel in UN peace operations in 2020 was almost double that in 2019. This difference is almost certainly linked to the pandemic and its impacts, which contributed to a record number of deaths across UN countries during the year.

The number of international personnel deployed in multilateral peace operations globally fell by 5.21%, falling from 1,043,555 in 2019 to 989,195 in 2020. The reasons for this were as follows. First, due to the financial difficulties of the United Nations and the necessity for fees to be paid, some major countries, such as Ethiopia, Rwanda, Nepal, and India, suffered serious losses, which weakened their enthusiasm to send troops in peacekeeping operations. Second, the COVID-19 pandemic forced countries to invest in controlling the spread of the epidemic and coping with the negative impacts, inevitably bringing about certain reductions in their financial ability to support UN peacekeeping. Third, a few countries’ domestic political turmoil and armed conflict shrank the scale of their peacekeeping contribution.

The driving factors influencing the contribution to UN peacekeeping could be listed as follows: domestic political stability, comprehensive national strength, sufficient financial sustainability, sufficient human capital, continuous peacekeeping history, and a desire to be influential and powerful in international affairs.

As a unique global partnership, UN peacekeeping brings together the General Assembly, the Security Council, the Secretariat, personnel contributors, and the host governments in a combined effort to maintain international peace and security and develop a community with a shared future for mankind. There is a large gap between countries with high-value financial and military capabilities to various United Nations peacekeeping missions and countries with insufficient resources and capabilities.

### Issue 3: Humanitarian Aid

#### Introduction

Humanitarian aid refers to assistance that focuses on providing basic needs, such as those of food, water, shelter, and medical care to those affected by disasters or other extreme circumstances. It can be given in the form of funding and supplies such as medicine, cookstoves, and blankets. It is typically provided by non-governmental organizations, governments, humanitarian organizations, and other specialized disaster relief entities. The purpose of such aid is to save lives, reduce suffering, and improve conditions for people in crisis.

Humanitarian aid promotes global justice by providing assistance to those in need, regardless of their race, nationality, or religion. Addressing the needs of those affected by natural disasters and other humanitarian crises without prejudice or bias helps protect their rights and dignity against violation. Additionally, humanitarian aid can help build bridges between communities of different backgrounds who would otherwise be unlikely to have contact with each other; this in turn fosters deeper understanding and more informed international relationships. We therefore included this issue in our global justice index and measured it using each country’s financial contribution to global humanitarian affairs.

#### Dimensions and Indicators

Last year, we assessed the humanitarian aid efforts of each country based on 11 distinct indicators. These were included food, health, housing, water, emergency response, early recovery, coordination, education, protection, agriculture, and others (the portion of the donation without an assigned use). The data for this analysis were collected from the UN’s Financial Tracking Service database, which records donations made by countries to other nations and to organizations such as UN departments and NGOs such as the World Food Program and WHO. We accounted for all types of donations, including those designated with a specific use-case and “other” contributions allocated without pre-specified purpose.

This year we changed our indicator system regarding humanitarian aid, in response to the worldwide COVID-19 pandemic. The pandemic caused a dramatic increase in the need for humanitarian aid, as many people have been adversely affected by disruptions in supply chains, economic losses, and access to health services. In addition, with travel restrictions preventing physical aid from being supplied to places in need as easily as before, efforts have been made to deliver a more virtual assistance through digital delivery methods such as online payments and text messaging campaigns. These technologies have allowed organizations to quickly reach those most at risk during this difficult period of history.

We are measuring each country’s humanitarian aid efforts in 2020 based on 12 indicators in all. Below, you will find detailed information about all of the metrics used to measure humanitarian aid (Table 5).

**Table 5:** Data on humanitarian aid

Category	Dimension	Indicator	Source	Coverage
Contribution	Humanitarian Donation	Food	Financial tracking service	186 countries (2020)
		Housing		
		Health		
		Water		
		Emergency response		
		Early recovery		
		Coordination		
		Education		
		Protection		
		Agriculture		
		COVID-19		
		Other		

To accurately gage the amounts of humanitarian donations, we combined the figures from all 11 indicators and took into account each country’s GDP per capita. This was done to ensure that countries with larger economies were not unduly favored over poorer nations in our assessment.

#### Results

This section reports the ranking results of the countries’ contribution to global justice from the perspective of humanitarian aid. Please see the detailed rankings in Table 6.

**Table 6:** Country ranking in humanitarian aid

Country	Ranking	Country	Ranking
United States of America	1	Georgia	83
Somalia	2	Costa Rica	83
Germany	3	Eswatini	83
United Kingdom of Great Britain and Northern Ireland	4	Iraq	83
Haiti	5	Bolivia (Plurinational State of)	83
Saudi Arabia	6	Zimbabwe	83
Chad	7	Micronesia (Federated States of)	83
Japan	8	Grenada	83
Bangladesh	9	El Salvador	83
Sweden	10	Libya	83
Canada	11	Jamaica	83
Norway	12	Tonga	83
United Arab Emirates	13	Angola	83
Burundi	14	Papua New Guinea	83
Mozambique	15	Uruguay	83
Netherlands	16	Saint Vincent and the Grenadines	83
Italy	17	Mali	83
Russian Federation	18	Namibia	83
France	19	Equatorial Guinea	83
Kuwait	20	Yemen	83
Switzerland	21	Uzbekistan	83
Denmark	22	Ecuador	83
Australia	23	Honduras	83
Belgium	24	Sao Tome and Principe	83
China	25	Bahrain	83
Republic of Korea	26	Barbados	83
Finland	27	Guinea-Bissau	83
Colombia	28	Dominica	83
Azerbaijan	29	Nepal	83
Spain	30	Sierra Leone	83
Cameroon	31	Rwanda	83
Ireland	32	Djibouti	83
Qatar	33	Paraguay	83
New Zealand	34	Algeria	83
Turkey	35	Bosnia and Herzegovina	83
Austria	36	Sudan	83
Pakistan	37	Palau	83
Kazakhstan	38	United Republic of Tanzania	83
Nigeria	39	Central African Republic	83
Luxembourg	40	Cabo Verde	83
Poland	41	Congo	83
Cote d’Ivoire	42	Senegal	83
Portugal	43	Zambia	83
Serbia	44	Liberia	83
Czechia	45	Kenya	83
Estonia	46	Republic of Moldova	83
Iceland	47	Samoa	83
Belarus	48	Nicaragua	83
Gabon	49	Jordan	83
Brazil	50	Benin	83
Croatia	51	Tuvalu	83
Indonesia	52	Guatemala	83
Bulgaria	53	Albania	83
Slovenia	54	Argentina	83
Lithuania	54	Seychelles	83
Romania	55	Afghanistan	83
Slovakia	56	Uganda	83
Malta	57	Cuba	83
Viet Nam	58	Suriname	83
Morocco	59	Ghana	83
Thailand	60	Niger	83
Philippines	61	Solomon Islands	83
South Africa	62	Kiribati	83
Oman	63	Botswana	83
Cambodia	64	Brunei Darussalam	83
Monaco	65	Israel	83
Malaysia	66	Vanuatu	83
Singapore	67	Antigua and Barbuda	83
Cyprus	68	Mauritius	83
Myanmar	69	Republic of North Macedonia	83
Hungary	70	Bahamas	83
Latvia	71	Marshall Islands	83
Greece	72	Trinidad and Tobago	83
Iran (Islamic Republic of)	73	India	83
Belize	74	Ethiopia	83
Sri Lanka	75	Saint Lucia	83
Mongolia	76	Lebanon	83
Andorra	77	Lesotho	83
Timor-Leste	78	Fiji	83
Armenia	79	San Marino	83
Bhutan	80	Togo	83
Peru	81	Nauru	83
Guyana	82	Egypt	83
Montenegro	82	Maldives	83
Madagascar	83	Saint Kitts and Nevis	83
Chile	83	Mauritania	83
Ukraine	83	Malawi	83
Democratic Republic of the Congo	83	Mexico	83
Comoros	83	Guinea	83
Burkina Faso	83	Tajikistan	83
Dominican Republic	83	Tunisia	83
Gambia	83	Lao People’s Democratic Republic	83
Panama	83	Kyrgyzstan	83

The United States retains the highest ranking on the issue of humanitarian aid. The top 10 countries on this issue are the United States of America, Somalia, Germany, the United Kingdom, Haiti, Saudi Arabia, Chad, Japan, Bangladesh, and Sweden.

#### Regional Analysis

**Fig. 3:**
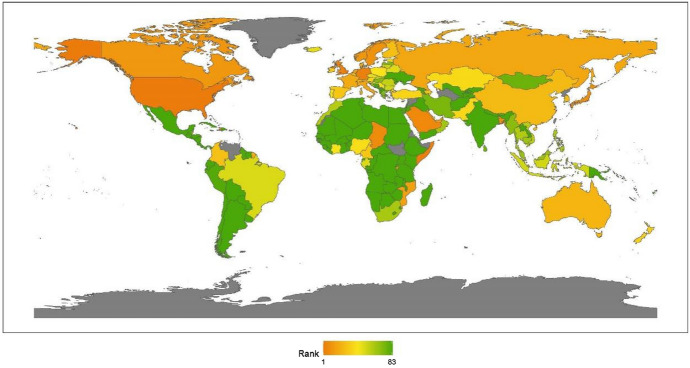
2020 index ranking of humanitarian aid on a world map

This section reports the regional analysis of the ranking of humanitarian aid. Figure 3 shows country rankings on a world map.

Asia: Saudi Arabia remained the highest ranking Asian country on this issue, followed by Japan and the United Arab Emirates. Saudi Arabia was one of the largest donors in 2020. Its contribution was multifaceted and showed a commitment to providing assistance on multiple levels. This included food, shelter, medical care, and other help for those affected by conflicts or natural disasters. It also implemented various programs to protect vulnerable people, such as victims of abuse or human trafficking. Furthermore, Saudi Arabia conducted projects that sought to empower individuals, including those living in poverty, by giving them access to skills training and job opportunities.

Japan was also among the top donors for humanitarian aid in 2020, contributing over $1.2 billion dollars to those affected by war, conflict, and disaster. This included support for providing food and shelter as well as medical services. Additionally, Japan also provided funding toward projects seeking to protect members of vulnerable groups, such as refugees or victims of human trafficking and child labor exploitation. These efforts demonstrate Japan’s commitment to global justice initiatives, as well making a positive impact around the world through generous contributions aimed at helping people in need.

Europe: Europe continued its high level of performance in humanitarian aid in 2020. Germany was the top European country on this issue in 2020, followed by the UK and Sweden. Germany contributed over $3 billion dollars to those affected by war and disasters.

European countries worked together to promote humanitarian aid in 2020 through a range of initiatives. These include providing financial and material support for those affected by conflicts or natural disasters, as well as developing a long-term strategy to reduce poverty, promote human rights, and create a more secure environment for vulnerable groups. Additionally, Europe has also created joint policy proposals such as the Paris Declaration on Aid Effectiveness, which seeks greater coordination between nations in international development assistance projects. Other legislation includes the creation of instruments that provide cross-border cooperation between EU member states for disaster relief operations or to facilitate the access of medical personnel into areas affected by conflict zones or other emergencies.

In addition, European countries have cooperated to provide humanitarian aid in response to the COVID-19 pandemic. These efforts include providing financial and material assistance and developing strategies for distributing critical medical supplies, including ventilators and personal protective equipment. Additionally, initiatives such as the EU Emergency Support Instrument have been launched to fast-track emergency funding for projects being conducted to alleviate socio-economic challenges faced by those affected by the virus. Europe has also implemented specific emergency measures such as commissioning a Coronavirus Global Response Platform, which seeks to raise additional funds from global partners that can be used for research on treatments and vaccines worldwide.

North America: The United States and Canada performed well on this issue in 2020. The United States contributed over $9 billion dollars to aid sectors including food security, nutrition, and health. Additionally, the US took part in initiatives such as the World Food Programme’s COVID-19 Response Plan, which seeks to help prevent hunger that results from disruptions leading from the pandemic through direct cash transfers or vouchers for nutritious meals provided online via an app. In response to the COVID-19 pandemic, the US provided over $5 billion in emergency aid both at home and abroad. This includes funding for the World Health Organization, distributing protective gear and medical supplies, providing funding for cash assistance programs, supporting vaccine programs, and expanding access to healthcare.

Canada provided over $600 million in humanitarian aid in 2020, including funding provided to UN agencies, directly to affected countries, or through Canadian NGOs. This includes funding to address global issues such as the COVID-19 pandemic, climate change, food insecurity, and refugees. For example, in 2020, Canada provided $78 million to the UN Refugee Agency, $63 million to COVID-19 Response Fund, and $200 million for Global Climate Action. It also launched a $150 million Food Security and Nutrition fund, an $18 million International Humanitarian Assistance program, and $50 million Humanitarian Assistance to address urgent needs caused by conflict and natural disasters.

Latin America: Haiti was the only Latin American country in the top 10 on this issue in 2020. Haiti has a population of approximately 11 million people and is classified as a low-income country by the World Bank. Its economy is primarily based on agriculture and services, and its GDP per capita was estimated at $781 USD in 2019. Haiti is among the most-vulnerable countries in the world to climate change and is prone to natural disasters, including hurricanes and floods. However, it contributed over $60 million in humanitarian aid in 2020. As noted, we took GDP per capita into consideration in our calculation to ensure that countries with larger economies were not unduly favored over poorer nations. This perspective, allowed Haiti’s 2020 contribution in light of its relatively weak economy to bring it to a top 10 position in humanitarian aid.

Africa: There were several African countries with weak economies that performed very well in 2020 and had a high ranking. Among the top 10 countries, Somalia, and Chad are African countries. Burundi and Mozambique contributed significantly as well with ranks of 14 and 15.

Somalia is also classified as a low-income country by the World Bank. Its economy is primarily based on agriculture and services and it is the second-poorest country in the world. It suffers from political instability, conflict, natural disasters, malnutrition, and a lack of access to basic services such as healthcare and education. It contributed more than $50 million dollars in humanitarian aid in 2020.

In absolute terms, Nigeria, South Africa, and Kenya provided the most aid. This aid was directed toward responding to the COVID-19 pandemic and addressing the urgent needs caused by conflict and natural disasters. African governments and organizations are also investing in initiatives to enhance local resilience and build the capacity of vulnerable communities.

According to the United Nations, Africa was the largest recipient of humanitarian aid in 2020, receiving over $3.9 billion dollars. This includes assistance in response to the COVID-19 pandemic as well as for addressing other urgent needs caused by conflict, natural disasters, and food insecurity.

Oceania: A large gap was seen with respect to the contribution made by Australia and New Zealand and that by other countries in Oceania. Australia ranked twenty-third in the all-country ranking, and New Zealand ranked the thirty-fourth. These are the only two countries who provided humanitarian assistance in Oceania.

Australia provided over $264 million dollars in humanitarian aid in 2020. In response to the COVID-19 pandemic, Australia provided over $400 million in aid, including funds for the World Health Organization, providing protective gear and medical supplies, as well as funding for cash assistance programs. It also provided over $200 million in humanitarian assistance, including $30 million to help vulnerable people in Pacific Island countries and the Asia-Pacific region and $75 million to support the United Nations Commission on Human Rights in conflict and crises.

#### Conclusion

Humanitarian aid has long played an important part in global justice because it helps alleviate the suffering of vulnerable populations, address disparities and inequalities, and promote the human rights of individuals and communities. It provides a lifeline of assistance in times crises and can help prevent the further erosion of human rights, including the right to health, food, water, education, and a dignified standard of living.

In light of the COVID-19 pandemic, we revised our indicator system and began to measure the contribution of each country through 12 indicators in all: food, health, housing, water, emergency response, early recovery, coordination, education, COVID-19, protection, agriculture, and others. After this adjustment, the United States retains the top ranking on the issue of humanitarian aid. The top 10 countries on this issue are the United States of America, Somalia, Germany, the United Kingdom of Great Britain and Northern Ireland, Haiti, Saudi Arabia, Chad, Japan, Bangladesh, and Sweden. This aid helped to provide emergency food assistance and nutrition support, strengthen disaster risk management capabilities and improve access to healthcare and education services on a global scale.

### Issue 4: Anti-terrorism and Conflicts

#### Introduction

Terrorism, violent extremism, conflict, and war pose significant challenges to global security and stability. Although though the world is generally safer now than it has been in the past, these threats continue to affect societies worldwide, both directly and indirectly. For example, in 2020, over 300,000 individuals lost their lives due to terrorist attacks, armed conflict, and war.^29^^,^ 30 In addition to the direct destruction of property and lives, terrorism, conflict, and war also indirectly affect the world by creating market uncertainty, introducing fear, increasing xenophobia, and causing loss of tourism.^31^ All of these impacts eventually undermine the process of global justice.

In March 2020, UN Secretary-General António Guterres called for global ceasefire in all corners of the world to fight the COVID-19 pandemic, recognizing it as a crisis of solidarity, cooperation, and diplomacy.^32^ Despite this, conflicts continue, although some warring parties have temporarily suspended hostilities. There is no doubt that COVID-19 has had a mixed impact worldwide. However, it is difficult to generalize global trends in conflict and violence, as the situation is complex and constantly evolving. For example, in some regions, due to lockdowns and travel restrictions, which reduced mobility and gatherings, the frequency and intensity of terrorist attacks and armed conflict were temporally reduced. However, a shift toward smaller scale, low-sophistication attacks or online propaganda and recruitment efforts by terrorists have appeared.^33^ In addition, increased poverty and unemployment can be drivers of terrorism and conflict, particularly in regions that are already fragile or that are facing pre-existing security challenges.^34^

In this study, we evaluate and compare the performance of different countries in their contribution to anti-terrorism and conflict. By analyzing each country’s data, we can better understand the current state of conflict and terrorism worldwide and the global effort to combat them. Finally, we can provide a perspective for describing global justice.

#### Dimensions and Indicators

As in the 2019 report, 192 countries were involved in the 2020 report regarding anti-terrorism and conflicts. We consider three dimensions to quantify the performance and contribution in addressing terrorism and conflicts of each country. Each dimension is represented by two or three observed indicators. Table 7 shows the data framework and sources.

**Table 7:** Data on anti-terrorism and armed conflicts

Category	Dimension	Indicator	Data source	Coverage
Performance	Conflicts	Number of conflicts	UCDP Armed Conflict Dataset	192 countries
		Number of wars		
		Number of conflict deaths		
Contribution	Conflict Agreements	Number of agreements		
		Achievements of agreements		
Performance	Terrorism	Number of terrorism attacks	Global Terrorism Dataset	
		Number of deaths from terrorism attacks		

Conflicts and terrorism dimensions are measured by the number of acts and the number of deaths that resulted. The indicators are negatively related to the index score. The larger the value, the worse the country’s performance in combating terrorism and conflict. All of these indicators are weighted by population size to ensure that each country is represented proportionally to its population. Conflict, including war (i.e., the extreme form of the conflict), claims more deaths than terrorism globally. In 2020, the total number of deaths due to conflicts and terrorism was around 296,000^35^ and 23,000,^36^ respectively.

The conflict agreements dimension reflects the contribution of each country to terminating conflicts. We measured this dimension by the number of agreements achieved. Every signed peace agreement is a result of long-term efforts among the involved parties. The achievement of agreements is a function of eight related indicators, chosen from the Uppsala Conflict Data Program (UCDP) Armed Conflict Dataset. As was done for previous reports, we designed a function to compute the achievement score for each agreement and then adopted an integral retrospective method to assign the score for the prior four years. As mentioned in last year’s report, the peace agreement data were unavailable during the data-collecting period; thus, the data for this dimension were imputed from 2018 data. This year’s report updated the agreement data for both 2019 and 2020, collected by the UCDP Armed Conflict Dataset. In 2019, six agreements were signed, and in 2020, eight were finalized.

#### Results

In this section, we present a sub-index ranking of 192 countries in 2020 based on their performance and contribution to global justice from the anti-terrorism and conflicts aspect. Table 8 displays the sub-index result.

**Table 8:** Country rankings in the anti-terrorism and conflict aspects of promoting global justice in 2020

Country	Ranking	Country	Ranking
Sudan	1	United States of America	97
India	2	Sweden	98
Myanmar	3	Timor-Leste	99
South Sudan	4	Tunisia	100
Japan	5	Mauritius	101
China	6	Togo	102
Vietnam	7	Switzerland	103
Republic of Korea	8	Eswatini	104
Brazil	9	Ghana	105
Uzbekistan	10	Lebanon	106
Democratic People's Republic of Korea	11	Austria	107
Morocco	12	El Salvador	108
Poland	13	Trinidad and Tobago	109
Malaysia	14	Kenya	110
Indonesia	15	New Zealand	111
Argentina	16	Denmark	112
Peru	17	Fiji	113
Algeria	18	Chile	114
Cuba	19	Netherlands	115
Angola	20	Comoros	116
Dominican Republic	21	Finland	117
Mexico	22	Saudi Arabia	118
Zimbabwe	23	Liberia	119
Hungary	24	Zambia	120
Tajikistan	25	Norway	121
Bangladesh	26	Turkey	122
Papua New Guinea	27	Philippines	123
Kazakhstan	28	Mauritania	124
Lao People’s Democratic Republic	29	Solomon Islands	125
Spain	30	Luxembourg	126
Serbia	31	Montenegro	127
Kyrgyzstan	32	Suriname	128
Ecuador	33	Colombia	129
Turkmenistan	34	Cabo Verde	130
Haiti	35	Greece	131
Singapore	36	Bosnia and Herzegovina	132
Germany	37	Malta	133
Slovakia	38	Ireland	134
Belarus	39	Ethiopia	135
Italy	40	United Arab Emirates	136
Venezuela (Bolivarian Republic of)	41	Libya	137
Oman	42	Lithuania	138
Costa Rica	43	Brunei Darussalam	139
Bulgaria	44	Nigeria	140
Honduras	45	Belize	141
Panama	46	Burundi	142
Kuwait	47	Bahamas	143
Rwanda	48	Iceland	144
Madagascar	49	Gambia	145
Thailand	50	Sierra Leone	146
Cote d’Ivoire	51	Maldives	147
United Republic of Tanzania	52	Vanuatu	148
Paraguay	53	Latvia	149
South Africa	54	Israel	150
Congo	55	Democratic Republic of the Congo	151
Uruguay	56	Barbados	152
Mongolia	57	Niger	153
Canada	58	Chad	154
Russian Federation	59	Cameroon	155
Sri Lanka	60	Guyana	156
Australia	61	Cyprus	157
Jamaica	62	Sao Tome and Principe	158
Nicaragua	63	Estonia	159
Qatar	64	Eritrea	160
Romania	65	Samoa	161
Georgia	66	Mozambique	162
Bolivia (Plurinational State of)	67	Central African Republic	163
Croatia	68	Azerbaijan	164
Republic of Moldova	69	Mali	165
Namibia	70	Burkina Faso	166
Senegal	71	Saint Lucia	167
Cambodia	72	Bhutan	168
Botswana	73	Kiribati	169
Guatemala	74	Micronesia (Federated States of)	170
Egypt	75	Grenada	171
Malawi	76	Saint Vincent and the Grenadines	172
Lesotho	77	Iraq	173
Uganda	78	Tonga	174
France	79	Seychelles	175
Slovenia	80	Antigua and Barbuda	176
Albania	81	Yemen	177
Republic of North Macedonia	82	Bahrain	178
Ukraine	83	Andorra	179
Guinea-Bissau	84	Syrian Arab Republic	180
Guinea	85	Somalia	181
Czechia	86	Marshall Islands	182
Benin	87	Djibouti	183
Iran (Islamic Republic of)	88	Saint Kitts and Nevis	184
Belgium	89	Dominica	185
Jordan	90	Armenia	186
Gabon	91	Monaco	187
United Kingdom of Great Britain and Northern Ireland	92	San Marino	188
Portugal	93	Palau	189
Nepal	94	Afghanistan	190
Equatorial Guinea	95	Tuvalu	191
Pakistan	96	Nauru	192

Most countries’ rankings are consistent with previous rankings. Where there are large shifts in ranking, the signing of peace agreements is typically the reason. All of the top four countries, Sudan, India, Myanmar, and South Sudan, signed one or more peace accords in 2020. With the exception of South Sudan, which ranked in the top tier in 2019, the rankings of the other three countries have improved significantly. Unlike the other better performing countries, the top four had relatively lower ranks on the anti-conflict and/or anti-terrorism dimensions. The reason for emphasizing the peace agreement dimension on this issue is that we believe that any conflict has an extremely negative impact on global justice, and the effort to make an agreement to end conflict within or between the countries should be recognized and appreciated.^37^

Sudan ranked first in 2020, mainly due to its remarkable contribution to the conflict agreement dimension. In 2020, the transitional government of Sudan signed partial peace agreements with several rebel groups. On October 3, 2020, the government and a coalition of several of the largest armed groups signed the Juba Peace Agreement,^38^ which sought to end the country’s decades-long armed conflict, in which thousands of people had been killed, and millions had been displaced. However, this agreement did not bring permanent peace to Sudan, as conflicts and terrorist attacks continued. Although it had the top rank in the agreement dimension, Sudan ranked 133rd in the anti-terrorism dimension and 110th in the anti-conflict dimension.

India ranked second on this issue, performing well on the anti-conflict and agreement dimensions. For anti-conflict, India had the highest score, with no war in 2020. However, it nevertheless had two conflicts and 187 conflict deaths.^39^ On January 27, 2020, the Indian government signed an agreement with the National Democratic Front of Borland, hoping to provide political and economic opportunities and boost peace in the northeast.^40^ This is the third accord attempting to end the Bodo militancy in as many decades, and it is expected to bring lasting peace to the state. In 2020, India suffered 450 terrorist attacks, in which 212 people lost their lives.

Myanmar ranked third. On August 21, 2020, in Nay Pyi Taw, the Myanmar government and four political and military organizations signed the Union Accord Part III, which was considered to be a necessary precursor to forming a Democratic Federal Union. This historic peace agreement includes three parts: the implementation of the Nationwide Ceasefire Agreement, post-election activities, and fundamental federal principles.^41^ With the exception of the outstanding performance in the conflict agreement dimension, Myanmar performed well on the anti-conflict dimension, witnessing no conflict or war. However, on the anti-terrorism dimension, Myanmar ranked 122nd, with 65 terrorist attacks and 41 deaths.^42^

South Sudan ranked fourth. In January 2020, the government of South Sudan and the armed parties signed a peace declaration in Rome, committing the parties to ceasefire and continuing peace talks.^43^ South Sudan ranked 34th and 146th on anti-conflict and anti-terrorism, respectively. It had no conflict or war took place, but 16 terrorist attacks claimed 56 deaths. The country continues to experience instability, economic stagnation, and fragility more than a decade after its independence in 2011. Conflict and other external shocks compounded the widespread problem of extreme poverty.^44^

The other top-ranking countries that made consistently remarkable contributions to fighting terrorism and conflict in 2020 were Japan, China, Vietnam, the Republic of Korea, Brazil, and Uzbekistan. With the exception of Sudan, South Sudan, and Brazil, all of the countries in the top 10 are all in Asia from the top list. Nauru, Tuvalu, Afghanistan, Palau, and San Marino occupied the bottom five spots this year and have been at the bottom for a long time. With the exception of Afghanistan, the other lowest ranking countries had no conflict or terrorist attack; their poor results are simply due to the population-weighted analysis model.

Afghanistan is one of the most dangerous countries in the world. It ranked 190th in this issue overall and 172nd and 192nd on the anti-conflict and anti-terrorism dimensions. Two conflicts and one war claimed 40,524 lives,^45^ and more than 10,000 people died in terrorist attacks, which accounted for 44% of all terrorism-related deaths worldwide.^46^ Afghanistan had the highest impact of terrorism in 2020, which is its second time ranking first on the Global Terrorism Index.^47^ The Taliban was the most active terrorist group in Afghanistan, followed by the Khorasan Chapter of Islamic State, Khorasan Province, and the Islamic State (IS). After long negotiations, the Taliban and the United States signed the Agreement for Bringing Peace to Afghanistan, commonly known as the US–Taliban deal, on February 29, 2020.^48^ This deal was intended to bring an end to the war in Afghanistan that began in 2001.

#### Regional Analysis

**Fig. 4:**
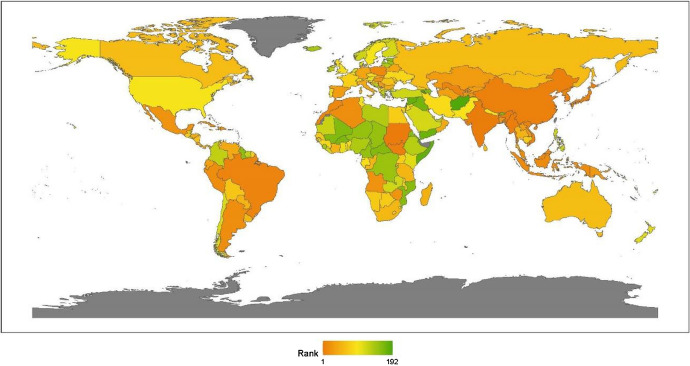
2020 index ranking of anti-terrorism and conflicts

Regional analysis enables us to investigate a specific region or sub-region to better understand local attributes and trends. In Fig. 4, we present a visualization of the global ranking. From a continental perspective, the region ranking of anti-terrorism and conflicts from high to low is Africa, North America, Europe, Latin America, Asia, and Oceania.

Asia: Asia ranked better than Oceania in 2020 but lower than every other continent. Anti-terrorism and conflict efforts in Asia varied widely across the region. Some countries made progress in reducing the threat of terrorism and conflict, while others faced significant challenges in addressing these issues. South-Eastern, Eastern, and Central Asia were in the top tier globally regarding anti-terrorism and conflicts. Western Asia (especially the Middle East) and South-Central Asia had the most conflicts and terrorist attacks.

War, as the extreme form of conflict, is more intense and widespread. It usually leads to more deaths and causes broader and longer lasting damage than conflict. Therefore, war is detrimental to global justice. In 2020, 13 out of 34 wars in the world happened in Asia, especially in the Middle East and Afghanistan, where over 65,000 people died.^49^ The top countries with the highest number of terrorist attacks in 2020 were Afghanistan, Iraq, and Yemen, all located in this region. The intense attacks are primarily due to political unrest and instability, which terror groups, particularly Taliban, IS, and AI-Qaeda, use to their advantage.^50^

Four Asian countries, Tajikistan, Sri Lanka, Nepal, and Thailand, significantly improved their ranking in this area from last year. These countries made outstanding contributions to the fight against terrorism. As a result, the number of attacks and deaths dropped significantly in 2020.

Europe: In 2020, Europe ranked third in this issue. It ranked fourth, third, and second in three dimensions, respectively. Ranking from high to low conflict, the sub-regions were Eastern, Northern, Southern, and Western Europe.

The global rankings were relatively stable for all the Europe countries except for Russia and the Netherlands. Russia’s ranking improved by 26 places, moving from eighty-fifth in 2019 to fifty-ninth in 2020. Deaths from terrorism in Russia fell to six deaths in three attacks in 2020. The deaths from conflict fell to 4652, representing a 37% decrease from the prior year. Deaths from war also decreased from 6865 to 4589, respectively, a decrease of 33%.^51^ However, the ranking of the Netherlands fell from eighty-third in 2019 to 115th in 2020. Despite the fall in deaths, the number of terrorist attacks rose from 4 in 2019 to 37 in 2020, the highest number of over in the past decade. The main threats to the Netherlands are Islamist terrorism and REMVE.^52^ After the Netherlands, the UK, Ukraine, Greece, Germany, and France suffered the most terrorist attacks in Europe in 2020.

North America: North America ranked second in the overall ranking. It had the top rank on both the anti-conflict and anti-terrorism dimensions and was third on the conflict agreement dimension. Within the region, the United States ranked ninety-seventh and Canada ranked fifty-eighth in this issue.

The US’s rank improved with the declining numbers of all kinds of conflicts and terrorism activities in 2020. The total of 12 deaths in the US due to terrorism was the lowest annual number recorded there after 9/11. Canada, on the contrary, fell to fifty-eighth in 2020 from fifty-third in 2019 due to an increase in terrorist activity. It recorded 14 attacks in 2020, an increase of 11 over 2019. Despite the increased number of attacks, only three deaths were recorded due to terrorism in 2020.

Latin America: Latin America ranked fourth globally. In each of its three dimensions, it likewise occupied the middle position. The sub-regions from top to bottom goes from South America to Central America and to the Caribbean. South American countries, Brazil, Argentina, Peru, and the Caribbean countries, Cuba, Dominican Republic, were ranked in the top five in this region and also had top rankings globally. It is worth noting that the low-ranking countries in the Caribbean did not encounter more conflicts or terrorist attacks; the lower rankings were mainly due to their tiny populations, as the anti-conflict, and counter-terrorism dimensions are population-weighted.

From a data perspective, Mexico, Guatemala, El Salvador, and Colombia suffered relatively serious conflicts or terrorist attacks. Specifically, in 2020, Colombia had 171 terrorist attacks on record, the highest among Latin American countries. Rebel groups continued to carry out several bombings and make other attacks, targeting infrastructure and civilians. In addition, criminal gangs and drug trafficking organizations carried out violent incidents, including bombings and assassinations. The other three countries had more conflicts and conflict deaths. They faced significant levels of conflict and violence, driven by a variety of factors, including gang activity, drug trafficking, and poverty.

Africa: Africa ranked the top one on this issue. On the dimension level, it ranked second, first, and third on anti-conflict, conflict agreement, and anti-terrorism dimensions, respectively. In the sub-regional results, the overall ranking from high to low was Northern, Southern, Middle, Western, and Eastern Africa. Even with its high rankings on the anti-conflict and counter-terrorism, Africa is not a peaceful place. Overall, in 2020, Africa accounted for 47% of instances and about 30% of deaths from conflict, 25% of instances and 40% of deaths from terrorism, and about half of the times and 25% of deaths from war globally.

The Sub-Saharan Africa, especially the Sahel region, is of serious concern, as the growth of Islamic State (IS) associates has resulted in a spike in terrorism across several countries in the region.^53^ Moreover, poverty, climate change, food insecurity, and political instability have led to more conflicts and terrorist attacks in the Sahel.^54^ In 2020, Nigeria had six conflicts and 4513 corresponding deaths, the most in Africa. It continued to face several security challenges, including ongoing conflict with Boko Haram and other militant groups in the northeast and communal and ethnic violence in other parts of the country.

Oceania: As regards the anti-terrorism and conflict issue, Oceania had a worse average score than elsewhere in 2020. With the exception for Papua New Guinea, the top-ranking country ranked twenty-seventh globally, and all the other countries ranked below sixtieth. When delving into the data for the indicators, we found that only Australia and New Zealand had conflicts and/or terrorist attacks on record; the other countries did not have any related activities or deaths. Australia encountered two conflicts and five terrorist attacks, with 580 and 2 deaths, respectively, in 2020. New Zealand had 12 terrorist attacks and no deaths. The geographic isolation of the island countries in Oceania has primarily shielded them from major terrorist events. As with the small population countries in the Caribbean area, the tiny island countries of Oceania owe their low rankings to an analysis model, not to their poor performance regarding anti-terrorism and conflict.

#### Conclusion

In this study, we collected data from the UCDP Armed Conflict Dataset and the Global Terrorism Dataset. Then, we used a population-weighted model to calculate each country’s score on anti-conflict, conflict agreement, and anti-terrorism dimensions in relation to global justice. The results indicated that Africa had the best performance in 2020, while Oceania had the worst. It was also found that countries with signed peace agreements had higher rankings on this issue.

Conflicts and terrorist activity are highly correlated. For example, places with the most terrorist attacks and conflicts commonly include sites of political instability, ethnic and religious tension, poverty, and resource competition. Nearly 80% of all terrorist acts take place within 50 km of a conflict, and 97% of terrorist fatalities happen in conflict zones.^55^ In addition, these countries usually have a long history of conflict, with many ongoing disputes and a history of foreign intervention. The presence of non-state armed groups, including terrorist organizations, also contributes to the high level of violence. In 2020, the sub-regions with the most terrorism deaths were Sahel, Southern Asia, and Middle East and Northern Africa.^56^ Of the total, 90 countries had at least one conflict and 27 experienced war.

One thing worth noting is that from the global data perspective, it seems that the pandemic has had very little direct impact on terrorism in 2020. However, long-term effects remain uncertain and may vary by the region.

### Issue 5: Cross-National Criminal Police Cooperation

#### Introduction

Cross-national criminal police cooperation is important for global justice because it allows law enforcement agencies to work together more effectively to identify, prevent, and respond to transnational crime. Transnational crime is any crime that occurs across multiple countries. It may involve sophisticated networks of individuals and organizations that operate on a global scale to facilitate illegal activities. The main characteristics of transnational crime include its international scope, complexity, profitability, scalability, and the anonymity associated with the crimes. By exchanging information and resources through cross-national criminal police cooperation, law enforcement can better identify and apprehend suspects, reduce the risk of international crime, and keep citizens safe. As a result, we incorporated this issue into our global justice index and evaluated each country’s contributions to combating transnational crimes.

The outbreak of the COVID-19 pandemic has had an impact on cross-national criminal police cooperation. Many countries have implemented travel bans and other restrictions that have severely impeded the ability of law enforcement to cooperate across national borders. In addition, as remote working becomes increasingly common due to the pandemic, online collaboration between law enforcement agencies has become even more important for them to tackle transnational crime effectively.

#### Dimensions and Indicators

This year, we have adopted the same evaluation system with 14 indicators to maintain consistency in our assessment. We measure the ratification status of each country and its performance in accordance with a set of UN treaties intended to reduce and combat transnational organized crime. These treaties include general treaties against transnational organized crime (United Nations Convention against Transnational Organized Crime, Protocol to Prevent, Suppress and Punish Trafficking in Persons, Especially Women and Children, supplementing the United Nations Convention against Transnational Organized Crime, Protocol against the Smuggling of Migrants by Land, Sea and Air, supplementing the United Nations Convention against Transnational Organized Crime, Protocol against the Illicit Manufacturing of and Trafficking in Firearms, Their Parts and Components and Ammunition, supplementing the United Nations Convention against Transnational Organized Crime), treaties against drugs and psychotropic substances (Single Convention on Narcotic Drugs of 1961 as amended by the 1972 Protocol, Convention on Psychotropic Substances of 1971, United Nations Convention against Illicit Traffic in Narcotic Drugs and Psychotropic Substances of 1988), a treaty against corruption (United Nations Convention against Corruption), and a treaty against taking of hostages (International Convention Against the Taking of Hostages). For the category of contribution, we measure their donation to Interpol, their donation to UNODC, and their FATF membership.

Please see below the details of all the indicators in the measurement of global cooperation against transnational crime (Table 9).

**Table 9:** Data on cross-national criminal police cooperation

Category	Dimension	Indicator	Data source	Coverage
Performance	General	United Nations Convention against Transnational Organized Crime	UN treaties	186 countries (2020)
		Protocol to Prevent, Suppress, and Punish Trafficking in Persons, Especially Women and Children, supplementing the United Nations Convention against Transnational Organized Crime		
		Protocol against the Smuggling of Migrants by Land, Sea, and Air, supplementing the United Nations Convention against Transnational Organized Crime		
		Protocol against the Illicit Manufacturing of and Trafficking in Firearms, Their Parts, and Components and Ammunition, supplementing the United Nations Convention against Transnational Organized Crime		
	Drugs and Psychotropic Substances	Single Convention on Narcotic Drugs of 1961, as amended by the 1972 Protocol	UN treaties	186 countries (2020)
		Convention on Psychotropic Substances of 1971		
		United Nations Convention against Illicit Traffic in Narcotic Drugs and Psychotropic Substances of 1988		
	Corruption	United Nations Convention against Corruption		
	Taking of Hostages	International Convention Against the Taking of Hostages 1979.12.17		
Contribution	Donation to Interpol	Donation to Interpol/GDP per capita	Interpol	186 countries (2020)
	Donation to UNODC	General purpose fund/GDP per capita	UNODC	186 countries (2020)
		Special purpose fund/GDP per capita		
		Pledges/GDP per capita		
	FATF Membership	The Financial Action Task Force Membership	FATF	186 countries (2020)

As discussed in last year’s report, data for donation to UNODC and FATF membership are limited, with historical data only being accessible from 2018. We have access to the other indicators’ data since 2010. Therefore, when we generated time series rankings from 2010 to 2019 in our previous report, there was a gap between 2017 and 2018 due to two new indicators that were included; thus making a comparison of results more difficult. To address this issue, last year’s report featured two versions of rankings: one with all fourteen indicators that provided an exact analysis of each country’s performance over that period but had a gap between 2017 and 2018 results as mentioned before; and another with thirteen indicators that did not include donations or FATF membership allowing for accurate tendencies of countries’ ranking across this timescale without any gaps. This year’s report follows the same guidelines, creating both versions for easier audience comparison when analyzing 2020 alongside our earlier findings from 2010 to 2019.

#### Results

This section reports the ranking results of the countries’ contribution to global justice from the perspective of cross-national criminal police cooperation. Please see the Tables 10 and 11 below for detailed ranking.

**Table 10:** Country ranking in cross-national criminal police cooperation (version 1)

Country	Ranking	Country	Ranking
Japan	1	Eswatini	94
United States of America	2	Burkina Faso	95
Germany	3	Morocco	96
Sweden	4	Nicaragua	97
United Kingdom of Great Britain and Northern Ireland	5	Cote d’Ivoire	98
Belgium	6	Kuwait	99
Italy	7	Jordan	100
Greece	8	Cuba	101
Brazil	9	Ghana	102
Finland	10	Slovakia	103
Canada	11	Republic of Moldova	104
India	12	Republic of North Macedonia	105
Austria	13	Monaco	106
New Zealand	14	Democratic Republic of the Congo	107
Norway	15	Yemen	108
Luxembourg	16	Malawi	109
China	17	Czechia	110
Netherlands	18	Ethiopia	111
Switzerland	19	Zambia	112
Russian Federation	20	Myanmar	113
Portugal	21	Cabo Verde	114
Mexico	22	Namibia	115
Israel	23	Armenia	116
France	24	Albania	117
Argentina	25	Bosnia and Herzegovina	118
Turkey	26	Serbia	119
Australia	27	Angola	120
Denmark	28	Malta	121
Indonesia	29	Lebanon	122
Spain	30	Suriname	123
South Africa	31	Brunei Darussalam	124
Togo	32	United Arab Emirates	125
Chile	33	Liberia	126
Ireland	34	Bahamas	127
Republic of Korea	35	Kenya	128
Senegal	36	Iraq	129
Jamaica	37	Sao Tome and Principe	130
Panama	38	Lao People’s Democratic Republic	131
Egypt	39	Papua New Guinea	132
Philippines	40	Djibouti	133
Malaysia	41	Mongolia	134
El Salvador	42	Nepal	135
Lesotho	43	Zimbabwe	136
Haiti	44	Bahrain	137
Saudi Arabia	45	Guinea-Bissau	138
Dominican Republic	46	Viet Nam	139
Poland	47	Gambia	140
Uganda	48	Qatar	141
Honduras	49	Burundi	142
Singapore	50	Botswana	143
Guatemala	51	Central African Republic	144
Mauritius	52	Saint Vincent and the Grenadines	145
Bolivia (Plurinational State of)	53	San Marino	146
Colombia	54	Niger	147
Costa Rica	55	Guinea	148
Iran (Islamic Republic of)	56	Timor-Leste	149
Ukraine	57	Comoros	150
Hungary	58	Congo	151
Belarus	59	Bhutan	152
Trinidad and Tobago	60	Slovenia	153
Tunisia	61	Saint Lucia	154
Ecuador	62	Nauru	155
Gabon	63	Estonia	156
Bulgaria	64	Somalia	157
Iceland	65	Uzbekistan	158
Cyprus	66	Kazakhstan	159
Algeria	67	Georgia	160
Madagascar	68	Equatorial Guinea	161
Sierra Leone	69	Cambodia	162
Nigeria	70	Bangladesh	163
Cameroon	71	Guyana	164
Peru	72	Dominica	165
Paraguay	73	Antigua and Barbuda	166
Uruguay	74	Saint Kitts and Nevis	167
Mali	75	Micronesia (Federated States of)	168
Benin	76	Chad	169
Mozambique	77	Mauritania	170
Libya	78	Solomon Islands	171
Lithuania	79	Tajikistan	172
Seychelles	80	Marshall Islands	173
Barbados	81	Oman	174
Rwanda	82	Tonga	175
Thailand	83	Fiji	176
Pakistan	84	Maldives	177
United Republic of Tanzania	85	Andorra	178
Kyrgyzstan	86	Kiribati	179
Afghanistan	87	Vanuatu	180
Azerbaijan	88	Belize	181
Sudan	89	Samoa	182
Romania	90	Montenegro	183
Sri Lanka	91	Grenada	184
Croatia	92	Palau	185
Latvia	93	Tuvalu	185

**Table 11:** Country ranking in cross-national criminal police cooperation (version 2)

Country	Ranking	Country	Ranking
United States of America	1	Morocco	94
Japan	2	Malawi	95
Brazil	3	Nicaragua	96
China	4	Latvia	97
Germany	5	Cote d’Ivoire	98
United Kingdom of Great Britain and Northern Ireland	6	Eswatini	99
India	7	Burundi	100
Italy	8	Ghana	101
Russian Federation	9	Kuwait	102
Mexico	10	Jordan	103
Canada	11	Ethiopia	104
Greece	12	Cuba	105
France	13	Zambia	106
Sweden	14	Slovakia	107
Togo	15	Republic of Moldova	108
Finland	16	Republic of North Macedonia	109
Chile	17	Czechia	110
Belgium	18	Myanmar	111
Philippines	19	Liberia	112
Egypt	20	Monaco	113
Turkey	21	Angola	114
Senegal	22	Cabo Verde	115
Austria	23	Namibia	116
Netherlands	24	Armenia	117
Norway	25	Lebanon	118
New Zealand	26	Albania	119
Iran (Islamic Republic of)	27	Bosnia and Herzegovina	120
Argentina	28	Serbia	121
Jamaica	29	Malta	122
Panama	30	Suriname	123
Luxembourg	31	Singapore	124
Switzerland	32	Brunei Darussalam	125
Indonesia	33	United Arab Emirates	126
Portugal	34	Central African Republic	127
Spain	35	Guinea-Bissau	128
Poland	36	Gambia	129
Lesotho	37	Nepal	130
El Salvador	38	Kenya	131
Haiti	39	Zimbabwe	132
Uganda	40	Iraq	133
Australia	41	Niger	134
Israel	42	Sao Tome and Principe	135
Dominican Republic	43	Bahamas	136
Honduras	44	Lao People's Democratic Republic	137
Madagascar	45	Papua New Guinea	138
Guatemala	46	Djibouti	139
Denmark	47	Somalia	140
Bolivia (Plurinational State of)	48	Mongolia	141
Mauritius	49	Viet Nam	142
Sierra Leone	50	Bahrain	143
South Africa	51	Iceland	144
Ukraine	52	Qatar	145
Nigeria	53	Guinea	146
Costa Rica	54	Botswana	147
Mozambique	55	Saint Vincent and the Grenadines	148
Algeria	56	Timor-Leste	149
Hungary	57	Comoros	150
Republic of Korea	58	San Marino	151
Belarus	59	Congo	152
Tunisia	60	Bhutan	153
Ecuador	61	Uzbekistan	154
Mali	62	Slovenia	155
Trinidad and Tobago	63	Saint Lucia	156
Colombia	64	Nauru	157
Cameroon	65	Estonia	158
Gabon	66	Cambodia	159
Bulgaria	67	Bangladesh	160
Afghanistan	68	Georgia	161
Peru	69	Kazakhstan	162
Pakistan	70	Chad	163
Cyprus	71	Equatorial Guinea	164
Benin	72	Guyana	165
Sudan	73	Dominica	166
Rwanda	74	Antigua and Barbuda	167
Paraguay	75	Saint Kitts and Nevis	168
Thailand	76	Tajikistan	169
Libya	77	Micronesia (Federated States of)	170
Ireland	78	Mauritania	171
Uruguay	79	Solomon Islands	172
United Republic of Tanzania	80	Marshall Islands	173
Kyrgyzstan	81	Oman	174
Lithuania	82	Tonga	175
Seychelles	83	Fiji	176
Barbados	84	Maldives	177
Burkina Faso	85	Andorra	178
Democratic Republic of the Congo	86	Kiribati	179
Azerbaijan	87	Vanuatu	180
Malaysia	88	Belize	181
Romania	89	Samoa	182
Sri Lanka	90	Montenegro	183
Yemen	91	Grenada	184
Saudi Arabia	92	Palau	185
Croatia	93	Tuvalu	185

In the first version, the top 10 countries in 2020 were Japan, the United States of America, Germany, Sweden, the United Kingdom of Great Britain and Northern Ireland, Belgium, Italy, Greece, Brazil, and Finland.

#### Regional Analysis

**Fig. 5:**
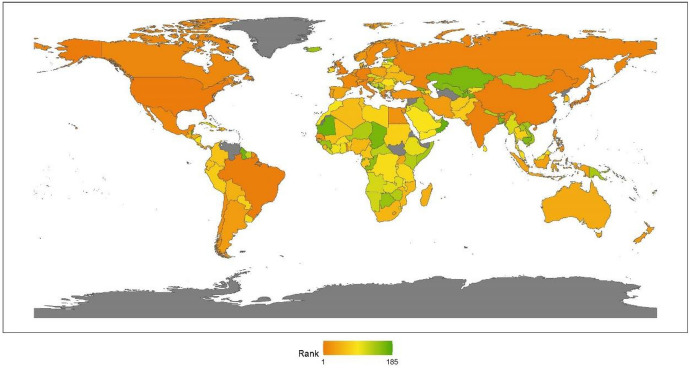
2020 index ranking of cross-national criminal police cooperation on a world map

This section provides a regional analysis of the ranking of cross-national criminal police cooperation (Fig. 5).

Asia: Generally, Asian countries performed well on this issue in 2020. Japan was actively engaged in cross-national police cooperation to combat transnational crime. In 2020, it ratified the Hague Convention on International Cooperation in Criminal Matters and signed a Memorandum of Understanding with the United States to increase information sharing between the two countries. It also provided assistance to developing countries to enhance capacity building for criminal justice administration, assisting victims of trafficking, and providing seminars on transborder crimes, such as drug enforcement and money laundering prevention.

Europe: There were seven European countries among the top 10 on this issue: Germany, Sweden, the United Kingdom, Belgium, Italy, Greece, Brazil, and Finland. In particular, the Federal Criminal Police Office of Germany took part in several international initiatives, including Europol’s Internet Referral System and Interpol’s I-24/7 system, which allow countries to rapidly exchange information on criminal matters. Additionally, it has signed a number of bilateral agreements with other European Union member states and non-EU countries to facilitate international investigations into criminal activity and improve data sharing capabilities between law enforcement agencies.

In 2020, the European Union (EU) took significant strides in cross-national police cooperation. The EU strengthened its data exchange capabilities and established a new system, called PULSE, to facilitate information sharing between different countries’ law enforcement agencies. In addition, it created an online platform, ENFOPOL 118, that allows for easy collaboration and coordination among all member states when tackling transnational crime. Furthermore, the recently ratified Prüm Convention provides a legal framework for easier cross-border investigations of criminal activity as well as improved access to DNA analysis and fingerprint databases across Europe.

North America: Both of the US and Canada performed well. In 2020, the United States continued to prioritize cross-national police cooperation to combat transnational crime. The US signed a number of bilateral agreements with countries across multiple continents seeking to improve information sharing between law enforcement agencies and facilitate international investigations into criminal activities. Additionally, they have become actively involved in initiatives such as Interpol’s I-24/7 system and Europol's Internet Referral System, which allow for rapid exchange of data on criminal matters.

Latin America: Transnational crime has been a major problem in Latin America. This is largely due to the lack of economic opportunity in many Latin American countries, coupled with high levels of corruption and a weak rule of law. The geographic location of Latin America, combined with its weak political and economic infrastructure, makes it a prime target for international criminal networks. Its proximity to both the US and Europe, two of the world’s wealthiest and most developed regions, creates an ideal market for illicit activities. In addition, the lack of investigatory resources and enforcement capabilities in Latin American countries make it easier for transnational crime organizations to get away with their activities.

Latin American countries have partnered with other countries in the region, as well as with organizations such as the United Nations, to form initiatives focusing on combating transnational crime. For example, in 2020, Chile and Peru joined forces to create a regional program to detect and prevent human trafficking. Mexico and Colombia signed an agreement to combat drug trafficking and organized crime. Brazil and Uruguay set up a joint task force to identify and prosecute cybercrime. Finally, Argentina and Uruguay have developed and are implementing a new program to strengthen maritime security and prevent illicit activities.

Africa: Due to the weak economic infrastructure and lack of rule of law in many countries in Africa, transnational crime has also been a major problem there. In 2020, several African countries out initiatives to combat transnational crime into practice. For example, in June 2020, the African Union launched the Africa-Wide Anti-Terrorism Campaign in response to the growing threat of terrorism and transnational organized crime in the region. This campaign seeks to strengthen cross-border cooperation, increase intelligence sharing, and identify terrorist networks within the region. As part of this campaign, delegates from all African states agreed on a 20-point plan to combat terrorism and transnational crime in the region, including measures such as improving regional legal frameworks, enhancing information exchange, and enhancing the capacity of national law enforcement agencies.

Oceania: Transnational crime is not as prevalent in Oceania as it is in other parts of the world, primarily because Oceania is relatively isolated. However, some issues connected with transnational crime still remain in the region, such as human trafficking, fraud, and cybercrime. In recent years, countries in the region have taken steps to strengthen their cross-border cooperation and enhance law enforcement capabilities to combat transnational crime. For example, in 2020, Australia, New Zealand, and Fiji launched the Pacific Regional Security Network, which is an information-sharing platform focused on combatting transnational crime. It includes a capability matrix that identifies areas of expertise, such as cybercrime and drug trafficking, that allows countries to quickly and effectively deploy resources to combat transnational crime. In addition, Australia, New Zealand, and Vanuatu signed a Memorandum of Understanding in 2020 to better coordinate law enforcement activities. The agreement focuses on strengthening communication and coordination between the three countries to combat transnational crime and promote regional security. Under the terms of the agreement, each country will share information and resources, including intelligence, surveillance capabilities, and personnel. Australia and the United States also established a joint Task Force to strengthen maritime security and disrupt illicit activity in the region.

#### Conclusion

In this section, we have established the performance and contribution of each country in relation to transnational crime by measuring its achievement in relation to 14 indicators. These include the ratification status of laws, donations to the Interpol and UNODC organizations, and FATF membership. In our results, European and North American countries generally had the highest scores, followed by Asia, Latin America, Africa, and Oceania. The US came out on top in our evaluation. The top 10 countries in 2020 were Japan, the United States of America, Germany, Sweden, the United Kingdom, Belgium, Italy, Greece, Brazil, and Finland. The top 10 countries in 2020 were Japan, the United States of America, Germany, Sweden, the United Kingdom, Belgium, Italy, Greece, Brazil, and Finland.

### Issue 6: Refugees

#### Introduction

2020 was a year of crisis for global refugee governance. The number of people forced to flee their homes due to war, violence, persecution, and human rights abuses rose to nearly 82.4 million, a 4% increase from the all-time high figure of 79.5 million in 2019.^57^ This means that 1% of the world's population was forcibly displaced during 2020. At the same time, the COVID-19 pandemic has enforced delays in the motion and resettlement of refugees, as countries closed their borders and reduced their hosting quotas, making it difficult to enable refugees to gain from their basic rights to education, health services,^58^ employment, and freedom of movement. Statistics released by the UNHCR show that only 22,770 people displaced abroad were resettled through UNHCR programs in 2020, a 20-year record low, although some 1.44 million refugees globally are in urgent need of resettlement.^59^ A growing number of refugees, many of whom are women and children, are struggling to survive, with one million children even being born as refugees.

The refugee problem is a crisis of global governance that threatens the achievement of global justice. Nation-states play a critical role in refugee protection. However, the burden and contribution of nation-states have been highly uneven. By the end of 2020, only 12 countries worldwide had sent 88% of the global refugee population. The top three countries as sources of highest numbers of refugees are Syria, with 6.7 million, Venezuela with 4 million, and Afghanistan with 2.6 million. On the other side, 16 countries (mostly developing countries) hosted 65% of the world’s refugees by the end of 2020. The top three countries with the largest number of hosted refugees are Turkey with 3.7 million, Jordan with 3 million, and Colombia with 1.7 million. While most international media headlines focus on the refugee influx crisis in the United States, the United Kingdom, and continental European countries, 73% of refugees nowadays are actually staying in nation-states that are neighboring their country of origin. Moreover, resettlement and welfare policies for refugees have even sparked social conflicts, political divisions, and party polarization in some Western countries, which has further complicated global refugee governance. Meanwhile, the COVID-19 pandemic has delivered huge economic and social shocks to developing countries, and many of them lack the capacity to deal with the health crisis, let alone ensure the survival and health of refugees with inclusive responses.

However, many nation-states and international organizations have made meaningful progress in helping protect refugees, including but not limited to receiving refugee status applications and conducting resettlement reviews through innovative online channels, providing vaccines and dedicated budgets to help refugees cope with the pandemic, contributing to the UNHCR (nation-states raised a total of $276.6 million funds in 2020), and mitigating regional conflicts by developing domestic and international cooperation, among other actions. These have had an encouraging positive effect in mitigating the continued growth in the number of refugees and the worsening of the global refugee crisis.

The efforts and contributions of a range of nation-states to global justice in the field of refugee governance vary widely. To assess the latest developments in global refugee governance and urge nation-states to put aside their differences and engage more deeply in refugee issues with a greater political will, our Global Justice Index report for 2020 continues to include a refugee sub-index. This sub-index was designed and calculated to rank nation-states according to their level of performance of and contribution to global justice in the issue area of refugee management. As a truly “whole-of-international community affair,” coverage of this issue area closely follows the principle of Common but Differentiated and Respective Capabilities (CBDR-RC).^60^ In other words, we take into account population size and economic power of different nation-states when constructing the index.

#### Dimensions and Indicators

We continue to follow the previous year’s approach to index construction methodology and indicator selection, which is consistent with other issue areas in this report. This sub-index comprehensively assesses the influence of nation-states on global justice in the field of refugee governance in two categories, namely, performance and contribution. Specifically, the performance category measures a country’s actions governing the creation and export of refugees, weighted by the size of the country’s population, i.e., the number of refugees exported per 1000 population. In the contribution category, we continue last year’s measurement by introducing five dimensions to evaluate a country’s efforts and investments in protecting incoming refugees and improving refugee governance. The five dimensions are as follows: (1) the number of refugees a country hosts and resettles relative to per log GDP (it is assumed that a country’s ability to host refugees is highly correlated with its economic power); (2) implementation of RSD (refugee status determination), measured by the number of decisions made on refugee status and the percentage of positive ones made; (3) participation in global refugee governance, measured by the membership of UNHCR and signatories to international agreements, including the 1951 Convention relating to the Status of Refugees and its 1967 Protocol; (4) refugee governance system, measured by indicators such as institutions for receiving, processing and identifying refugees, planning for displaced populations, measures to provide assistance, risk reduction strategies, and permission for temporary stay or temporary protection; and (5) the living conditions of refugees, measured by the quality of accommodation provided to refugees by a country.

Our data sources, data imputation approach, and index construction methods remain consistent with those of last year. Our data are mainly drawn from various authoritative international organizations, including the World Bank, UNHCR Statistical Yearbook, UNHCR-Annex of Global Appeal, and UN Report of World Population Policies (see Table 12). The issue of missing data remains a major challenge in conducting the index measurements. We followed last year’s data imputation method, which is of course imperfect and subject to potential error, but we used a variety of robustness tests to ensure maximum accuracy and reliability.

**Table 12:** Data on refugee

Category	Dimension	Indicators	Data source	Country coverage
Performance	Refugee population	Exported refugee population to per 1000 inhabitants	World Bank; UNHCR Statistical Yearbook	192 countries
Contribution	Refugee population	Imported refugees to per log (GDP)	World Bank; UNHCR Statistical Yearbook	211 economics
	Implement refugee status determination	Number of decisions made Proportion of positive decisions	UNHCR Statistical Yearbook	169 countries
	Participation in international refugee governance	Membership of UNHCR Signing international agreements	UNHCR-Annex of Global Appeal	192 countries
	National policies on refugee issues	System for receiving, processing, and identifying refugees; Planning for displaced populations; Specific measures to provide assistance; Disaster risk reduction strategy; Grant permission for temporary stay or temporary protection	World Population Policies	92–102 countries
	Standard of living	Type of refugee accommodation	UNHCR Statistical Yearbook	122 countries

#### Results

Using the unified index construction method developed for this project, this sub-index ranks the degree to which 192 nation-states around the world influence global justice in the issue area of refugee governance in 2020 (see Table 13), based primarily on the performance and contribution made by nation-states in this area. This index, along with the indices for the other nine issue areas of this report, make up the final Global Justice Index.

**Table 13:** Country ranking in refugee aspects of promoting global justice in 2020

Country	Ranking	Country	Ranking
Spain	1	Turkey	97
Sweden	2	Oman	98
France	3	North Macedonia	99
United Kingdom	4	Nigeria	100
Finland	5	Nepal	101
Germany	6	Liberia	102
Canada	7	Namibia	103
Brazil	8	Ethiopia	104
Ireland	9	Cote d’Ivoire	105
Belgium	10	Congo, Rep	106
Argentina	11	Togo	107
Italy	12	Slovak Republic	108
Philippines	13	Monaco	109
Switzerland	14	Moldova	110
Zambia	15	Kuwait	111
Mozambique	16	Sierra Leone	112
Thailand	17	Guinea-Bissau	113
Austria	18	Kyrgyz Republic	114
Norway	19	Armenia	115
Paraguay	20	Brunei Darussalam	116
Uruguay	21	San Marino	117
Japan	22	Ukraine	118
Luxembourg	23	Djibouti	119
Malawi	24	Kiribati	120
Denmark	25	Belize	121
Lesotho	26	Niger	122
United States	27	Georgia	123
Greece	28	Montenegro	124
Tanzania	29	Cambodia	125
Peru	30	Guatemala	126
Malta	31	Zimbabwe	127
South Africa	32	Andorra	128
Slovenia	33	Hungary	129
Korea, Rep	34	Pakistan	130
Lithuania	34	Uzbekistan	131
Chile	36	Jamaica	132
Palau	37	Colombia	133
Kenya	38	Grenada	134
Romania	39	Guyana	135
Czechia	40	Dominican Republic	136
Portugal	41	Mauritius	137
Madagascar	42	Cameroon	138
Uganda	43	Yemen	139
Australia	44	Guinea	140
Latvia	45	Cabo Verde	141
Qatar	46	Gambia	142
Bangladesh	47	Azerbaijan	143
Netherlands	48	Lebanon	144
New Zealand	49	Bahrain	145
Costa Rica	50	Marshall Islands	146
Samoa	51	Iran, Islamic Rep	147
Iceland	52	Albania	148
Cyprus	53	Mauritania	149
India	54	Nauru	150
Mexico	55	Nicaragua	151
Jordan	56	Equatorial Guinea	152
Panama	57	Korea, Dem. People’s Rep	153
Israel	58	Seychelles	154
Russian Federation	59	Mongolia	155
Poland	60	Somalia	156
Belarus	61	Tonga	157
Estonia	62	Mali	158
Ghana	63	Bahamas, The	159
Benin	64	El Salvador	160
Vanuatu	65	Serbia	161
Fiji	66	Sao Tome and Principe	162
Tunisia	67	Dominica	163
Angola	68	Honduras	164
Tajikistan	69	Antigua and Barbuda	165
Malaysia	70	Cuba	166
Botswana	71	St. Kitts and Nevis	167
China	72	Bosnia and Herzegovina	168
Papua New Guinea	73	Croatia	169
Ecuador	74	Central African Republic	170
Bulgaria	75	Afghanistan	171
United Arab Emirates	76	Libya	172
Algeria	77	Maldives	173
Egypt, Arab Rep	78	Iraq	174
Morocco	79	Congo, Dem. Rep	175
Burkina Faso	80	Barbados	176
Kazakhstan	81	South Sudan	177
Trinidad and Tobago	82	Sudan	178
Bolivia	83	Rwanda	179
Chad	84	Burundi	180
Timor-Leste	85	Vietnam	181
Eswatini	86	Venezuela, RB	182
Micronesia, Fed. Sts	87	Sri Lanka	183
Turkmenistan	88	Lao PDR	184
Gabon	89	St. Lucia	185
Solomon Islands	90	Syrian Arab Republic	186
Indonesia	91	Comoros	187
Senegal	92	Haiti	188
Suriname	93	St. Vincent and the Grenadines	189
Singapore	94	Eritrea	190
Saudi Arabia	95	Bhutan	191
Tuvalu	96	Myanmar	192

In 2020, the COVID-19 pandemic, with its accompanying various economic and social shocks, made global refugee governance extraordinarily challenging, bringing about myriad novel difficulties. On the one hand, the total number of refugees worldwide continues its decade-long upward trend, growing from approximately 26 million in 2019 to 26.4 million in 2020. Poor living conditions and inadequate health care have amplified the impact of the pandemic on crowed refugees, jeopardizing their livelihoods and posting gravest threats to their lives. On the other hand, the pandemic tested the ability of nation states and the international community to assist and protect refugees, as many of the protection measures and programs that existed before the pandemic could not be carried on successfully as they were. Many hosting countries failed to include refugees in their pandemic responses, and some turned to tighten their refugee admission and resettlement policies or even refused to accept additional asylum claims. Additional hardship was brought by long-persisting conflicts and erupting new ones, including a massive displacement of refugees from military and social conflicts in Syria, South Sudan, Central African Republic, Mozambique, Ethiopia, and elsewhere.

In 2020, the ranking of nation-states on the index of promoting global justice in the issue area of refugee governance remained generally stable relative to the data from the previous year (see Table 13), with some countries showing fluctuations in their order. The top 10 countries in the world, in order, are Spain, Sweden, France, the United Kingdom, Finland, Germany, Canada, Brazil, Ireland, and Belgium. The list of countries remains unchanged from the 2019 results, with only minor changes within the ranking of these countries. Western and Northern European countries still occupy 8 positions in the top 10, while the remaining 2 slots are from North America and Latin America. In 2020, the bottom-ranked 10 countries were Sri Lanka, Laos, St. Lucia, Syria, Comoros, Haiti, St. Vincent and the Grenadines, Eritrea, Bhutan, and Myanmar. In addition, Venezuela, Rwanda, and Sudan also performed poorly. Compared to the previous year’s results, the list has barely changed. This is primarily driven by prolonged political instability, poor human rights conditions, and weak state capacity in these countries, further exacerbated by the COVID-19 pandemic in 2020, which has only amplified their backwardness in the area of refugee governance.

#### Regional Analysis

**Fig. 6:**
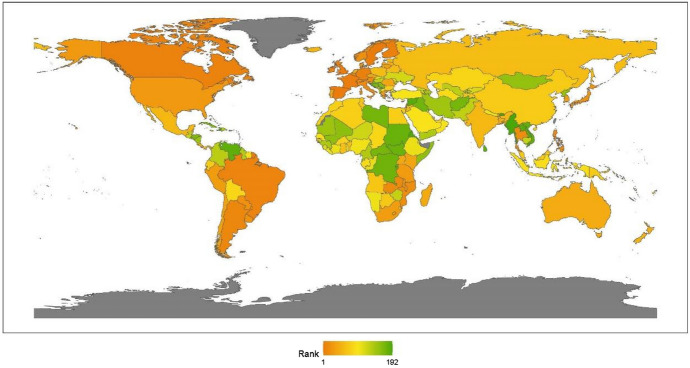
2020 index ranking of refugee governance on a world map

As with anti-poverty, refugee flows and their governing performance show a clear geographical clustering. A regional analysis of the composite indicators shows that North America and Europe were the most prominent contributors to global justice in the field of refugee governance in 2020, mainly driven by their lower number of refugee exports and thanks to their contribution in enhancing refugee protection and participation in the international protection regime during the pandemic crisis. However, as noted, this does not mean that the two regions host and resettle the largest number of refugees globally. Oceania, Latin America, Asia, and Africa are closer to each other in terms of their index scores. Relative to differences between continents broadly speaking, the variance is found to be more pronounced between sub-regions within continents (see Fig. 6). This is largely because the factors that produce refugees and determine the quality of refugee governance, such as military conflicts, natural disasters, political instability, and poverty, are often geographically specific within narrow regions.

Asia: Asia’s contribution to global justice in the area of refugee governance has been relatively low. In 2020, only 6 of the top 50 countries in the world refugee governance were from Asia: the Philippines (13th), Thailand (17th), Japan (22nd), South Korea (34th), Qatar (46th), and Bangladesh (47th). Within Asia, the refugee issues in Southeast Asia, South Asia, and the Middle East have been worrying. These regions both export a high number of refugees and make Asia a larger host of refugees (especially Turkey, Jordan, and Lebanon) because most of those forced from their homes fled to neighboring countries. Relative to other continents, Asia scores lower on the indicators of the number of RSD, proportion of positive decisions, and participation in the international refugee-protection regime, because many Asian countries still have not acceded to the 1951 Refugee Convention and its 1967 Protocol, continue with an impoverished refugee governance system, and made relatively few contributions to protecting and assisting refugees. By the same token, Asian countries have much room for progress in promoting global justice in the field of refugee protection, in both will and capacity. The international community should pay closer attention to sub-regions within Asia, providing more meaningful and sustainable support.

Europe: Europe, the preferred escape destination for refugees from Syria, North Africa, and elsewhere, has borne some of the brunt of the global refugee crisis. It has introduced various programs to support and protect refugees, both at the EU level and at the level of the EU member states. In 2020, Europe continued its excellent performance from previous years in the ranking of advancing global justice in the refugee governance, leading the world in the indicators of participation in international refugee conventions and of the number of RSDs. Within the region, Western and Northern Europe scored significantly higher than Eastern and Southern Europe, with 8 of the top 10 countries in the 2020 index coming from Western and Northern Europe, where they have long been at the top. Germany is the main refugee recipient in Europe, hosting a total of 1.2 million refugees as of 2020. The Eastern European countries Hungary, Serbia, Bosnia-Herzegovina, and Croatia continue to rank low. These countries have placed barriers at their borders to refugees trying to enter Europe through the Western Balkan corridor, even closing their borders completely during the pandemic.

North America: North America has been leading the world in promoting global justice in the issue area of refugee governance. However, compared to 2019, its overall index score decreased slightly in 2020. Specifically, Canada remained at seventh position globally, while the US’s ranking dropped from twenty-third in 2019 to twenty-seventh in 2020. Within North America, Canada contributes significantly more to global justice in the field of refugee governance than the United States does. Canada remains firmly in the top 50 on various indicators, including the number of refugees admitted, RSD and the percentage of positive decisions, refugee policy, and refugee living conditions. Canada is also signatory to international refugee conventions and actively participates in international refugee governance. For its part, the US scores lower on participation on the measure of international refugee conventions, and it has a lower percentage of positive cases in refugee status decision-making Likewise, it does not contribute as much as Canada does to providing accommodating conditions for the displaced, much of which is constrained by the increasingly polarized political ecology and growing anti-immigrant sentiment.

Latin America: Latin America continues to underperform in promoting global justice through refugee governance. Several countries in the region have been plagued by chronic natural disasters, political instability, economic collapse, and violent crime, forcing the displacement of millions of vulnerable people. The COVID-19 pandemic made this situation even more complex and volatile. By 2020, Venezuela alone had generated about 4 million refugees displaced across borders, making it the second-largest refugee outflow country in the world. Most Latin American countries actively participate in international refugee conventions (excepting only Cuba and Guyana), and they have also established a regional framework for refugee governance cooperation. In the 2020 rankings, there is a huge disparity within the region, with South American countries scoring significantly higher in the index than the counties in Central America and the Caribbean. For instance, Brazil ranked eighth and Argentina ranked eleventh in the world. By contrast, Haiti is ranked fifth from the bottom globally.

Africa: The refugee crisis in Africa remains very serious. The number of refugees in Africa has been growing for more than a decade, with one in three refugees worldwide being in Africa. In the 2020 ranking, Africa as a region is at the bottom of the list. While it scores high on two indicators, the number of refugees hosted and the proportion of positive outcomes of RSD, it underperform on other indicators. Within Africa, East Africa and the Great Lakes region hosted some 4 million refugees, with Ethiopia, Sudan, and Uganda in this region being among the African countries hosting the most people displaced abroad. In 2020, political unrest in Ethiopia and ongoing violent conflicts in Central Africa and South Sudan led to an influx of at least 120,000 refugees in Sudan. Numbers of refugees in West and Central Africa are also growing extremely quickly, driven mainly by floods, the pandemic, humanitarian crises, and intensifying military conflicts.

Oceania: Oceania showed moderate performance on all indicators of refugee governance. Its overall ranking in 2020 is only slightly higher than those of Africa and Asia. On the one hand, Oceania’s countries accepted a low number of refugees moving across their borders. On the other hand, the region does not stand out in terms of the number of RSD, the proportion of positive outcomes in RSD, or the provision of safe accommodation for refugees. This is partly determined by the region’s relatively isolated location. However, more importantly, the political will to engage in national refugee governance remains very low in these countries, most of which are not signatories to international refugee conventions. Within the region, Australia and New Zealand have contributed more than other Pacific Island countries. However, the growing numbers of Southeast Asian refugees having come in search of asylum in Australia and New Zealand in recent years have also come to meet increasing resistance from far-right forces and due to anti-immigrant sentiment in both countries.

#### Conclusion

The fulfillment of global justice is not possible to achieve while refugee populations continue to grow in the way that they are. Admittedly, the COVID-19 pandemic has exacerbated the worsening of the global refugee crisis and made it increasingly difficult for nation states and the international community to provide assistance and protection to displaced people. However, the pandemic also highlights the importance of strategic collaboration, the sharing of responsibility, and the inclusion of refugees in national responses and welfare systems, calling for joint efforts at both the global and regional levels to address various challenges to global governance that forced people to flee their homes. As Filippo Grandi, the High Commissioner of the United Nations Refugee Agency (UNHCR), said, “While the 1951 Refugee Convention and the Global Compact on Refugees provide the legal framework and tools to respond to displacement, we need much greater political will to address conflicts and persecution that force people to flee in the first place.”^61^

Addressing the refugee problems is a long-term and complex project. In addition to emergency support, such as housing and health care for refugees, the commitment of nation states and the international community to preventing and resolving military conflicts, political violence, economic crises, health crises, and humanitarian disasters of all kinds are necessary. Accomplishing this will allow people to live and work in peace and security without being displaced, and it will help more refugees return to their homelands. Security and hope are the best remedies.

### Issue 7: Anti-poverty

#### Introduction

The case of advancing global justice must first address the alarming problem of global poverty.^62^ For this reason, the UN’s 2030 Agenda for Sustainable Development places poverty eradication at the top of many agendas, urging nation states to take action against it. While national and global achievements in poverty reduction have been gratifying for more than a decade, the outbreak and continuation of the COVID-19 pandemic in 2020 have put the economy in many countries through the worst recession since the Great Depression, adding to global poverty. In developed countries, massive unemployment, reduced work hours, and uncertainty concerning the necessities of life and health have pushed millions of vulnerable people into the group of the new poor. In developing countries, declining in incomes due to the pandemic shock and the rise in the prices of food, energy, and household goods have exacerbated the status of the world’s poorest, increasing the difficulty for them of escaping the poverty trap, meanwhile dragging hundreds of millions of people who had already been lifted out of poverty to slide back into it. According to estimates from a World Bank study, “in 2020, between 88 and 115 million people could fall back into extreme poverty as a result of the Pandemic.”^63^ Global poverty reduction has thus recently suffered a major setback and may not even meet the poverty reduction mandate of the 2030 Sustainable Development Goals. This poverty crisis will no doubt deepen global inequality and jeopardize global justice.^64^ It is against this backdrop that our Global Justice Report continues to focus on and assess the achievement and contribution of countries and regions around the world in addressing extreme poverty in 2020, on the one hand tracking the latest developments in this issue area and on the other, urging nation states to make more targeted efforts to fight against poverty.

Nation states play a pivotal role in the global cause of poverty alleviation. Economic development, political stability, social welfare policies, and specific poverty alleviation programs as they are designed and carried out by nation states have a profound and positive impact on poverty governance.^65^ Our Global Justice Report suggests that nation states have a primary duty in the area of poverty alleviation. However, the progress, effectiveness, and contribution in the case of global poverty reduction vary greatly across countries. Some developing countries (e.g., China, Vietnam, and India) regard anti-poverty to be their political responsibility and moral task. As a result, a great deal of human, financial, and material resources are invested, and a series of deliberate institutions and policies are adopted to help their vulnerable citizens escape extreme poverty. However, there are also many less-developed countries that are constrained by their fragile state capacity and poor economic performance or that have been mired in political turmoil and military conflicts for too long to be able to center poverty alleviation on their agendas. In addition, with COVID-19 sweeping across the globe, the extent of the impact of the pandemic on the poor also varied across countries due to differing response strategies and control measures, thus exposing world populations to survival challenges and development opportunities. This has led to significant variance in anti-poverty performance across countries, which in turn have impacts on global justice.

#### Dimensions and Indicators

As the conceptualization and measurement of poverty is extremely complex and academically controversial, we continue to use the concept and theory of poverty that we applied last year. Consistent with the other issue areas of the Global Justice Index, we measure and rank nation-state scores on poverty governance (i.e., their contribution to global justice) in two core categories. This helps strike a balance between “thin” and “thick” concepts of poverty, without requiring a single monetary indicator to be used or the incorporation of an overly complex system of indicators.^66^

Continuing to use the previous year’s methodology, two categories are set: (1) contribution, measured by the reduction in the poverty rate, which assesses the degree of improvement in a country’s poverty reduction inputs in a given year compared to the previous year, benchmarked to the world average for that year. The formula is

$$\left\{\left({h}_{i}- {h}_{i-1}\right)- \left({\overline{h} }_{i}- {\overline{h} }_{i-1}\right)\right\}\times \mathrm{population}{}_{i}$$

where ${h}_{i}- {h}_{i-1}$ is the annual reduction of the poverty head ratio of a country, and ${\overline{h} }_{i}- {\overline{h} }_{i-1}$ describes the annual reduction of the poverty head ratio of the world as a whole. The difference between the two is the extent to which the reduction in the poverty head ratio of the country exceeds the world average, where the excess is taken to represent the country’s contribution to world poverty reduction and weighted by population. (2) Performance, a category directly measured by poverty gap data, assesses a country’s annual performance in reducing poverty.

Two points in particular need to be noted. First, the Poverty Reduction Index that is used in this report focuses on absolute poverty. Although absolute poverty is closely related to the issues of “inequality,” “relative poverty,” and “vulnerability,” they are often interchangeably discussed. However, they differ significantly in terms of their focus, concepts, measurement methods, and solutions. Following the project’s conceptualization of global justice, based on the principle of “cosmopolitan but due-diligent responsibility,” this report continues last year’s approach, focusing on the duty of diligence of nation states in addressing absolute poverty and improving the quality of life of their citizens in their jurisdictions.

Second, due to the differences in poverty lines adopted by different countries, it is common practice in the international community to make measurements using purchasing power parity (PPP) as a benchmark to facilitate cross-national comparisons. The World Bank has set three poverty thresholds based on 2011 PPPs: $1.90 per person per day (for low-income countries), $3.20 per person per day (for lower middle-income countries), and $5.50 per person per day (for upper middle-income countries); thus, this measures the minimum cost of goods and services that people must consume to maintain a basic subsistence and meet socially acceptable standards. In September 2022, the World Bank switched to the use of 2017 PPP to calculate global poverty data, and the nominal value of the new international poverty lines was readjusted from $1.90 in 2011 prices to $2.15 in 2017 prices, $3.20 to $3.65, and $5.50 to $6.85, respectively. However, this adjustment mainly pertains to the statistical criteria, “the real value of the international poverty line remains virtually unchanged.”^67^ In addition, as this year’s Global Justice Report focuses on countries’ contributions and achievement in the area of poverty governance in 2020, it will follow previous poverty lines. Our data sources, data imputation methods, index calculation procedures, and ranking rules continue last year’s practices. See Table 14 for specific data information and coverage.

**Table 14:** Data on anti-poverty

Category	Indicator	Data source	Coverage
Contribution	Poverty rate reduction ($5.5, population weighted)	World Bank	152 countries
Performance	Poverty gap ($5.5)	World Bank	152 countries

#### Results

Following the unified index construction methodology developed by this project, we obtain a total of 152 countries’ global justice ranking results with respect to their level of performance and contribution in the issue area of poverty reduction in 2020 (Table 15).

**Table 15:** Country ranking in anti-poverty aspect of promoting global justice in 2020

Country	Ranking	Country	Ranking
China	1	Indonesia	77
India	2	Colombia	78
Thailand	3	Bolivia (Plurinational State of)	79
Poland	4	Sri Lanka	80
Azerbaijan	5	Samoa	81
Switzerland	6	Gabon	82
Iceland	7	Tuvalu	83
Slovenia	8	Bhutan	84
Czechia	9	Fiji	85
Slovakia	10	Egypt	86
Cyprus	11	Armenia	87
Malta	12	Georgia	88
Finland	13	Iraq	89
Belarus	14	Tajikistan	90
Croatia	15	Maldives	91
Germany	16	Guatemala	92
Netherlands	17	Venezuela (Bolivarian Republic of)	93
Belgium	18	Cabo Verde	94
France	19	Pakistan	95
Norway	20	Myanmar	96
Malaysia	21	Philippines	97
Denmark	22	Kyrgyzstan	98
Luxembourg	23	Nicaragua	99
Russian Federation	24	Mauritania	100
United Kingdom of Great Britain and Northern Ireland	25	Botswana	101
Hungary	26	Sudan	102
Japan	27	Ghana	103
Republic of Korea	28	Honduras	104
Ireland	29	Namibia	105
Canada	30	Kiribati	106
Australia	31	Nepal	107
Lebanon	32	South Africa	108
Austria	33	Zimbabwe	109
Sweden	34	Comoros	110
Lithuania	35	Gambia	111
Bosnia and Herzegovina	36	Micronesia (Federated States of)	112
Kazakhstan	37	Vanuatu	113
Latvia	38	Cameroon	114
Uruguay	39	Bangladesh	115
Ukraine	40	Uzbekistan	116
Montenegro	41	Lao People’s Democratic Republic	117
Portugal	42	Haiti	118
Israel	43	Cote d’Ivoire	119
United States of America	44	Ethiopia	120
Seychelles	45	Solomon Islands	121
Turkey	46	Papua New Guinea	122
Spain	47	Angola	123
Serbia	48	Sao Tome and Principe	124
Bulgaria	49	Eswatini	125
Estonia	50	Senegal	126
Viet Nam	51	Guinea	127
Italy	52	Congo	128
Iran (Islamic Republic of)	53	Kenya	129
Republic of Moldova	54	Chad	130
Greece	55	Uganda	131
Mauritius	56	Yemen	132
Chile	57	Timor-Leste	133
Republic of North Macedonia	58	United Republic of Tanzania	134
Romania	59	Burkina Faso	135
Costa Rica	60	Sierra Leone	136
Jordan	61	Niger	137
Algeria	62	Togo	138
Panama	63	Liberia	139
Mongolia	64	Mali	140
Paraguay	65	Rwanda	141
Dominican Republic	66	Nigeria	142
Tonga	67	Benin	143
Morocco	68	Lesotho	144
Albania	69	Zambia	145
Tunisia	70	Mozambique	146
Brazil	71	Guinea-Bissau	147
Ecuador	72	Malawi	148
Peru	73	Democratic Republic of the Congo	149
Jamaica	74	Burundi	150
Mexico	75	Central African Republic	151
El Salvador	76	Madagascar	152

In the 2020 ranking, China and India maintained their momentum from previous years, leading the world in terms of contributing to global justice in the area of poverty governance, which is highly correlated with the large size of the poor population in both countries and the government’s proactive approach to poverty reduction. The other countries in the top 10 are Thailand, Poland, Azerbaijan, Switzerland, Iceland, Slovenia, the Czech Republic, and Slovakia. These countries achieved more than the world average in reducing the poverty head ratio and have performed well in addressing the poverty gap. With the exception of Vietnam,^68^ the ranking results have not changed much compared with 2019, although a slight back-and-forth adjustment can be seen in the positions of some countries, indicating the robustness of the index measurement.

China remains firmly at the top of the anti-poverty index; 2020 was the closing year of China’s national project of Targeted Poverty Alleviation. While the COVID-19 pandemic ravaging the world, the Chinese government was nevertheless able to persist in its efforts to achieve the poverty reduction target of the UN 2030 Agenda for Sustainable Development 10 years ahead of schedule. Admittedly, the pandemic certainly had an impact on poverty eradication in China. First, a large number of agricultural products could not be sold, which seriously reduced farmers’ income. Second, the rural labor force in poor areas was hindered from going out to work. Third, the pandemic blocked transportation, rural tourism, and rural industry development. In response, China’s central government provided 146.1 billion yuan of special funds for poverty alleviation in 2020,^69^ focusing on solving the “two no-worries and three guarantees” (*liangbuchou sanbaozhang*), i.e., to realize that the rural poor do not any longer need to worry about food and clothing and that compulsory education, basic medical care, and housing security are guaranteed; on the other hand, it increased targeted support for key poverty-stricken regions. Local governments also took multiple countermeasures to mitigate the impact of the pandemic and secure the achievement of poverty eradication by 2020: first, introducing e-commerce to options to promote the online sales of rural products; second, promoting local employment by financially supporting rural enterprises; third, strengthening the ability to find a precise match between labor supply and demand to broaden employment channels for migrant workers; fourth, creating more social welfare work to ensure minimum protection for the lowest income families; and fifth, providing timely relief and assistance to families that seem to be returning to poverty. By the end of 2020, all 832 poverty-stricken counties in China were lifted out of poverty, all 128,000 poor villages became unlisted, and nearly 100 million poor people escaped poverty. Accordingly, China declared success in the elimination of absolute poverty and regional poverty under its current standard.

For the 152 countries for which data and rankings are available, the bottom 10 countries in 2020 were Benin, Lesotho, Zambia, Mozambique, Guinea-Bissau, Malawi, Democratic Republic of Congo, Burundi, Central African Republic, and Madagascar, in that order. This result was largely unchanged from the 2019 ranking, suggesting that these countries made little improvements in anti-poverty spheres in 2020. It is an unfortunate fact that all of these countries are located in underdeveloped regions of Africa, which have long suffered from a number of adverse factors, such as a stagnant state economy, rapid population growth, military conflicts, political unrest, climate change, and humanitarian disasters, making their poverty relief extremely difficult, if not impossible. These bottom-ranked countries have significantly underperformed the world average in reducing the poverty head ratio and have performed poorly in addressing the poverty gap, thus failing to improve global justice in terms of either contribution or performance in the issue area of poverty governance. Even worse, during the COVID-19 pandemic, people in these countries met a compounded challenge due to poor health care and a worsening food crisis. In 2020, these countries experienced a regression, in which the number of new poor even exceeded the number of people escaping poverty. This significantly slows the process of global poverty reduction, and it inevitably threatens the achievement of global justice.

#### Regional Analysis

**Fig. 7:**
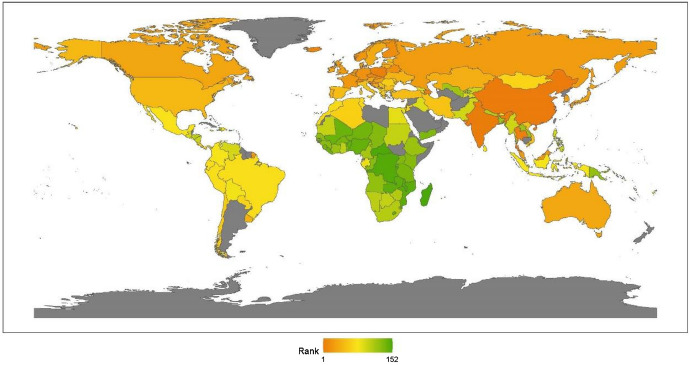
2020 index ranking of poverty governance on a world map

Poverty tends to be distributed in regional aggregations. Some regional factors (e.g., resource endowments or regional conflicts) constrain nation states from meaningfully contributing to global justice through poverty reduction. When ranked by region, the scores from highest to lowest in the issue area of poverty governance are, in descending order, North America, Europe, Latin America, Asia, Oceania, and Africa (see Fig. 7). This report suggests to achieve global justice requires regional cooperation and collaborative governance among nation states.

Asia: Poverty has always plagued Asia, and it has a higher rate of poverty head ratio and a larger poverty gap than North America or Europe, but this factor is declining: 2020 saw Asia make significant contributions to global poverty reduction, with China providing a prominent performance. While the pace of poverty reduction in Asia has slowed due to the pandemic, most countries in the region have maintained political stability and economic growth. At the same time, developing countries with large poverty populations (e.g., China, India, and Malaysia) continue to make substantial strides in addressing poverty, assuming the due-diligence obligations of nation states. The distribution of poverty within Asia is characterized by significant divergence, with East Asia ranking ahead of Southeast Asia, Central Asia, and West Asia. Harsh natural environments, ongoing regional conflicts, and insufficient state investment are the main reasons for the low ranking of some Central and West Asian countries.

Europe: Europe as a whole is firmly ranked second in the world on the Anti-Poverty Index. The poverty head ratio and poverty gap in Europe were already at a low level, and both fell in 2020. Within the region, Western and Northern Europe outperformed Eastern and Southern Europe in terms of contribution and performance for poverty governance. Iceland, Switzerland, Finland, Germany, and the Netherlands have long been among the world leaders in terms of their contribution to global justice on the issue area of poverty reduction, thanks to their established welfare state systems and their sustained economic vitality. Some Eastern European countries, including Poland, Slovenia, the Czech Republic, Slovakia, and Croatia, also achieved high rankings in 2020, with significant improvements in reducing the poverty head ratio and poverty gap. By contrast, Southern European countries (e.g., Spain, Italy, and Greece) remain at the bottom of the ranking within Europe, trapped by the European debt crisis and high unemployment, which was further exacerbated during the 2020 pandemic.

North America: In 2020, North America (including the United States and Canada) remained at the top of the anti-poverty rankings, significantly outperforming the global average in reducing the poverty head ratio and largely benefiting from the low variation within the region. While the US and Canada do not lead the world in the anti-poverty index as individual countries, as a region, they are not held back by underperforming countries. Within the region, Canada performs slightly better than the US in the index, with the two countries respectively ranking thirtieth and forty-fourth globally in 2020. Canada moved down one position relative to 2019, while the US moved up six positions. The 2020 pandemic disproportionately affected vulnerable groups within both countries. Targeted relief measures were introduced in both to effectively alleviate poverty, especially for children, the elderly, laborers, and the homeless. However, poverty among immigrants and minorities remains severe, largely due to growing political polarization and anti-migrant sentiment.

Latin America: Poverty has developed into a major governance challenge for Latin American governments. In 2020, Latin America (including 19 countries in Central and South America) continues to rank ahead of Asia and Africa. Within the region, South America achieved more than Central America and the Caribbean in controlling the rise in the poverty head ratio and poverty gap. This can largely ne attributed to the anti-poverty programs of Conditional Cash Transfers and other relief measures, which were widely implemented in major South American countries. However, although many countries in Central America and the Caribbean have also made considerable efforts to alleviate poverty, their achievements have been largely restricted by numerous natural disasters (e.g., earthquakes, hurricanes), fragile state capacity, political corruption, and economic slowdowns. In recent years, these countries have experienced sharp reductions in labor income and the worst recorded level of performance for the tourism industry, and some of them even became massive exporters of refugees. In 2020, the devastating socio-economic impacts of the COVID-19 pandemic have hit the anti-poverty cause in Latin America hard, with vulnerable people living in remote rural areas and in urban slums suffering new risks of reversing to poverty.

Africa: Africa has grappled with poverty and related problems for decades. It has long been at the bottom of regional-level index rankings. As noted, the 10 bottom-ranked countries in the 2020 Anti-Poverty Index are all located in sub-Saharan Africa. The shock of the onset of the COVID-19 pandemic in 2020 resulted in an increase both in the proportion of people living under poverty and in the overall poverty gap. Within Africa, North African countries score significantly ahead of sub-Saharan African countries on the Anti-poverty Index, with Morocco, Tunisia, and Egypt still ranking high in their sub-region, although they were in the middle of the global pack. By contrast, countries in Central, West, and Southern Africa have long lagged in contributing to global justice in the issue area of poverty governance. This is primarily driven by their rapid population growth, prolonged political unrest, and sluggish local economy. To make things worse, the deepening of social and health troubles prompted by the COVID-19 pandemic have driven millions of the already disadvantaged into even deeper poverty in these regions.

Oceania: Oceania ranks ahead of Africa but behind Asia in the Anti-Poverty Index. Australia has excelled in reducing the poverty head ratio and poverty gap, ranking 31st globally in 2020 for its contribution to global justice in terms of poverty governance. The Australian government has long been committed to an inclusive social security system, particularly for the Aboriginal poor, but welfare policies for immigrant groups have also been opposed by its far-right political forces. Island nations scattered across the South Pacific rank poorly, primarily because they suffer from an enclosed geography and the threat of sea-level rise from climate change. The pandemic has exacerbated the economic and social isolation of these countries.

#### Conclusion

Advancing global justice in the area of poverty governance requires nation states to diligently fulfill their obligations, through both enhanced domestic inputs and deepened regional cooperation. After the worldwide shock of the onset of the COVID-19 pandemic hit in 2020, the international cause of poverty alleviation was struck especially hard, with the poverty head ratio in many countries rising instead of falling, and the depth of poverty further expanding. In 2020, for the first time in decades, the number of new poor starts exceeded those lifted out of poverty, setting back the anti-poverty target of the UN’s 2030 Agenda for Sustainable Development.

Countries vary greatly in their anti-poverty measures and their government initiatives in response to the pandemic, which has further led to disparities in their performance in poverty alleviation across regions and countries. Thus, it is fair to say that the pandemic has even expanded the gap between countries in its contributions to global justice in the area of poverty alleviation. In the coming years, more targeted efforts are needed from nation states and the international community to protect the hard-won gains of poverty alleviation for the fronts of pandemic governance and economic recovery.

### Issue 8: Education

#### Introduction

Education is a fundamental human right, enshrined in the Universal Declaration of Human Rights^70^ and other international human rights treaties. In addition, education is crucial if other human rights are to be realized. Education can help break the poverty cycle, promote gender equality and create a more peaceful and sustainable world.^71^ It also significantly impacts health and well-being in both individuals and communities and contributes to the development of a more democratic, just, and inclusive society. Therefore, the question of justice for education not only concerns the development of education itself but also relates to the human fate and the living conditions of every educated person, which forms the bottom line of the education system and educational action. Educational justice includes the idea of fairness the administration of basic rights to education, equality of educational opportunities, fairness in the allocation of educational resources, and democracy in education governance.

The COVID-19 pandemic has had a significant impact on education in 2020. The sudden closure of schools and universities resulted in the widespread adoption of online and distance learning. However, this only widened the digital divide, as many students needed to acquire access to technology and the internet.^72^ The pandemic has also had a significant financial impact on educational institutions and highlighted existing educational system inequalities.^73^ The effects of the pandemic on education are likely to be long-lasting and will require sustained efforts to mitigate.^74^

This section assesses and compares the performance of diverse countries in their efforts toward promoting educational justice. This entails a detailed analysis of each country’s data to create a clear presentation of the current state of educational performance and government investment in education. In effect, this study contributes a unique perspective on delineating global justice.

#### Dimensions and Indicators

In line with previous reports, we here focus on two aspects of education to evaluate each country’s education situation from a global justice perspective. First, because basic education is widely regarded as a fundamental individual right, we focus on the performance of basic education to measure the extent of the protection of individual education rights. From the literature, we have drawn a set of indicators to model the performance of basic education, such as enrollment rate, completion rate, dropout rate, and pupil-teacher ratio. Second, the provision of basic education to the population is also widely seen as the government’s responsibility and duty. In response, we focus on the efforts made by governments to improve basic education, and we measure to what extent the government invests fiscal resources in basic education. The data framework and sources are shown in Table 16. Two main dimensions, focusing on the performance of primary education and lower secondary education, as well as financial contribution, provide different aspects of a country’s effort on education.

**Table 16:** Data on education

Category	Dimension	Indicator	Data source	Coverage
Performance	Primary education	Primary completion rate, total (% of relevant age group)	World Bank UNESCO; OCED	165 countries
		School enrollment, primary (% net)		
		Pupil-teacher ratio, primary		
		Children out of school (% of primary school age)		
	Secondary education	Lower secondary completion rate, total (% of relevant age group)		
		School enrollment, secondary (% net)		
		Pupil-teacher ratio, secondary		
		Children out of school (% of secondary school age)		
Contribution	Government expenditure on education	Government expenditure on education, total (% of GDP)		

The raw data are largely from the World Bank.^75^ The proportion of missing raw data is very high for some indicators, such as school enrollment and pupil-teacher ratio. To handle these missing data and expand the range of countries that can be ranked, we first collect the relevant data from other databases (i.e., UNESCO^76^ and OCED^77^) and replace some of the missing values using direct computation. Then, we adopt an autoregression model to impute the missing values from the other relevant indicators, beyond the nine indicators in the model, as dependent variables.

Armed with these data, we apply a population-weighted model to compute the education sub-index scores and rankings in terms of global justice. A population-weighted model is used to control for the influence of the population and reflects the country’s contribution and performance at an individual level.

#### Results

This section presents the overall ranking results for countries’ contributions to global justice in terms of education. Table 17 presents countries’ rankings for 2020. These rankings, in general, are relatively stable year-over-year. In this year’s report, we included 14 more countries in the educational justice ranking thanks to the improved model and the greater amount of available data. These were Bolivia, Greece, Morocco, Saudi Arabia, Luxembourg, Palau, Turkey, France, the Democratic Republic of the Congo, Nigeria, the Republic of Korea, Nauru, Solomon Islands, and Saint Kitts and Nevis. These changes also led the ranking to fluctuate slightly relative to previous results.

**Table 17:** Country rankings in the education aspect of promoting global justice in 2020

Country	Ranking	Country	Ranking
Luxembourg	1	Peru	84
United States of America	2	Fiji	85
Iceland	3	Ecuador	86
Switzerland	4	Namibia	87
Norway	5	Botswana	88
Denmark	6	Morocco	89
Sweden	7	Samoa	90
Australia	8	Azerbaijan	91
Israel	9	Bahamas	92
Belgium	10	Mongolia	93
Finland	11	Uzbekistan	94
Ireland	12	Trinidad and Tobago	95
Netherlands	13	Andorra	96
New Zealand	14	Suriname	97
Canada	15	Bhutan	98
Austria	16	Georgia	99
United Kingdom of Great Britain and Northern Ireland	17	Jamaica	100
Germany	18	Cambodia	101
France	19	Albania	102
China	20	Indonesia	103
Palau	21	Solomon Islands	104
Cyprus	22	Egypt	105
Japan	23	Philippines	106
Kuwait	24	Armenia	107
Qatar	25	Jordan	108
Saudi Arabia	26	Vanuatu	109
Malta	27	Ukraine	110
Italy	28	Guatemala	111
Singapore	29	Sri Lanka	112
Republic of Korea	30	Central African Republic	113
Estonia	31	Lesotho	114
Slovenia	32	Honduras	115
United Arab Emirates	33	Kyrgyzstan	116
Spain	34	Cabo Verde	117
Monaco	35	Timor-Leste	118
Czechia	36	Ghana	119
Portugal	37	Lao People’s Democratic Republic	120
San Marino	38	Viet Nam	121
Latvia	39	Eswatini	122
Marshall Islands	40	Cuba	123
Poland	41	Guyana	124
Slovakia	42	Algeria	125
Oman	43	Gambia	126
Costa Rica	44	Sierra Leone	127
Greece	45	Cote d’Ivoire	128
Lithuania	46	Myanmar	129
Croatia	47	Zambia	130
Hungary	48	Mauritania	131
Chile	49	Madagascar	132
Russian Federation	50	Burkina Faso	133
Uruguay	51	Burundi	134
Seychelles	52	Tajikistan	135
Argentina	53	El Salvador	136
Brazil	54	Rwanda	137
Antigua and Barbuda	55	Tunisia	138
Mexico	56	Kiribati	139
Saint Kitts and Nevis	57	Nepal	140
Barbados	58	Benin	141
Thailand	59	Togo	142
Turkey	60	Mozambique	143
Romania	61	Senegal	144
Panama	62	Chad	145
Malaysia	63	Bangladesh	146
Kazakhstan	64	Djibouti	147
Nauru	65	Mali	148
Iran (Islamic Republic of)	66	Guinea	149
Maldives	67	Cameroon	150
Mauritius	68	Congo	151
Bulgaria	69	Afghanistan	152
Belize	70	Malawi	153
Saint Vincent and the Grenadines	71	Niger	154
Belarus	72	Liberia	155
Sao Tome and Principe	73	Nicaragua	156
Colombia	74	Uganda	157
Dominican Republic	75	Democratic Republic of the Congo	158
Dominica	76	United Republic of Tanzania	159
Republic of Moldova	77	Kenya	160
Bolivia (Plurinational State of)	78	Lebanon	161
Saint Lucia	79	Ethiopia	162
Paraguay	80	India	163
Serbia	81	Nigeria	164
South Africa	82	Pakistan	165
Tonga	83		

In general, the top 10 countries were Luxembourg, the United States of America, Iceland, Switzerland, Norway, Denmark, Sweden, Australia, Israel, and Belgium. While the absolute rankings of several countries have changed, the differences in scores among these top countries were very small. With the exception of China, Palau, and the Marshall Islands, all of the countries in the top 50 are developed. On the other hand, developing countries, especially those with the lowest income, tended to rank toward the bottom. The lowest ranking countries were Nicaragua, Uganda, the Democratic Republic of the Congo, the United Republic of Tanzania, Kenya, Lebanon, Ethiopia, India, Nigeria, and Pakistan. Six of the lowest ranking 10 countries were in Africa, three in Asia, and one in Latin America.

Over the past decade, there has been a growth rise in worldwide expenditure on education. However, it appears that COVID-19 may have disrupted this consistent growth trajectory.^78^ However, in 2020, the average global government expenditure on education of GDP increased to 4.3% from 4.1% in 2019.^79^ There were broad differences between different regions of the world. The amount spent in high-income countries increased from 4.7% in 2019 to 5.2% in 2020, whereas that in the low-income countries fell to 3% by 0.2% from 2019.

#### Regional Analysis

**Fig. 8:**
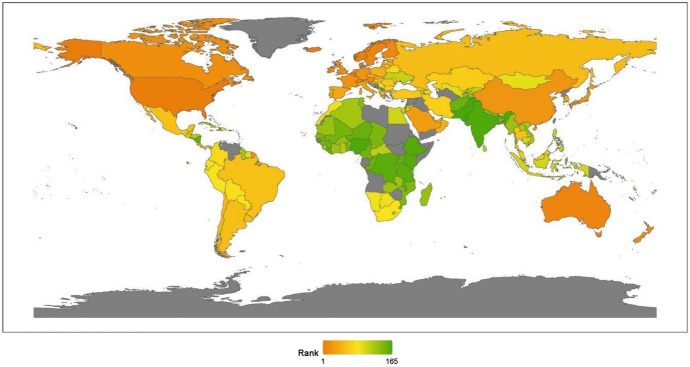
2020 index ranking of education issue on a world map

This section provides a regional analysis of the educational justice ranking. A visualization of the worldwide ranking in 2020 is depicted in Fig. 8, which allows us to see the regional differences in educational justice from a more intuitive point of view. From a continental perspective, the ranking from top to bottom was North America, Europe, Oceania, Asia, Latin America, and Africa. The remainder of this section discusses each continent and its sub-regions’ results in detail.

Asia: In general, Asia ranked in the middle of the continents. It gained one place relative to 2019’s ranking, mainly due to better basic education performance. However, the state of education in Asia is diverse and complex. This region is home to more than half of the world’s population, and its nations encompass a wide range of cultural, economic, and political contexts. Therefore, large gaps were seen among the sub-regions of Asia. Eastern Asia and Western Asia (the Middle East portion) reached the top tier in the education ranking, while South-Central Asia ranked toward the bottom of the list. The other parts of Asia, including Central, Western (other than the Middle East portion), and Southeast Asia, ranked in the middle. Specifically, nine of the top 20% countries for educational justice were located in Asia: three were in East Asia, five were in West Asia (the Middle East), and one was in Southeast Asia. On the other hand, seven countries were in the bottom 20%, of which six were in South-Central Asia and one in the Middle East.

In terms of performance, China (first), Iran (fifth), Thailand (sixth), and Japan (seventh) were among the top 10 list for Asia. As discussed in the last report,^80^ the population-weighted model weights a country's population positively if its performance in basic education is above the global average and negatively if it is below. Consequently, a country with a large population that outperforms the worldwide average in basic education might get a very high rating on this dimension. This is because the statistics show that the country is providing educational possibilities above the global average to a huge population. China and Japan were the representatives. On the contrary, the low ranking of Pakistan (165th) and India (163rd) suggested they provided their larger population with lower quality education.

In terms of contribution, only Israel (tenth) was on the top 10 list, and most countries were in the middle of the list. On average, governmental expenditures on education accounted for 4.09% of GDP, the same as the middle-income countries and slightly below the global average (4.3%). Within Asia, investment in education was the most unevenly distributed in the Middle East,^81^ with Israel spending $3171 per capita, 33 times as much as Lebanon, which spent $98 per capita.

Europe: European countries showed consistently high performance on education compared to other regions. All sub-regions were in the top 50% of the regional rankings, with Western and Northern Europe performing better than Southern and Eastern Europe. Of the top 10, 7 were European countries. The vast majority of its countries ranked in the top 50% of the global rankings, and the worst-performing country, Ukraine, also ranked as high as 110th (i.e., above the bottom 30%). The outperformance in this domain among European countries is mainly due to the high level of welfare and the high investment in education. However, relative to performance, in the population-weighted model, European countries, due to their relatively small populations, have no advantage in ranking.

Luxembourg is being observed in this reporting for the time and ranked first in 2020. Luxembourg has a well-regarded education system, considered as one of the best in Europe. The country places a strong emphasis on providing high-quality education to all its citizens, regardless of their background or socio-economic status. In 2020, its government expenditures on education accounted for 5% of GDP, resulting in $5833 per capita, the highest amount in the world. This high investment in education made it rank first in the contribution dimension. However, it did not perform very well in the performance dimension, with a middle ranking (i.e., seventy-seventh).

The lowest ranked countries in Europe, Ukraine (110th), Andorra (96th), and Albania (102nd), also had the most significant decline in this region. Their decreased performance was the cause of their decline in their ranking. For example, the primary school completion rate in Albania fell by 2.56%, and the children out-of-school rate increased by 1.74% relative to the previous year.^82^

North America: North America continued to have the highest average score on educational justice. Within the region, the United States ranked second worldwide, and Canada ranked fifteenth. In addition, the United States outperforms Canada in all three dimensions. The US ranked sixth on the contribution dimension and third on the overall performance dimension. Canada ranked seventeenth and ninth in the two dimensions.

On the contribution dimension, in 2020, the US and Canada increased their government expenditures on education. Specifically, the US increased its percentage of government expenditure on education in total GDP from 4.99% in 2019 to 6.05% in 2020. Canada increased its amount 4.77–5.17%. This positively impacts their ranking.

Latin America: Latin America ranked fifth, only slightly better than Africa. None of the countries were in the top 40, and most had middle rankings. The best-performing countries in Latin America were Costa Rica (forty-fourth) and Chile (forty-ninth). The worst-performing countries were El Salvador (136th) and Nicaragua (156th).

The ranking of Latin America countries was more strongly influenced by their rank in the contribution dimension. The average investment in education per capita in the region was $355, half of the global average.^83^ By contrast with the clustered ranking of the contribution dimension, the performance ranking was more dispersed. Six countries in Latin America ranked in the top 20%, and five countries were in the bottom 20%. Better performing countries, including Argentina, Brazil, Mexico, and Chile, had higher primary completion rates and lower dropout rates.

Africa: Overall, Africa performed the worst on educational justice in 2020. Within the region, Southern and Northern Africa had better performance than the other parts of Africa. Among the 43 African countries surveyed in this report, 39 countries ranked in the lower half of the list. The best-performing countries in Africa were Seychelles (fifty-second), Mauritius (sixty-eighth), Sao Tome and Principe (seventy-third), and South Africa (eighty-second).

All countries spent less than the world average on education, and the average dropout rates from basic education were the highest globally. Sub-Saharan Africa was the region with the highest levels of dropout. More than 20% of children aged between 6 and 11 dropped out, and around 33% of youths between 12 and 14 years were also out of school.^84^

Oceania: In general, Oceania ranked third out of the six continents. By sub-region, Australia and New Zealand ranked in the first tier, and Micronesia, Polynesia, and Melanesia ranked in the middle globally. By country, Australia, New Zealand, and Palau ranked in the top 20%, Kiribati ranked in the bottom 20%, and all of the other countries were middle-ranked in 2020.

The ranking of the two dimensions fell into roughly the same order as the overall ranking. The top-performing countries in this region were Palau (second), Marshall Island (eighth), and Australia (sixteenth). Regarding the government contribution to education, Australia (ninth), and New Zealand (fourteenth) were at the top of the overall list. However, countries varied widely in their expenditures on education. For example, in 2020, the government expenditure on education of GDP in Australia was about 6%, resulting in an outlay of $3,154 per capita; while in Vanuatu, the government only spent 2% of GDP, for $64 per capita.^85^

#### Conclusion

Education is a critical component in human rights and justice. It provides individuals with the knowledge, skills, and understanding necessary to participate fully in society and enjoy their rights. It also promotes social cohesion and helps create a more just and equitable society. By providing access to education for all, regardless of gender, ethnicity, or socio-economic background, societies can help break the cycles of poverty and inequality that perpetuate conflict and undermine justice.

In this study, we collected data from three reputable organizations: the World Bank, UNESCO, and OECD. Then, using a population-weighted model, we computed each country’s score in terms of basic education performance and the level of government investment in promoting educational justice on a global scale. The results showed that North America had the highest level of performance in 2020, whereas Africa exhibited the lowest. In addition, 14 countries’ data were included in this domain for the first time.

The COVID-19 pandemic has had a significant impact on education worldwide, and this must be considered when global educational justice is discussed. We identified falls in government expenditure on education in low-income countries and increases in high-income countries. The effects of the pandemic on education may have far-reaching consequences for other social issues, including public health, the economy, crime, and poverty. To fully understand the impact of COVID-19 on education, it is necessary to measure its effects and develop new indicators to reflect the influence of the pandemic on education. In the future, we should consider these additional indicators and modify the analytical model accordingly.

### Issue 9: Public Health

#### Introduction

Public health is widely recognized as a key issue in global justice.^86^ The right to health that means every person has the right to access the necessary health care and services to reach the highest possible level of physical and mental health.^87^ Maintaining public health is essential for promoting the well-being and health of all individuals, regardless of their socio-economic status or where they live. Improving public health, therefore, is among the necessary means to achieve a right to health. From the perspective of global justice, improving public health can help to decrease global health inequality. For example, providing access to basic healthcare and disease prevention measurements can help reduce disparities and improve overall health outcomes for people who live in poverty or in disadvantaged communities or disadvantaged countries.

Although both states and international organization have obligation with respect to the right to health. Implemented by improving global public health as a global justice issue,^88^ the primary obligation falls on states to take steps to respect, protect, and fulfill the right to health.^89^ Our aim is to evaluate the performance of countries on public health issues from the perspective of global justice, this report ignores the role of international organizations on public health issues and instead focuses on the countries’ performance on this issue.

Unlike previous reports, we included COVID-19 related indicators in this year's report. The COVID-19 pandemic swept the world in 2020, resulting in over 79 million confirmed cases and over 1.7 million deaths worldwide.^90^ To measure the impact of the COVID-19 crisis on countries’ performance on public health issues from the perspective of global justice, we have included COVID-19 related indicators in this report. However, it is worth noting that the COVID-19 crisis forms only a part of global public health issues. Therefore, we do not put COVID-19 in a prominent position in this report. Instead, we have placed it under the key disease dimension, together with other diseases in an equally important position.

We used a population-weighted method in calculating the score of the performance category. We first calculated the global average for each indicator as a benchmark. Then, based each country’s population, we transformed each indicator using the benchmark. Finally, we calculated each country’s score on this issue based on the transformed indicators. Following the population-weighted method, if a country performed better than the benchmark, the greater population this country has, the greater the total contribution of the country, and the higher its score. However, if a country performed worse than the benchmark, the greater the population, the lower its score. Therefore, a high ranking for a country on this issue would not necessarily mean that the country is leading in the world in terms of performance on this issue in absolute terms.

#### Dimensions and Indicators

Consistent with the Global Justice Index Report published in 2021, we measured each country’s performance on public health issues using two perspectives, namely, its performance in public health and its contribution to public health. The performance perspective mainly includes four dimensions, namely, life expectancy, mortality rate, public health infrastructure, and key diseases. Specifically, we use life expectancies at 60 years old and at birth to proxy for a country’s performance in terms of life expectancy. We also measure a country’s performance in terms of mortality using the adult mortality rate (for those aged between 15 and 60 years), infant mortality rate (for those aged between birth and 1 years), neonatal mortality rate, and under-five mortality rate to measure a country’s performance in terms of mortality rate. We also used two indicators, namely, the share of population using at least basic sanitation and the share of population using at least basic drinking-water services, to measure performance in terms of public health infrastructure. Finally, in addition to the indicators of treatment success rate of new tuberculosis (TB) cases, effective treatment coverage of tuberculosis, raised fasting blood glucose, and incidence of TB per 100,000 population per year, all of which were used in the 2021 report, we also added two new indicators, namely, the COVID-19 infection rate and the COVID-19 death rate, to measure a country’s performance in dealing with the COVID-19 pandemic in the dimension of key diseases. The data on COVID-19 come from Center for Systems Science and Engineering at Johns Hopkins University.^91^

This contribution measured countries’ effort to promote public health from the perspective of domestic general government health expenditures. The contribution category covered two indicators, namely, domestic general government health expenditure as a percentage of general government expenditure and domestic general government health expenditure per capita in US dollars. In addition to the COVID-19 related data, the other data are sourced from the WHO. The details can be found in Table 18.

**Table 18:** Data on public health

Category	Dimension	Indicator	Data source	Coverage
Performance	Life expectancy	Life expectancy at age 60 (years)	WHO	190 countries
		Life expectancy at birth (years)	WHO	
	Morality	Adult mortality rate (probability of dying between 15 and 60 years per 1000 population)	WHO	
		Infant mortality rate (probability of dying between birth and age 1 per 1000 live births)	WHO	
		Neonatal mortality rate (per 1000 live births)	WHO	
		Under-five mortality rates (probability of dying by age 5 per 1000 live births)	WHO	
	Public health infrastructure	Population using at least basic sanitation services (%)	WHO	
		Population using at least basic drinking-water services (%)	WHO	
	Key disease	Raised fasting blood glucose (≥ 7.0 mmol/L or on medication) (age-standardized estimate)	WHO	
		Treatment success rate: new tuberculosis cases	WHO	
		Tuberculosis effective treatment coverage (%)	WHO	
		Incidence of tuberculosis (per 100,000 population per year)	WHO	
		COVID-19 infection rate	JHU	
		COVID-19 death rate	JHU	
Contribution	Expenditure	Domestic general government health expenditure as a percentage of general government expenditure (%)	WHO	
		Domestic general government health expenditure per capita in US$	WHO	

#### Results

This section presents the ranking results for public health from the perspective of global justice in 2020. As shown in Table 19, which reports country rankings in public health for 2020, the top 10 countries are the USA, Norway, Germany, Japan, Ireland, Canada, Sweden, Denmark, Iceland, and the UK, which are the same countries as those identified as the top 10 in the 2019 report, only with slight changes in ranking. For example, Japan dropped from second place in 2019 to fourth place in 2020, while Canada rose from tenth place in 2019 to sixth place in 2020. Of the top 10 countries in public health, 7 are in Europe, 2 are in Northern America, and 1 is in Asia. Omitting COVID-19 related indicators, relating to the global public health crisis of 2020, we found that the top 10 countries remain stable. The largest change in the top 10 rankings is that Australia rose from eleventh to tenth, while Iceland fell from ninth to eleventh. The countries ranked eleventh to twentieth have remained stable. The largest change there comes from Palau, rising from thirty-seventh in 2019 to thirteenth in 2020, while Belgium fell from fifteenth in 2019 to twenty-first in 2020.

**Table 19:** Country rankings in the public health aspect of promoting global justice in 2020

Country	Ranking	Country	Ranking
United States of America	1	Algeria	96
Norway	2	Serbia	97
Germany	3	Saint Vincent and the Grenadines	98
Japan	4	Mauritius	99
Ireland	5	Namibia	100
Canada	6	Tunisia	101
Sweden	7	Bhutan	102
Denmark	8	Lesotho	103
Iceland	9	Trinidad and Tobago	104
United Kingdom of Great Britain and Northern Ireland	10	Burkina Faso	105
Australia	11	Saint Kitts and Nevis	106
New Zealand	12	Indonesia	107
Palau	13	Brunei Darussalam	108
Luxembourg	14	Malaysia	109
Netherlands	15	Viet Nam	110
Austria	16	Eswatini	111
France	17	Gabon	112
China	18	Solomon Islands	113
Costa Rica	19	Cabo Verde	114
Finland	20	Saint Lucia	115
Belgium	21	Nauru	116
Uruguay	22	Grenada	117
Italy	23	Niger	118
Switzerland	24	Albania	119
Iran (Islamic Republic of)	25	Sri Lanka	120
Spain	26	Mongolia	121
Malta	27	Fiji	122
San Marino	28	Rwanda	123
Israel	29	Philippines	124
Czechia	30	Tonga	125
Panama	31	Ukraine	126
Chile	32	Kiribati	127
Republic of Korea	33	Malawi	128
Maldives	34	Congo	129
Cuba	35	Burundi	130
Andorra	36	Tajikistan	131
Cyprus	37	Sudan	132
Singapore	38	Syrian Arab Republic	133
Bahamas	39	Papua New Guinea	134
Colombia	40	Madagascar	135
Tuvalu	41	United Republic of Tanzania	136
Portugal	42	Cambodia	137
El Salvador	43	Georgia	138
Nicaragua	44	Morocco	139
Estonia	45	Kenya	140
Slovenia	46	Bahrain	141
Argentina	47	Timor-Leste	142
Russian Federation	48	Turkmenistan	143
Paraguay	49	Kyrgyzstan	144
Guatemala	50	Iraq	145
Dominican Republic	51	Zambia	146
Slovakia	52	Armenia	147
Monaco	53	Senegal	148
Jamaica	54	Ghana	149
United Arab Emirates	55	Lao People’s Democratic Republic	150
Bosnia and Herzegovina	56	Mauritania	151
Saudi Arabia	57	Vanuatu	152
Ecuador	58	Dominica	153
Thailand	59	Libya	154
Suriname	60	Gambia	155
Antigua and Barbuda	61	Equatorial Guinea	156
South Africa	62	Mozambique	157
Lithuania	63	Guinea	158
Peru	64	Cote d’Ivoire	159
Bolivia (Plurinational State of)	65	Nepal	160
Qatar	66	Mali	161
Lebanon	67	Venezuela (Bolivarian Republic of)	162
Guyana	68	Togo	163
Poland	69	Egypt	164
Romania	70	Djibouti	165
Latvia	71	Zimbabwe	166
Greece	72	Angola	167
Botswana	73	Micronesia (Federated States of)	168
Mexico	74	Sierra Leone	169
Barbados	75	Benin	170
Bulgaria	76	Liberia	171
Belarus	77	Chad	172
Montenegro	78	Central African Republic	173
Kuwait	79	Comoros	174
Brazil	80	Haiti	175
Seychelles	81	Azerbaijan	176
Republic of North Macedonia	82	Afghanistan	177
Croatia	83	Cameroon	178
Oman	84	Myanmar	179
Turkey	85	Bangladesh	180
Uzbekistan	86	Guinea-Bissau	181
Hungary	87	Eritrea	182
Belize	88	Democratic Republic of the Congo	183
Samoa	89	South Sudan	184
Jordan	90	Yemen	185
Honduras	91	Ethiopia	186
Republic of Moldova	92	Uganda	187
Sao Tome and Principe	93	Pakistan	188
Kazakhstan	94	India	189
Marshall Islands	95	Nigeria	190

The bottom 10 countries of the ranking in this issue are Guinea-Bissau, Eritrea, Democratic Republic of the Congo, South Sudan, Yemen, Ethiopia, Uganda, Pakistan, India, and Nigeria. Once again, similar to the ranking of last year’s report, the ranking of the bottom four countries remained relatively stable. The only change is that Guinea-Bissau replaced Cameroon to enter the bottom 10 countries, while Cameroon rose from the third from the bottom in 2019 to the thirteenth from last in 2020. Of the bottom 190 countries, 7 countries are in Africa, and the remainder are in Asia. After excluding the COVID-19 related indicator, we found that our results are robust: there were no changes in the ranking of the countries in the bottom 10, only slight changes in their ranking.

#### Regional Analysis

**Fig. 9:**
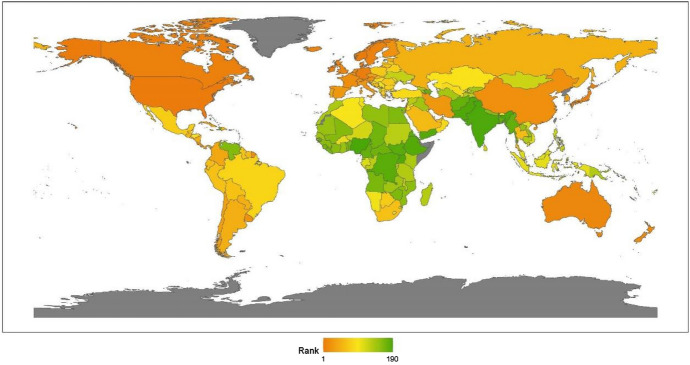
2020 index ranking of public health issues on a world map

In this section, we provide a regional analysis of the ranking in public health issues from the perspective of global justice. Figure 9 shows the geographic distribution of the rankings across countries in 2020. It is very clearly shown that North America, Europe (especially Northern and Western Europe), the Australian and New Zealand region in Oceania, and the East Asian region rank higher, while Africa as a whole, Asia (especially South Asia), and the Caribbean region rank lower. The figure also clearly shows the great variation in ranking within the continents.

Asia: In 2020, Asia as a whole ranks slightly higher than Africa. According to Table 19, 27 of the 49 Asian countries in the ranking are ranked after the hundredth place. Furthermore, Yemen, Pakistan, and India ranked among the bottom 10 countries. Within Asia, however, there are large variations in rankings across countries. For example, among the 49 Asian countries in the ranking, 8 countries, namely, Japan (fourth), China (eighteenth), Iran (twenty-fifth), Israel (twenty-ninth), Republic of Korea (thirty-third), Maldives (thirty-fourth), Cyprus (thirty-seventh), and Singapore (thirty-eighth) rank within the top 20%. However, eight countries, namely Nepal (160th), Azerbaijan (176th), Afghanistan (177th), Myanmar (179th), Bangladesh (180th), Yemen (185th), Pakistan (188th), and India (189th), rank within the bottom 20%. From a regional perspective, East Asia is the best-performing region in Asia. In the four East Asian countries listed in Table 19, two (Japan (fourth) and China (eighteenth)) are ranked among the top 10% globally, and three (Japan, China, and Republic of Korea (thirty-third)) are ranked among the top 20% globally. South Asia is the worst-performing region in Asia on public health issues, and four (namely Nepal, Bangladesh, Pakistan, and India) of the eight countries in Asia that are ranked among the bottom 20% globally are from South Asia.

Europe: Europe as a whole ranks second to North America in terms of public health. In Table 19, it can be seen that 7 of the top 10 countries in terms of public health are from Europe. Among the 42 European countries listed in Tables 19 and 20 are ranked among the top 20% worldwide on this issue, and 39 are ranked among the top 50%. The lowest ranked country in Europe is Ukraine, which is ranked 126th.

**Table 20:** Data on the protection of women and children

Category	Dimension	Indicator	Data source	Coverage
Performance (women)	Health and demography	Life expectancy at birth, ratio female to male(years)	World Bank	163 countries
		Maternal mortality ratio (modeled estimate, per 100,000 live births)		
		Mortality ratio, under 5 years, female to male		
		Sex ratio at birth (male to female births)		
	Economic status	Unemployment ratio, female to male		
		Vulnerable employment, ratio of female to male		
		Wage and salaried workers, ratio female to male		
	Political status	Proportion of seats held by women in the national parliament (%)		
Performance (children)	Children’s health and demography	Number of deaths per 1000 + (include 13 indicators), under 5 years	WHO	
		Prevalence of thinness among children and adolescents, BMI < -2 standard deviations below the median (crude estimate) (%)	WHO	
	Children’s education (educational difference between males and females)	Gender parity index for gross enrollment ratio in primary and secondary education	World Bank	

Figure 9 clearly displays the regional disparities in public health rankings across Europe. In particular, Northern and Western Europe are the best-performing regions in Europe, and Eastern and Southern Europe perform relatively poorly. Of the 10 Northern European countries, 6 rank in the top 10 in the world, and 5 of the 8 Western European countries are ranked in the top 10% globally. However, none of the 10 Eastern European countries or the 14 Southern European countries ranked in the top 10% globally. Of the bottom 10 countries in Europe, 5 (Bulgaria (76th), Belarus (77th), Hungary (87th), Republic of Moldova (92nd), and Ukraine (126th)) are in Eastern Europe, and 5 (Montenegro (78th), Republic of North Macedonia (82nd), Croatia (83rd), Serbia (97th), Albania (119th)) are in Southern Europe.

North America: North America is made up of two developed countries, namely, the United States and Canada. This region is one of the best performing in the world for public health issues as relates to global justice. The United States ranks first in the world, and Canada ranks sixth.

As previously reported, the United States and Canada do not have the best performance globally in terms of public health in terms of performance. For example, in 2020, the United States was among the countries most severely affected by the COVID-19 pandemic. According to statistics from the Center for Systems Science and Engineering at Johns Hopkins University, the number of COVID-19 cases in the United States surpassed 20 million, for an infection rate of 6.1%. The number of COVID-19 deaths in the country was 350,000, 0.1% of the total population. However, in terms of contribution to global health, however, the two countries are at the forefront globally in terms of public health investment. For example, the United States and Canada rank third and sixteenth globally in domestic general government health expenditure as a percentage of general government expenditure, at 22.35% and 18.3%, respectively. Similarly, the United States and Canada rank fourth and ninth globally in domestic general government health expenditure as a percentage of gross domestic product, at 10.68% and 9.7%, respectively. Furthermore, the United States ranks first among the major global economies on both indicators.

Latin America: Latin America as a whole presented better performance on public health issues than Africa and Asia. Among the 33 Latin American countries in Table 19, more than 75% (25 countries) of the countries are in the top 50% in the world for public health issues. Only three countries (namely Dominica (153rd), Venezuela (162nd), and Haiti (175th)) are in the bottom 20% of the world’s rankings.

Like other regions in Europe and Asia, Latin America exhibits significant regional disparities on this issue. Central America performed better than South America, and the Caribbean region. The country with the best performance in Latin America on public health issues was in Central America (Costa Rica (nineteenth)), and all eight Central American countries are ranked in the top 50% in the world. The Caribbean region had the worst performance in Latin America on this issue. Of the eight Latin American countries ranked in the bottom 50% of the world on this issue, seven of them are in the Caribbean region.^92^

Africa: As a whole it had the worst performance of any continent on public health issues. South Africa had the highest ranking on public health issues on the continent, but it only ranked sixty-second in the world. Of the 53 African countries in Table 19, 49 ranked in the bottom 50% of the world on this issue. Of the 53, 26 African countries ranked in the bottom 20% of the world, and 12 ranked in the bottom 10% of the world.

Figure 9 also clearly showed that although Africa as a whole performs poorly on public health issues, it shows regional differences in performance across Africa. Southern Africa is the best-performing region in Africa on this issue, although its performance cannot be called good from a global perspective. The two countries in Southern Africa, South Africa and Botswana, are the best-performing countries in Africa, ranking sixty-second and seventy-third in the world, respectively. Other regions in Africa (East Africa, Central Africa, North Africa, and West Africa) do not exhibit significant differences in performance on this issue.

Oceania: The 14 countries of Oceania performed well as a whole in terms of public health issue, but the 14 countries show great differences in performance. For example, three countries (viz. Australia (eleventh) New Zealand (twelfth), and Palau (thirteenth)) in Oceania that rank in the top 10% in the world in terms of public health, but eight countries in the region rank in the bottom 50% in the world.

The Australia and New Zealand region in Oceania (consisting of two developed countries) has one of the best regional performance in public health in the world, but the regions of Melanesia, Micronesia, and Polynesia, all of which consist of Pacific island countries, perform poorly. Palau from Micronesia and Tuvalu from Polynesia are exceptions. This is largely due to the high scores of those two countries in terms of the contribution dimension, reflecting the efforts of that their governments have expended to improve public health in their own countries. For example, in 2020, Palau and Tuvalu ranked second and first in the world in terms of domestic general government health expenditure as a percentage of gross domestic product, reaching 13.51% and 18.09%, respectively. Likewise, domestic general government health expenditure as a percentage of general government expenditure were high as well, reaching 23.24% and 15.9%, respectively, ranking second and twenty-seventh in the world in the same year.

#### Conclusion

We collected 16 indicators on life expectancy, mortality, public health infrastructure, key diseases, and domestic general government health expenditure from WHO and Hopkins University and calculated the scores on this issue for 190 countries based on a population-weighted model. Our results show that developed countries perform better on this issue, but developing countries (especially those with large populations) perform less worse than previously. From a regional perspective, North America, Europe, and East Asia, together with some countries from Oceania, performed better, while Africa and South Asia performed worse.

Here, we only weight a country’s indicators for the performance category by its population. This means that when a country exceeds the global average in the indicators, having a large population size will lead to a higher score, and vice versa. Thus, our results are in line with the expectation that countries with large populations that perform better than the global average will rank high, and conversely, countries with large populations that do not perform as well as the global average rank low. In addition, because we give equal weight to the contribution category as to the performance category and do not transform the indicators in the contribution category with population weights, some countries that perform well in the contribution category also rank higher on this issue.

### Issue 10: Protection of Women and Children

#### Introduction

The protection of women and children is one of the 10 key issues of global justice in this report. These two groups are often marginalized and vulnerable populations that suffer disproportionate levels of discrimination and violence.^93^ This can result in unequal access to basic human rights, such as education, healthcare, and economic and political opportunities. Therefore, protecting women and children is crucial for promoting human rights and providing opportunities for development for disadvantaged groups, creating a fairer society. Protecting women and children is thus important for achieving global justice.^94^

Unlike other issues, for which we evaluate a country based on both performance and contribution dimensions, we can only assess a country's performance in promoting gender equality and protecting children from a performance point of view, due to the lack of information on government spending for issues related to protecting women and children.

For this issue, we use a population-weighted model to convert all indicators and calculate each country’s score. As we noted for the public health issue, the rankings are calculated based on this model to indicate the extent to which a country is enabling its share of the global population to live better than the global average. In other words, a high ranking for a country on this issue does not necessarily mean that the country is leading all those below it in terms of performance.

#### Dimensions and Indicators

To measure scores on this issue, we will separately measure its performance in protecting women and protecting children. On the one hand, to assess a country’s performance in protecting women, we use a set of indicators that cover women’s relative position in the country on health, demography, economics, and politics to measure its gender inequality. First, we will use a set of indicators, namely, gender ratios in life expectancy at birth, mortality under 5 years old, and maternal mortality, to measure the relative health status of females. Second, we used the gender ratio at birth to measure demographic characteristics. Third, we used a set of employment-related indicators, namely, the gender ratio in the unemployment ratio, the vulnerable employment ratio, wage, and in salaried workers, to measure the relative position of females in the economy. Last, we used the proportion of seats held by women in the national parliament to proxy for the political status of women. The women-related data were sourced from the World Bank.

On the other hand, we accessed performance in protecting children from two perspectives. First, we focus on child health, which involves two indicators: number of deaths per 1000 live births, defined as the number of deaths among children aged less than 5 years from a specific cause per 1000 live births, and the prevalence of thinness among children and adolescents, measured by the percentage of school-age children and adolescents with a body mass index (BMI) less than 2 standard deviations below the median. These indicators related to child health are sourced from the World Health Organization. Second, we use a gender parity index for gross enrollment ratio in primary and secondary education, defined as the ratio of girls to boys enrolled at primary and secondary levels in public and private schools, to proxy for gender inequality in children’s education. The indicator comes from the World Bank. More details can be found in Table 20.

#### Results

This section reports the ranking results for the protection of women and children from the perspective of global justice in 2020. As shown in Table 21, which reports the country rankings in the protection of women and children in 2020, the top 10 countries are China, the USA, Russia, Brazil, Mexico, Germany, the UK, Italy, and Spain. Of the top 10 countries, 6 are in Europe, two are from Latin America, and the other two are from Asia and North America. The top 10 countries in 2020 are almost identical to those for this issue in 2019. The only change in the top 10 counties between 2019 and 2020 is that Spain, which was ranked eleventh in 2019, has replaced Thailand (ranked fourteenth in 2020) to become the tenth in 2020.

**Table 21:** Country rankings in the protection of women and children in 2020

Country	Ranking	Country	Ranking
China	1	Montenegro	83
United States of America	2	Malta	84
Russian Federation	3	Luxembourg	85
Brazil	4	Iceland	86
Mexico	5	Suriname	87
Germany	6	Barbados	88
France	7	Saint Lucia	89
United Kingdom of Great Britain and Northern Ireland	8	Cabo Verde	90
Italy	9	Samoa	91
Spain	10	Belize	92
Ukraine	11	Guyana	93
Poland	12	Saint Vincent and the Grenadines	94
Argentina	13	Venezuela (Bolivarian Republic of)	95
Thailand	14	Paraguay	96
Republic of Korea	15	Tonga	97
Canada	16	Sao Tome and Principe	98
Iran (Islamic Republic of)	17	Vanuatu	99
Australia	18	Bahrain	100
Syrian Arab Republic	19	Azerbaijan	101
Viet Nam	20	Botswana	102
Turkey	21	Fiji	103
Colombia	22	Brunei Darussalam	104
Cuba	23	Solomon Islands	105
Saudi Arabia	24	Maldives	106
Romania	25	Bhutan	107
Netherlands	26	Comoros	108
Kazakhstan	27	Djibouti	109
Belarus	28	Tajikistan	110
Sweden	29	Timor-Leste	111
Belgium	30	Namibia	112
Uzbekistan	31	Eswatini	113
Portugal	32	Equatorial Guinea	114
Tunisia	33	Eritrea	115
Czechia	34	Turkmenistan	116
Jordan	35	Gambia	117
Chile	36	Rwanda	118
Greece	37	Guatemala	119
Austria	38	Oman	120
Hungary	39	Lesotho	121
Malaysia	40	Cambodia	122
South Africa	41	Morocco	123
Israel	42	Lao People’s Democratic Republic	124
Algeria	43	Mauritania	125
Dominican Republic	44	Madagascar	126
Sri Lanka	45	Burundi	127
Serbia	46	Congo	128
Finland	47	Liberia	129
Bulgaria	48	Malawi	130
Denmark	49	Papua New Guinea	131
Singapore	50	Zimbabwe	132
Slovakia	51	Nepal	133
Norway	52	Togo	134
Ireland	53	Myanmar	135
Switzerland	54	Central African Republic	136
Ecuador	55	Philippines	137
New Zealand	56	Uganda	138
Kyrgyzstan	57	Zambia	139
Costa Rica	58	Sierra Leone	140
Croatia	59	Benin	141
Kuwait	60	Ghana	142
Georgia	61	Guinea	143
Lithuania	62	Burkina Faso	144
Peru	63	Niger	145
Armenia	64	Kenya	146
Republic of Moldova	65	Yemen	147
Panama	66	United Republic of Tanzania	148
Senegal	67	Indonesia	149
Uruguay	68	Mali	150
Mongolia	69	Cameroon	151
Latvia	70	Bangladesh	152
Slovenia	71	Angola	153
Albania	72	Mozambique	154
North Macedonia	73	Chad	155
Estonia	74	United Arab Emirates	156
Qatar	75	Afghanistan	157
Nicaragua	76	Ethiopia	158
El Salvador	77	Egypt	159
Cyprus	78	India	160
Trinidad and Tobago	79	Democratic Republic of the Congo	161
Mauritius	80	Pakistan	162
Bolivia (Plurinational State of)	81	Nigeria	163
Honduras	82		

The bottom 10 countries in the ranking of 163 countries in 2020 are Mozambique, Chad, United Arab Emirates, Afghanistan, Ethiopia, Egypt, India, the Democratic Republic of the Congo, Pakistan, and Nigeria. Of the bottom 10 countries, six are in Africa and four are in Asia. The bottom 10 countries for this issue in 2020 are somewhat different from those in 2019. First, the three countries ranked at the bottom 10 in 2019, namely, Yemen, Mali, and Bangladesh, have risen from the 152nd, 153rd, and 154th in 2019 to the 147th, 150th, and 152nd places, respectively, so the following countries are no longer among the bottom 10 countries on this issue in 2020; second, Mozambique, Chad, and the United Arab Emirates have dropped from the 148th, 150th, and 137th place in 2019 to the 154th, 155th, and 156th positions in 2020, thus entering the list of the bottom 10 countries.

#### Regional Analysis

**Fig. 10:**
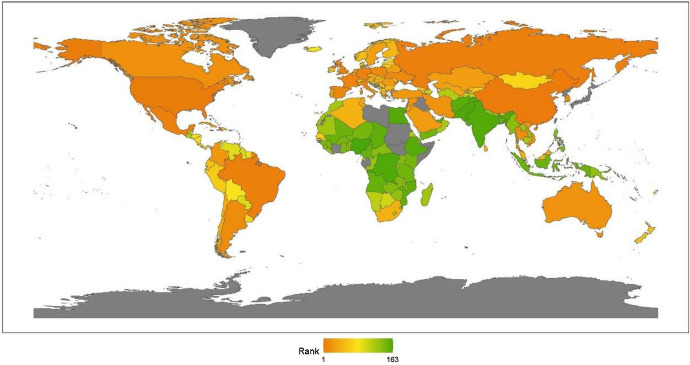
2020 index ranking of protection of women and children on a world map

This section provides a regional analysis of the ranking in the issue of the protection of women and children from the perspective of global justice. Figure 10 shows the geographic distribution of the rankings across countries in 2020.

Asia: There is a significant difference seen in the performance of the 43 Asian countries in the issue of protection of women and children, as shown in Table 21. First, in 2020, 10 countries, namely China, Thailand, Republic of Korea, Iran, Syrian Arab Republic, Viet Nam, Turkey, Saudi Arabia, Kazakhstan, and Uzbekistan, are ranked in the top 20% in the world, and 3 (China, Thailand, Republic of Korea) are ranked in the top 10%. Among these, China is ranked first in the world in this issue. Second, 10 countries, namely Nepal, Myanmar, Philippines, Yemen, Indonesia, Bangladesh, United Arab Emirates, Afghanistan, India, and Pakistan, are ranked in the bottom 20% in the world, and 7 countries, viz. Philippines, Yemen, Indonesia, Bangladesh, United Arab Emirates, Afghanistan, India, and Pakistan, are ranked in the bottom 10% in 2020. Second, from a regional perspective, East Asia is the best-performing region in Asia on this issue, and it is also one of the better performing regions in the world. Two countries from East Asia, China and Republic of Korea, ranked first and fifteenth in the world. By contrast, South Asia is the worst-performing region in Asia on this issue, and it is also one of the worst-performing regions in the world.^95^ Three countries from South Asia, namely Pakistan, India, and Afghanistan, are ranked among the lowest 10 countries in the world.

Europe: As a whole, Europe performed second only to North America on this issue and was one of the best-performing regions in the world in 2020. Every region in Europe has countries that performed among the best and among the worst on this issue. Among the 38 European countries listed in Table 21, nearly 90% (34) of the countries ranked in the top 50% in the world, with 39% (14) of the countries ranking in the top 20%, and 21% (8) of the countries ranking in the top 10%.

The performance of various regions in Europe on this issue is relatively balanced. For example, among the top 10 countries in Europe with the best performance on this issue, 4 (namely Russian, Ukraine, Poland, and Romania) are from Eastern Europe, 3 (namely Germany, France, and Netherlands) are from western Europe, 2 (namely Italy and Spain) are from Southern Europe, and 1 (namely the UK) is from northern Europe; while among the 10 worst-performing European countries, 5 (namely Slovenia, Albania, North Macedonia, Montenegro, and Malta) are from Southern Europe, 3 (namely Latvia, Estonia, and Iceland) are from Northern Europe, and the other two are from Eastern (Republic of Moldova) and Western (Luxembourg) Europe, respectively.

North America: North America as a whole performed the best in protecting women and children. The rankings of the two countries that make up North America remained the same as last year, with the United States ranking second in the world and Canada ranking sixteenth. It is noteworthy that, as with the public health issue, we used a population-weighted model to calculate countries’ scores on this issue. This means that a high ranking does not necessarily mean that the country leads all those that follow it in the ranking on this issue. This is not only true for countries that rank high, such as China and the United States, but also for countries that have low ranks, such as India and Bangladesh. In 2020, for example, the proportion of seats held by women in the national parliaments of the United States and Canada was nearly 29% and 27.5%, respectively, ranking sixty-first and fifty-second of the 163 countries in the ranking. For their part, the proportions for India and Pakistan were nearly 14.4% and 20.2%, respectively, ranking them 132nd and 101st in the world. Another example is the gender parity index for the gross enrollment ratio in primary and secondary education, which measures gender inequality in children’s education. The U.S. and Canada scored 0.98683 and 1.00259 on the indicators, respectively, just slightly above the global average (0.98564).

Latin America: Latin America as a whole performed well in protecting women and children. Of the 26 Latin American countries listed in Table 21, 2 countries (Brazil and Mexico) ranked fourth and fifth in the world, respectively; 19% (5 countries) were in the top 20% of the ranking, and 61.5% (16 countries) were in the top 50%. Guatemala ranked last among the Latin American countries, at 119th in the world, which is significantly higher than the rankings of many African and Asian countries.

The rankings of countries in the different regions of Latin America are relatively balanced on this issue. For example, the highest ranking country in the Caribbean region is Cuba, which ranks twenty-third in the world and fifth in Latin America, while the lowest ranked country is Saint Vincent and the Grenadines, ranking ninety-fourth in the world and twenty-third in Latin America. In the Central America region, the highest ranked country is Mexico, ranking fifth in the world and second in Latin America. By contrast, the lowest ranked country is Guatemala, at 119th in the world and 26th in Latin America. In the South America region, the highest ranked country is Brazil, ranking fourth in the world and first in Latin America, while the lowest ranked country is Paraguay, ranking ninety-sixth in the world and twenty-fifth in Latin America.

Africa: Africa as a whole is the worst-performing continent in terms of protecting women and children. Of the 42 African countries listed in Table 21, 89.1% (41 countries) ranked in the bottom 50% in the world on this issue, and 47.8% (22 countries) ranked in the bottom 20% in the world. In addition, 6 of the 10 countries that perform the worst in the world on this issue are in Africa. The top three African countries in terms of rank on this issue are Tunisia, South Africa, and Algeria, which rank thirty-third, forty-first, and forty-third in the world, respectively. However, their rankings do not enter the top 20% in the world.

Although all regions in Africa perform poorly in this issue from a global perspective, Southern Africa and Northern Africa (except Egypt) had among the better performances in the whole African continent. As mentioned earlier, the top three African countries in terms of rankings on this issue are in Southern Africa (South Africa) and North Africa (Tunisia and Algeria). By contrast, East Africa, Central Africa, and West Africa not only had the worst performance in Africa but also the worst performance in the world. Of the 10 countries that performed the worst in the world on this issue 5 are in these three regions, including Mozambique and Ethiopia in East Africa, Chad and Congo in Central Africa, and Nigeria in West Africa.

Oceania: In 2020, a total of eight countries in Oceania entered the ranking in the issue. Oceania as a whole performed better than Asia and Africa in protecting women and children but worse than Europe, North America, and Latin America. Australia and New Zealand are the two best-performing countries in Oceania, ranking eighteenth and fifty-sixth in the world, respectively. These rankings are not high compared with some countries in Europe, North America, and South America. However, the Solomon Islands and Palau are the two worst-performing countries in Oceania, ranking 105th and 131st in the world, respectively, a significantly higher rating than some of the underdeveloped countries in Asia and Africa.

From a regional perspective, the Australia and New Zealand region is the best-performing region in Oceania. The two highest ranking countries in Oceania both come from this region, followed by Polynesia. The Melanesia region is the worst-performing region in Oceania, with its four countries ranking the lowest in the continent as a whole. In addition, it is worth noting that all of the countries in Polynesia and Melanesia are ranked in the bottom 50% in the world, which indicates poor performance in this issue for these two regions.

#### Conclusion

Using a population-weighted model, we used 11 indicators from the World Bank and WHO relating to women’s health, women’s economic and political status, children’s health, and children’s education to assess each country’s performance in protecting women and children. Our results show that Europe, North America, and Latin America performed well on this issue. Oceania’s performance is in the middle. Although the performance of Asia as a whole is poor, there are significant differences in performance among different regions in Asia. For example, East Asian countries performed well on this issue, while South Asia is one of the worst-performing regions in the world. In addition, Africa is not only the worst-performing continent as a whole, but it also has poorly performing regions.

It should be noted that our results are based on a population-weighted model, so each country’s score on each indicator largely depends on two factors: (1) its relative position to the benchmark (i.e., the global weighted average) on that indicator, and (2) the size of the country’s population. The advantage of this approach is that we see all of humanity as a whole, and the extent to which a country contributes to justice for all of humanity depends not only on what it achieves for a given indicator, but also on the size of the population that it serves. Thus, our results show that the top- and bottom-ranking countries on the topic are both countries with large populations. As discussed, the highest ranking performance does not mean that the given country has the best performance on the topic; it may mean instead that it enabled a population that represents a significant portion of humanity as a whole to reach a level above the current global average. The converse is also true.

## Global Justice Indices: Main Results

This section presents the global justice rankings for 2020. As the results on the 10 issues show, the number of countries involved varied greatly for each issue. For example, climate change only involved 75 countries, whereas public health issue involved 190 countries. To prevent too many countries from being excluded from the Global Justice Index due to missing values, we adopt three strategies to construct the Global Justice Index. First, we construct the Global Justice Index by excluding the two issues with the most missing values, namely, climate change and anti-poverty. Doing this would allow the index to cover 149 countries (Table 22). Second, we exclude the climate change issue, which only relates to 75 countries in 2020, from the Global Justice Index (Table 23). Third, we report the Global Justice Index with all 10 issues (Table 24).

**Table 22:** Global justice index in 2020 (except for both climate change and anti-poverty)

Country	Ranking	Country	Ranking
United States of America	1	Uganda	76
Germany	2	Slovakia	77
United Kingdom of Great Britain and Northern Ireland	3	Jordan	78
China	4	Algeria	79
Sweden	5	Estonia	80
Canada	6	Tunisia	81
France	7	Colombia	82
Norway	8	Chad	83
Italy	9	Guatemala	84
Finland	10	Burkina Faso	85
Luxembourg	11	Dominican Republic	86
Belgium	12	Hungary	87
Switzerland	13	Trinidad and Tobago	88
Denmark	14	Ukraine	89
Brazil	15	El Salvador	90
Ireland	16	Benin	91
Spain	17	Mauritius	92
Australia	18	Suriname	93
Austria	19	Eswatini	94
Netherlands	20	Republic of Moldova	95
New Zealand	21	Botswana	96
Russian Federation	22	Namibia	97
Argentina	23	Sierra Leone	98
Republic of Korea	24	Kyrgyzstan	99
Iceland	25	Honduras	100
Israel	26	Kazakhstan	101
Saudi Arabia	27	Kenya	102
Uruguay	28	Cuba	103
India	29	Cameroon	104
Chile	30	Croatia	105
Greece	31	Oman	106
Portugal	32	Nicaragua	107
Mexico	33	Fiji	108
Costa Rica	34	Niger	109
South Africa	35	Pakistan	110
Bangladesh	36	Samoa	111
Philippines	37	Timor-Leste	112
Indonesia	38	Barbados	113
Panama	39	Burundi	114
Thailand	40	Serbia	115
Cyprus	41	Liberia	116
Poland	42	Cabo Verde	117
Egypt	43	Tajikistan	118
Malta	44	Cambodia	119
Paraguay	45	Mongolia	120
Czechia	46	Solomon Islands	121
Lesotho	47	Azerbaijan	122
Peru	48	Uzbekistan	123
Senegal	49	Albania	124
Turkey	50	Congo	125
Lithuania	51	Armenia	126
United Republic of Tanzania	52	Sri Lanka	127
Mozambique	53	Sao Tome and Principe	128
United Arab Emirates	54	Gambia	129
Latvia	55	Mali	130
Romania	56	Georgia	131
Nepal	57	Vanuatu	132
Iran (Islamic Republic of)	58	Guinea	133
Zambia	59	Guyana	134
Slovenia	60	Belize	135
Togo	61	Djibouti	136
Qatar	62	Maldives	137
Singapore	63	Viet Nam	138
Rwanda	64	Mauritania	139
Ghana	65	Central African Republic	140
Belarus	66	Tonga	141
Kuwait	67	Lao People’s Democratic Republic	142
Malawi	68	Nigeria	143
Bolivia (Plurinational State of)	69	Saint Lucia	144
Ecuador	70	Democratic Republic of the Congo	145
Malaysia	71	Saint Vincent and the Grenadines	146
Morocco	72	Myanmar	147
Ethiopia	73	Afghanistan	148
Madagascar	74	Bhutan	149
Bulgaria	75

**Table 23:** Global justice index in 2020 (excluding climate change)

Country	Ranking	Country	Ranking
United States of America	1	Ethiopia	66
China	2	Estonia	67
Germany	3	Dominican Republic	68
United Kingdom of Great Britain and Northern Ireland	4	Mauritius	69
Sweden	5	United Republic of Tanzania	70
France	6	Lesotho	71
Canada	7	Togo	72
Italy	8	Colombia	73
India	9	Uganda	74
Brazil	10	Republic of Moldova	75
Norway	11	Guatemala	76
Switzerland	12	Mozambique	77
Finland	13	El Salvador	78
Belgium	14	Zambia	79
Luxembourg	15	Chad	80
Spain	16	Kazakhstan	81
Austria	17	Azerbaijan	82
Denmark	18	Rwanda	83
Netherlands	19	Kyrgyzstan	84
Australia	20	Pakistan	85
Ireland	21	Croatia	86
Russian Federation	22	Fiji	87
Republic of Korea	23	Botswana	88
Greece	24	Namibia	89
Israel	25	Cameroon	90
Portugal	26	Serbia	91
Chile	27	Burkina Faso	92
Uruguay	28	Mongolia	93
Egypt	29	Honduras	94
Mexico	30	Samoa	95
Iceland	31	Malawi	96
Poland	32	Albania	97
Indonesia	33	Armenia	98
Thailand	34	Tajikistan	99
Bangladesh	35	Benin	100
Philippines	36	Sri Lanka	101
Cyprus	37	Eswatini	102
Turkey	38	Madagascar	103
Panama	39	Cabo Verde	104
Malta	40	Sierra Leone	105
Costa Rica	41	Georgia	106
Paraguay	42	Kenya	107
South Africa	43	Nicaragua	108
Lithuania	44	Gambia	109
Peru	45	Viet Nam	110
Czechia	46	Vanuatu	111
Latvia	47	Uzbekistan	112
Morocco	48	Mauritania	113
Romania	49	Timor-Leste	114
Malaysia	50	Liberia	115
Belarus	51	Solomon Islands	116
Nepal	52	Tonga	117
Slovenia	53	Niger	118
Ghana	54	Congo	119
Bulgaria	55	Guinea	120
Senegal	56	Sao Tome and Principe	121
Ukraine	57	Maldives	122
Jordan	58	Mali	123
Bolivia (Plurinational State of)	59	Burundi	124
Ecuador	60	Myanmar	125
Algeria	61	Lao People’s Democratic Republic	126
Iran (Islamic Republic of)	62	Nigeria	127
Hungary	63	Bhutan	128
Tunisia	64	Central African Republic	129
Slovakia	65	Democratic Republic of the Congo	130

**Table 24:** Global justice index in 2020 (including all 10 issues)

Country	Ranking	Country	Ranking
United States of America	1	Thailand	32
China	2	Philippines	33
Germany	3	Bangladesh	34
United Kingdom of Great Britain and Northern Ireland	4	Iceland	35
Sweden	5	Turkey	36
France	6	Cyprus	37
Canada	7	Peru	38
Italy	8	Lithuania	39
Brazil	9	Latvia	40
India	10	Czechia	41
Finland	11	Romania	42
Norway	12	Malaysia	43
Switzerland	13	South Africa	44
Belgium	14	Morocco	45
Luxembourg	15	Belarus	46
Spain	16	Slovenia	47
Austria	17	Bulgaria	48
Russian Federation	18	Ecuador	49
Denmark	19	Slovakia	50
Australia	20	Hungary	51
Ireland	21	Algeria	52
Netherlands	22	Ukraine	53
Republic of Korea	23	Estonia	54
Greece	24	Iran (Islamic Republic of)	55
Israel	25	Colombia	56
Portugal	26	Azerbaijan	57
Chile	27	Kazakhstan	58
Mexico	28	Pakistan	59
Egypt	29	Sri Lanka	60
Indonesia	30	Viet Nam	61
Poland	31	Uzbekistan	62

Table 22 reports the Global Justice Index following the exclusion of the two issues of climate change and anti-poverty, which cover 149 countries. The table shows that the top 10 countries in the ranking of this index are the United States, Germany, the UK, China, Sweden, Canada, France, Norway, Italy, and Finland. Of the top 10 countries, 7 are from Europe, 2 are from North America, and 1 is from Asia. Unlike the case of the top 10 countries in 2019, France as not included in 2019 due to missing values, but it replaced Belgium to enter the top 10 this year. There were no other changes among the other nine countries, except for minor fluctuations in their rankings.

**Fig. 11:**
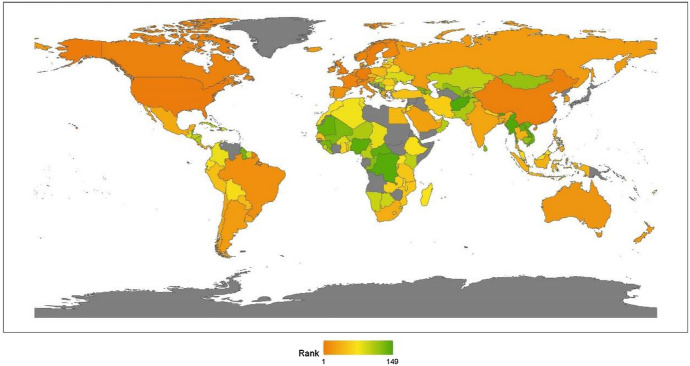
2020 Index Ranking of global justice (except for climate change and anti-poverty)

The bottom 10 countries for global justice in Table 22 are the Central African Republic, Tonga, Lao People’s Democratic Republic, Nigeria, Saint Lucia, Democratic Republic of the Congo, Saint Vincent and the Grenadines, Myanmar, Afghanistan, and Bhutan. Of these, four countries are from Asia, three are from Africa, two are from Latin America, and one is from Oceania. Like the top 10, the list of the bottom 10 countries remained relatively stable compared to the list in 2019, except for the new entries of Central African Republic, Nigeria, and the Democratic Republic of the Congo in 2020, while Congo, Georgia, and Vietnam escaped from this list. Figure 11 shows the index ranking of global justice that excludes climate change and anti-poverty in 2020.

Table 23 reports the Global Justice Index that excludes the issue of climate change in 2020, which covers 130 countries. As shown in the table, the top 10 countries in the Global Justice Index that exclude the issue of climate change are the USA, China, Germany, the UK, Sweden, France, Canada, Italy, India, and Brazil. Of the top 10 countries, 5 are in Europe, 2 are in Asia, 2 are in North America, and 1 is in Latin America. Compared to the top 10 countries of last year, India and Brazil are the new entrants to the top 10 this year. They rose from the twenty-fourth and eighteenth positions in 2019 to the ninth and tenth positions in 2020, replacing Norway and Belgium, which dropped from the sixth and eighth positions in 2019 to the eleventh and fourteenth positions in 2020, respectively.

**Fig. 12:**
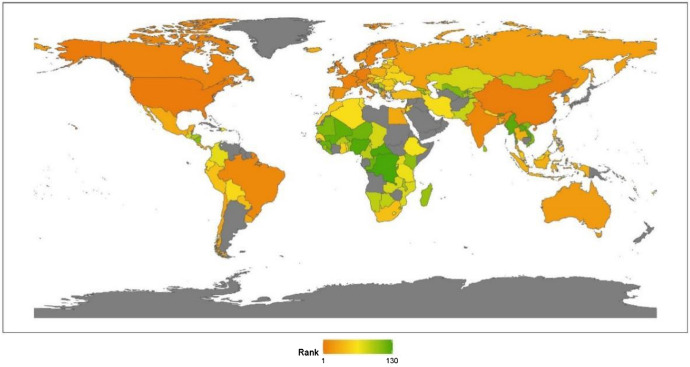
2020 Index ranking of global justice (except for climate change)

The bottom 10 countries in Table 23 are Sao Tome and Principe, Maldives, Mali, Burundi, Myanmar, Lao People’s Democratic Republic, Nigeria, Bhutan, Central African Republic, and Democratic Republic of the Congo. Of the bottom 10 countries, 5 are in Asia and the other 5 are in Africa. Compared to the bottom 10 countries in 2019, Sao Tome and Principe, Nigeria, Democratic Republic of the Congo, and Maldives are the new entrants to the bottom 10 this year. Among them, the first three were previously not ranked due to the lack of data, and Maldives previously ranked twentieth from the bottom. By contrast, Chad, Sierra Leone, Gambia, and Congo escaped from the bottom 10 countries this year, ranking 80th, 105th, 109th, and 119th, respectively. Figure 12 shows the index ranking of global justice that excludes anti-poverty in 2020.

Table 24 reports the Global Justice Index for 2020 that includes all 10 issues. Table 24 shows that the top 10 countries in the ranking are exactly the same as those in Table 23, namely the USA, China, Germany, the UK, Sweden, France, Canada, Italy, India, and Brazil. Relative to the top 10 countries in 2019, France, Brazil, and India are new entrants to the top 10 in 2020. Among them, France was not ranked due to the lack of data in for 2010, and Brazil and India rose from the seventeenth and twenty-third positions in 2019 to the ninth and tenth in 2020, respectively. By contrast, Norway, Finland, and Belgium dropped from the sixth, eighth, and tenth places in 2019 to the twelfth, eleventh, and fourteenth in 2020, falling out of the top 10.

**Fig. 13:**
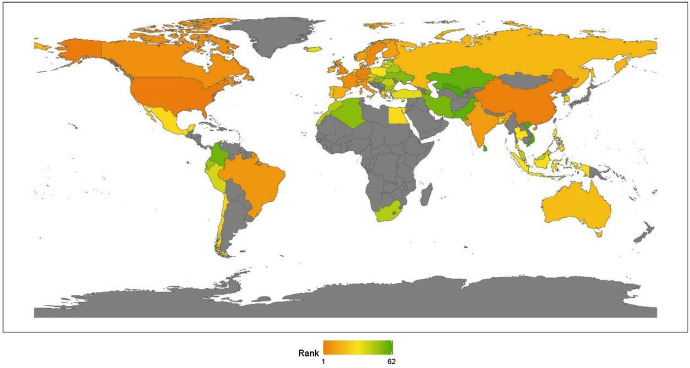
2020 Index ranking of global justice (including all ten issues)

The bottom 10 countries in Table 24 are Ukraine, Estonia, Iran (Islamic Republic of), Colombia, Azerbaijan, Kazakhstan, Pakistan, Sri Lanka, Viet Nam, and Uzbekistan. Of these seven are in Asia, two are in Europe, and one is in Latin America. Ukraine and Estonia dropped fell from eleventh and thirteenth from the bottom in 2019 to tenth and last ninth from the bottom, respectively, in 2020, replacing Algeria and Bangladesh as new entrants to the bottom 10 countries list. Figure 13 shows the index ranking of global justice that includes all 10 issues in 2020.

## Conclusion

The year 2020 was an extraordinary year for global justice. In the shadow of the COVID-19 pandemic, it has been a year of unprecedented shocks and challenges for many areas of global justice, including, but not limited to, public health, poverty governance, refugee governance, peacekeeping, and education.^96^ Our Global Justice Index in 2020 maintains the theoretical foundations, methodological framework, and measurement approaches used in previous years, on the one hand, and attempts to capture new changes in various issue areas and their respective implications for global justice, on the other.

For the theoretical part, we adhere to the two principles of CBDR-RC and CDDR, which synthesize rights-based, goods-based, and virtue-based approaches to understanding global justice and take into account the specificities of each issue area to provide a consistent and solid theoretical foundation for our measurements. In terms of the methodological framework and indicator measurement, we maintained the index construction method used previously, first selecting 10 issue areas to measure the influence of individual nation-states on global justice in each domain, forming separate sub-indices, and finally computing a unified global justice index through a synthetic formula. The 10 issue areas selected for the index were (1) climate change, (2) peacekeeping, (3) humanitarian aid, (4) terrorism and armed conflicts, (5) cross-national criminal police cooperation, (6) refugee, (7) anti-poverty, (8) education, (9) public health, and (10) the protection of women and children. These are key areas for defining, achieving and maintaining global justice, which nation-states can be expected to shoulder shared responsibilities and make meaningful contributions. In addition, we updated the indicator systems and data sources for relevant issue areas to reflect the latest developments (for instance, for the fields of humanitarian aid, public health, and education), while also maintaining continuity and consistency with previous years’ annual reports.

Assessing global justice is a challenging project. Given the limitations of data and for reasons of rigor, we report three versions of the index results: a global justice index including all the 10 issue areas (with a coverage of 62 countries); a global justice index without the issue area of climate change (with a coverage of 130 countries); and a global justice index without either of the issue areas of climate change or poverty (with a coverage of 149 countries). In the Global Justice Index for all the 10 issue areas, the top 10 countries, in order, are the United States, China, Germany, the United Kingdom, Sweden, France, Canada, Italy, Brazil, and India, which are closely followed by Finland, Norway, and Switzerland. The list has not changed much from 2019, except for the obvious rises in the rankings of Brazil and India. India’s ranking in the top 10 is driven primarily by its excellent performance in the issue areas of terrorism and armed conflict (due to the latest peace agreements), poverty governance, and climate change in 2020. Brazil’s rise in the ranking can largely be attributed to its progress in the areas of humanitarian aid and refugee governance. This is reflected in the growing activism of developing powers to take on the shared responsibility of global justice. The overall performance of top-ranked countries in the 10 issue areas is outstanding and sustainable, significantly enhancing global justice.

The final results suggest that many underdeveloped countries are underperforming due to their capacity constraints and poor endowments; however, there are also some developed countries that should contribute more and are also failing to put in their fair share of effort due to a lack of political will, self-interested motives, and domestic divisions. More importantly, our index reveals that, as a result of the pandemic, the influence of nation-states on global justice has become increasingly polarized in a number of issue areas, i.e., the gap between countries that perform well and those that do poorly is widening, including the domains of peacekeeping, education, refugees, poverty, and public health. All of these limit the progress of global justice in 2020. They also highlight once again the importance of mutual cooperation and shared governance among nation states.

The Global Justice Index tracks and measures the efforts and performance of nation states in promoting justice at a global level by focusing on 10 key issue areas of global governance and using more than 50 carefully-selected measurement indicators from highly respected sources. The index has several potential uses: (1) demonstrating the most recent performance and contribution of nation-states in global justice and its 10 key issue areas, it can serve to urge countries to pay additional attention to global justice and to undertake due-diligence obligations by taking more targeted and proactive actions in certain fields; (2) encouraging nation states to put aside their differences, end their self-serving political attitudes, and engage in more international and regional collaborations to jointly address various global governance challenges so that the light of justice can more equally shine on the world population; (3) calling on nation-states to share more data and on international organizations and academic institutions to explore the determining factors of global justice more scientifically by collecting, comparing, and studying relevant data and conducting more correlation and causality analyses, to establish data-driven policy decision-making and global governance.

Admittedly, the following caveats should be taken into account in reading and using the Global Justice Index: (1) due to missing data and limitations in the data imputation method, there may be some discrepancies between indicator measurements and real-world data in certain key issue areas, so potential updates to the data used should be borne in mind when using the index; (2) in the synthetic measurement of the final index, we assigned the same weight to all 10 issue areas, although the impact of different issue areas on global justice may not be exactly the same, because there is no theoretical basis for more precise weighting yet; (3) the index results are more suitable for making comparisons between countries within the same year. Cross-year comparisons require special attention to data coverage, as some ranking changes are simply caused by the problem of missing data. As a first attempt in this area, our index measurement is certainly not perfect, but we hope to keep improving as our research continues and as your feedback comes in.

## Data Availability

Data will be available upon request.

## Footnotes

1 Gu et al. (2020, 2021a, b, 2022a, b, c).

2 Kataria and Qu (2022).

3 Guo et al. (2019).

4 Guo et al. (2019).

5 Lu et al. (2018).

6 Betz and Coley (2021).

7 Mahmoud et al. (2018).

8 Ws et al. (2021).

9 NASA (2020).

10 See https://unfccc.int/sites/default/files/resource/1.GCP_pdf.

11 World Meteorological Organization (2021).

12 The 2030 Agenda for Sustainable Development, adopted by all United Nations Member States in 2015, provides a shared blueprint for peace and prosperity for people and the planet, now and into the future. At its heart are the 17 Sustainable Development Goals (SDGs), which are an urgent call for action by all countries—developed and developing—in a global partnership.

13 Guo et al. (2019).

14 Gu et al. (2022a, b, c).

15 IPCC (2021).

16 See https://www.epa.gov/ghgemissions/global-greenhouse-gas-emissions-data.

17 When measuring China's index results, we only used the data of China's mainland areas in our calculation. Therefore, Taiwan province was plotted as missing value on the map. This situation applies to all other visualization maps in this report.

18 Friedlingstein et al. (2020).

19 Cui et al. (2021).

20 Xing et al. (2021).

21 Wang et al. (2021).

22 Wang et al. (2020).

23 Liu et al. (2022).

24 IPCC (2021).

25 NASA (2020).

26 See https://unfccc.int/sites/default/files/resource/1.GCP_pdf.

27 Victoria and Glyn (2009).

28 Hegre et al. (2019).

29 Data source: https://www.start.umd.edu/gtd/.

30 Data source: https://ucdp.uu.se.

31 Sandler and Enders (2008).

32 See https://www.un.org/sg/en/content/sg/speeches/2020-03-23/secretary-general-appeal-for-global-ceasefire.

33 See https://www.unitar.org/sites/default/files/media/file/Factsheet%20CT%20Printed.pdf.

34 Marone (2022).

35 Data source: https://www.start.umd.edu/gtd/.

36 Data source: https://ucdp.uu.se.

37 Gu et al. (2021a, b).

38 See https://bti-project.org/en/reports/country-report/SDN.

39 Data source: https://ucdp.uu.se.

40 See https://pib.gov.in/Pressreleaseshare.aspx?PRID=1703510.

41 See https://www.mdn.gov.mm/en/myanmar-signs-union-accord-part-iii-establishing-democratic-federal-union.

42 Data source: https://www.start.umd.edu/gtd.

43 See http://ucdpged.uu.se/peaceagreements/fulltext/South%20Sudan%2020200112.pdf.

44 See https://www.worldbank.org/en/country/southsudan/overview#:~:text=Now%2C%20more%20than%20a%20decade,%2C%20displacement%2C%20and%20external%20shocks.

45 Data source: https://ucdp.uu.se.

46 Roser et al. (2021). Coronavirus pandemic (COVID-19). 2021. Our World in Data https://ourworldindata.org/coronavirus.

47 Institute for Economics & Peace (2022).

48 See https://www.state.gov/wp-content/uploads/2020/02/Agreement-For-Bringing-Peace-to-Afghanistan-02.29.20.pdf.

49 Data source: https://www.start.umd.edu/gtd.

50 Institute for Economics & Peace (2022).

51 Data source: https://ucdp.uu.se.

52 Bureau of Counterterrorism, Department of State, United States. (2021). Country Reports on Terrorism 2020.

53 Institute for Economics & Peace. (2022).

54 See https://www.who.int/emergencies/situations/humanitarian-crisis-in-sahel-region-of-africa#:~:text=region%20of%20Africa-,Overview,food%20insecurity%20and%20political%20instability.

55 Institute for Economics & Peace. (2022).

56 Institute for Economics & Peace. (2022).

57 UN High Commissioner for Refugees, Global Report 2020. https://www.unhcr.org/flagship-reports/globalreport/. Accessed on January 27, 2022.

58 Kluge et al. (2020).

59 See https://www.unhcr.org/protection/resettlement/600e95094/resettlement-fact-sheet-2020.html. Accessed on January 28, 2022.

60 Sujian et al. (2019).

61 See https://www.unhcr.org/news/press/2021/6/60ca09a74/unhcr-world-leaders-must-act-reverse-trend-soaring-displacement.html. Accessed on February 10, 2023.

62 Liu (2022a, b).

63 World Bank (2020).

64 Gu and Wang (2022).

65 Wang and Guo (2022), Zuo (2022) and Liu (2022a, b).

66 See Ferreira and María (2013).

67 See Fact Sheet: An Adjustment to Global Poverty Lines. https://www.worldbank.org/en/news/factsheet/2022/05/02/fact-sheet-an-adjustment-to-global-poverty-lines. Accessed on December 27, 2022.

68 This is mainly due to the fact that Vietnam's poverty headcount ratio decreased less in 2020 than that in 2019.

69 See the government report at http://www.gov.cn/xinwen/2020-12/03/content_5566565.htm. Accessed on December 29, 2022.

70 Assembly (1948).

71 Freeman (2022).

72 Yang et al. (2022) and Abid et al. (2021).

73 Doyle (2020).

74 Daniel (2020).

75 See https://databank.worldbank.org/.

76 See http://uis.unesco.org/.

77 See https://www.oecd.org/.

78 Al-Samarrai et al. (2021).

79 Data source: https://databank.worldbank.org/.

80 Gu et al. (2022a, b, c).

81 The Middle East in Asia includes 10 countries, with 9 in Western Asia (i.e., Oman, Saudi Arabia, Israel, Kuwait, United Arab Emirates, Jordan, Turkey, Qatar, and Lebanon) and 1 in Southern Asia (i.e., Iran).

82 See https://databank.worldbank.org/.

83 See https://databank.worldbank.org/.

84 See http://data.uis.unesco.org/.

85 See https://databank.worldbank.org/.

86 For instance, Meier (2006) and Tasioulas and Vayena (2020).

87 The concept of the right to health is recognized in various international human rights treaties and agreements, such as the Universal Declaration of Human Rights, the International Covenant on Economic, Social and Cultural Rights, and the Convention on the Rights of the Child (for instance, see, Leary (1994), Kinney (2000) and Toebes (2001)).

88 For instance, Ruger (2009) argues that local, national and global actors have duties to promote human flourishing and, more specifically, individuals’ central health capabilities. See Ruger (2009).

89 Office of the United Nations High Commissioner for Human Rights & World Health Organization. (2008). *The right to health*. Fact sheet no. 31. Leary (1994).

90 WHO (2020), Pradhan et al (2022) and Khan et al. (2021).

91 See https://github.com/CSSEGISandData.

92 The other one is from South America.

93 Carrington (2014).

94 Jaggar (2014) and Josefsson and Wall (2020).

95 Although some countries from southern Asia, such as Iran, rank relatively high in the world on this issue, it cannot change the fact that South Asia, as a whole, performs poorly.

96 It must be noted that the problem of climate change has been somewhat mitigated by the COVID-19 pandemic that impeded economic and social activities.
